# Synthesis of 3-Amino-4-(Hydroxymethyl)-β-Lactam Branch Point and Its Late-Stage C4 Functionalization with Ceftaroline-Sidechain Monobactams and Their Biological Evaluation

**DOI:** 10.3390/molecules31203637

**Published:** 2026-10-12

**Authors:** Vid Kavaš, Majda Golob, Anže Meden, Martina Hrast Rambaher, Stanislav Gobec

**Affiliations:** 1 Department of Pharmaceutical Chemistry, Faculty of Pharmacy, University of Ljubljana, Aškerčeva cesta 7, 1000 Ljubljana, Slovenia; vid.kavas@ffa.uni-lj.si (V.K.); anze.meden@ffa.uni-lj.si (A.M.); martina.hrast-rambaher@ffa.uni-lj.si (M.H.R.); 2 Institute of Microbiology and Parasitology, Veterinary Faculty, University of Ljubljana, Gerbičeva 60, 1000 Ljubljana, Slovenia; majda.golob@vf.uni-lj.si

**Keywords:** monobactam, 2-azetidinone, late-stage functionalization, penicillin-binding protein, molecular docking, click chemistry, antibacterial agents, antimicrobial resistance

## Abstract

Monocyclic β-lactams are an underexplored subclass of β-lactam antibiotics active against Gram-negative pathogens, but access to them requires long sequences reported across the patent and primary literature. We compare two alternative routes to 3-amino-4-(hydroxymethyl)-2-azetidinone, a synthon carrying handles at N1, C3 and C4, starting from ethyl glyoxylate and from (*R*)-glyceraldehyde acetonide. Both are assembled from published transformations; reported here are isolated yields for every step, including the conditions that failed, and execution of the β-lactam-forming sequence on a 50 g scale. Removal of the 2,4-dimethoxybenzyl group was strongly concentration-dependent, giving 94% on the glyoxylate-derived ester against 70% on the acetonide-derived alcohol, whereas the acetonide route replaces a low-yielding NaBH_4_ ester reduction (43 to 57%) with a near-quantitative oxidative cleavage/reduction sequence. Diverting one late-stage intermediate through copper-catalyzed azide–alkyne cycloaddition or direct thioether alkylation gave three sulfonated monobactams bearing a ceftaroline-derived C4 sidechain, in 0.10 to 0.29% overall yield over 15 steps. None reduced *S. pneumoniae* PBP1b activity below 50% at 100 μM, against an IC_50_ of 4.93 μM for aztreonam, and none inhibited wild-type *E. coli* or *S. aureus* at 128 μg/mL; weak inhibition was seen only in efflux-deficient and outer-membrane-permeabilized *E. coli*. C4 substitution through either linkage supports activity when the appended group also provides an uptake vector, which the ceftaroline-derived side chain does not, and it conferred no measurable target affinity either.

## 1. Introduction

Antimicrobial resistance (AMR) represents a rapidly escalating global health crisis, imposing a substantial burden on healthcare systems worldwide. In 2021 alone, AMR was directly responsible for approx. 1.14 million deaths and contributed to nearly 5 million fatalities, highlighting its impact across healthcare systems regardless of their economic status. Recognizing this threat, the World Health Organization has identified AMR as one of the top ten global health challenges and has emphasized the urgent need for new antibacterial strategies alongside improved stewardship and surveillance [1,2].

Bacterial peptidoglycan is an essential structural polymer of the bacterial cell wall and a key antibacterial target. The final step of its biosynthesis is catalyzed by penicillin-binding proteins (PBPs), which are D,D-transpeptidases responsible for peptide crosslinking via recognition of the D-Ala-D-Ala motif [3,4]. These enzymes are irreversibly inhibited by β-lactam antibiotics [5]. β-Lactam antibiotics remain among the most important antibacterial agents, although their clinical utility is increasingly compromised by resistance, most notably through β-lactamase expression. Their activity arises from structural mimicry of the D-Ala-D-Ala motif, enabling covalent inhibition of PBPs. Structurally, β-lactams comprise a diverse family defined by the β-lactam pharmacophore, with monobactams representing the only subclass lacking a fused bicyclic scaffold [6].

**Figure 1:**
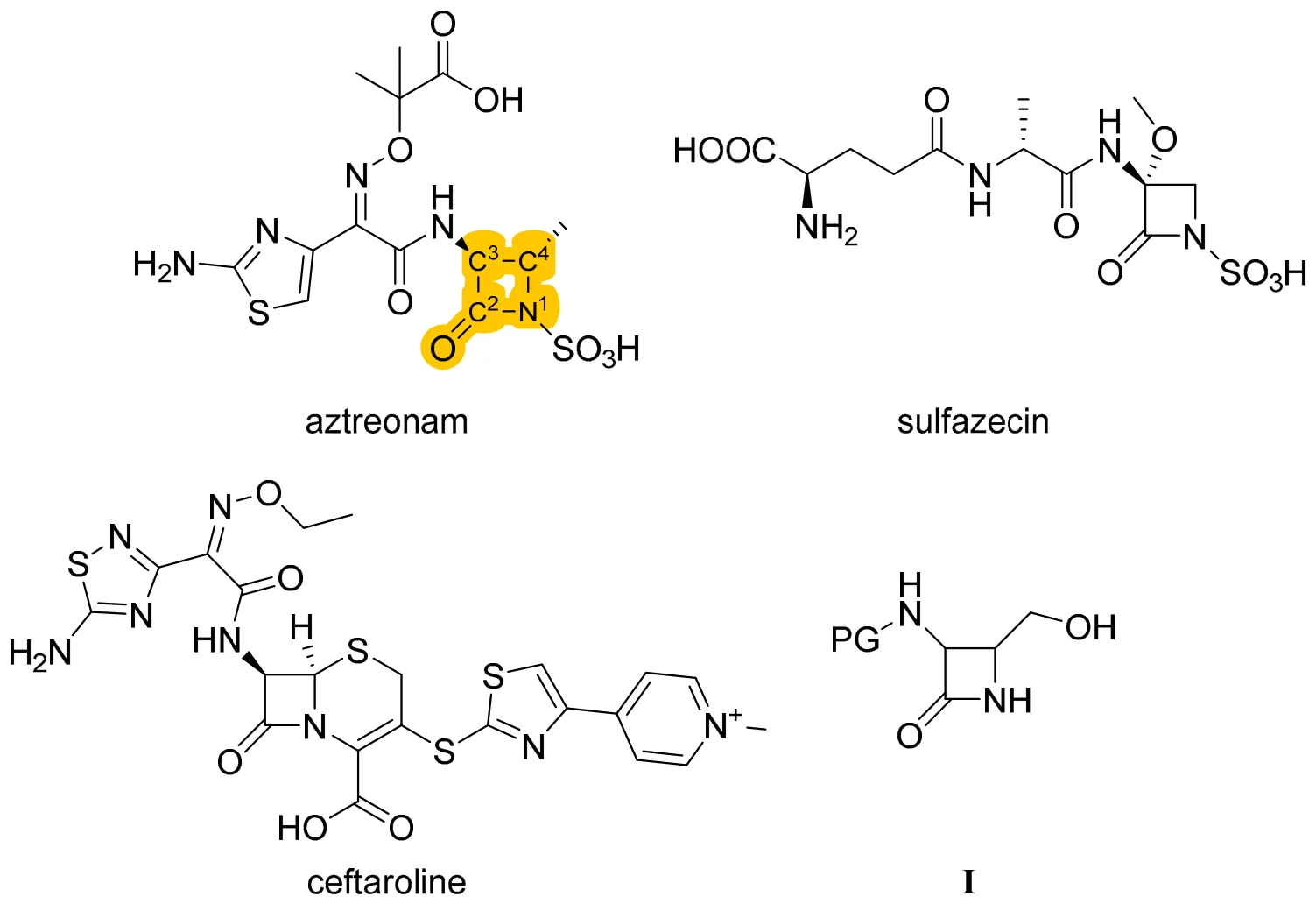
Structures of monobactams with 2-azetidinone core marked in orange and its numbering, and ceftaroline, PG—protective group.

The first monocyclic β-lactam antibiotic, sulfazecin (Figure 1), was reported in 1979, but its limited intrinsic activity necessitated further structural optimization [7]. This effort culminated in aztreonam, the first clinically approved monobactam [8]. In contrast to other β-lactams, aztreonam features a monocyclic scaffold bearing an N-sulfonated β-lactam, a motif critical for its biological activity. It exhibits selective inhibition of PBP3 in Gram-negative bacteria, resulting in potent activity against aerobic Gram-negative pathogens, while lacking efficacy against Gram-positive organisms [6,9].

Since the discovery of naturally occurring monocyclic β-lactams, significant effort has been directed toward the development of synthetic routes that enable structural diversification [10,11,12,13]. These strategies broadly fall into cycloadditions, cyclizations, and less conventional multicomponent or rearrangement-based approaches, as comprehensively reviewed by Bredael and co-workers [14,15]. Among them, the Staudinger [2 + 2] ketene–imine cycloaddition remains the most widely used method, enabling rapid assembly of the β-lactam core from readily accessible precursors via a nucleophilic addition–cyclization cascade [16]. Alternative approaches, including enolate–imine cycloadditions and rearrangement-based reactions, have been explored to access more highly decorated scaffolds [13,17,18,19,20]. Intramolecular cyclization strategies provide complementary access to monocyclic β-lactams, particularly through Mitsunobu-mediated closure or halogen-induced cyclization [21,22,23].

Functionalization at C3 and C4 of the monobactam core has been examined in detail. A route enabling late-stage diversification at both positions has been patented [24], and the key intermediate 3-amino-4-(hydroxymethyl)azetidin-2-one and several of its derivatives have been described previously, together with entries to it from methyl glyoxylate and from glyceraldehyde acetonide [25,26,27,28]. Conversion of the corresponding ester to the C4 iodide has been reported in higher yield than obtained here [29], and the C4 azide has been prepared under a different protecting-group regime [30]. At C4 itself, thioether substitution has given monobactams active against Gram-negative bacteria in both *cis* and *trans* series [9], and triazole linkages installed by copper-catalyzed azide–alkyne cycloaddition have given active *cis*-configured compounds with linkers ranging from direct attachment of the aryl group to extended variants [25]. The synthetic approaches to 3-amino-β-lactams bearing heteroatom-containing C4 substituents have been reviewed recently [14]. In the reported series, activity is associated with C4 substituents that also promote uptake, such as siderophores and iron-chelating pyridones [9,25]. The chemistry described here therefore rests on established transformations. What is reported is a side-by-side comparison of the two entries with isolated yields for every step, including the conditions that failed, execution of the β-lactam-forming sequence on a 50 g scale, and the use of a single late-stage intermediate to access two different C4 linkage types.

Despite this diversity of methods, the synthesis of monobactams remains challenging. Many routes require multistep sequences, prefunctionalized substrates, or careful control of reactive intermediates, which collectively limit scalability and rapid diversification. As a result, the development of modular and efficient strategies for the construction and late-stage functionalization of monocyclic β-lactams remains an important objective, particularly at the C3 and C4 positions, which are critical for tuning biological activity. Herein we report two alternative routes to the 3-amino-4-(hydroxymethyl)azetidin-2-one synthon **I**, which carries handles for functionalization at N1, C3 and C4, and their use to prepare three N1-sulfonated monobactams bearing a ceftaroline-derived side chain at C4. The 2-(pyridin-4-yl)-1,3-thiazol-5-yl group of ceftaroline, a side chain that contributes to the penicillin-binding protein binding profile of that cephalosporin [31], was selected in order to test whether it would enhance affinity when transferred to C4 of a monobactam. PBP1b from *Streptococcus pneumoniae* was used as the model enzyme, the same species for which ceftaroline’s PBP affinities have been characterized. The *N*-methylated pyridinium form present in ceftaroline was prepared alongside the non-methylated analogues to probe whether the additional fixed positive charge would improve passage across the Gram-negative outer membrane, an approach distinct from the iron-chelating vectors used in the published C4-substituted series. The enzymatic assay was the primary readout and the antibacterial testing a secondary check. The compounds were evaluated for inhibition of PBP1b from *S. pneumoniae* and against wild-type, efflux-deficient and outer-membrane-permeabilized strains.

## 2. Results and Discussion

### 2.1. Chemistry

**Scheme 1:**
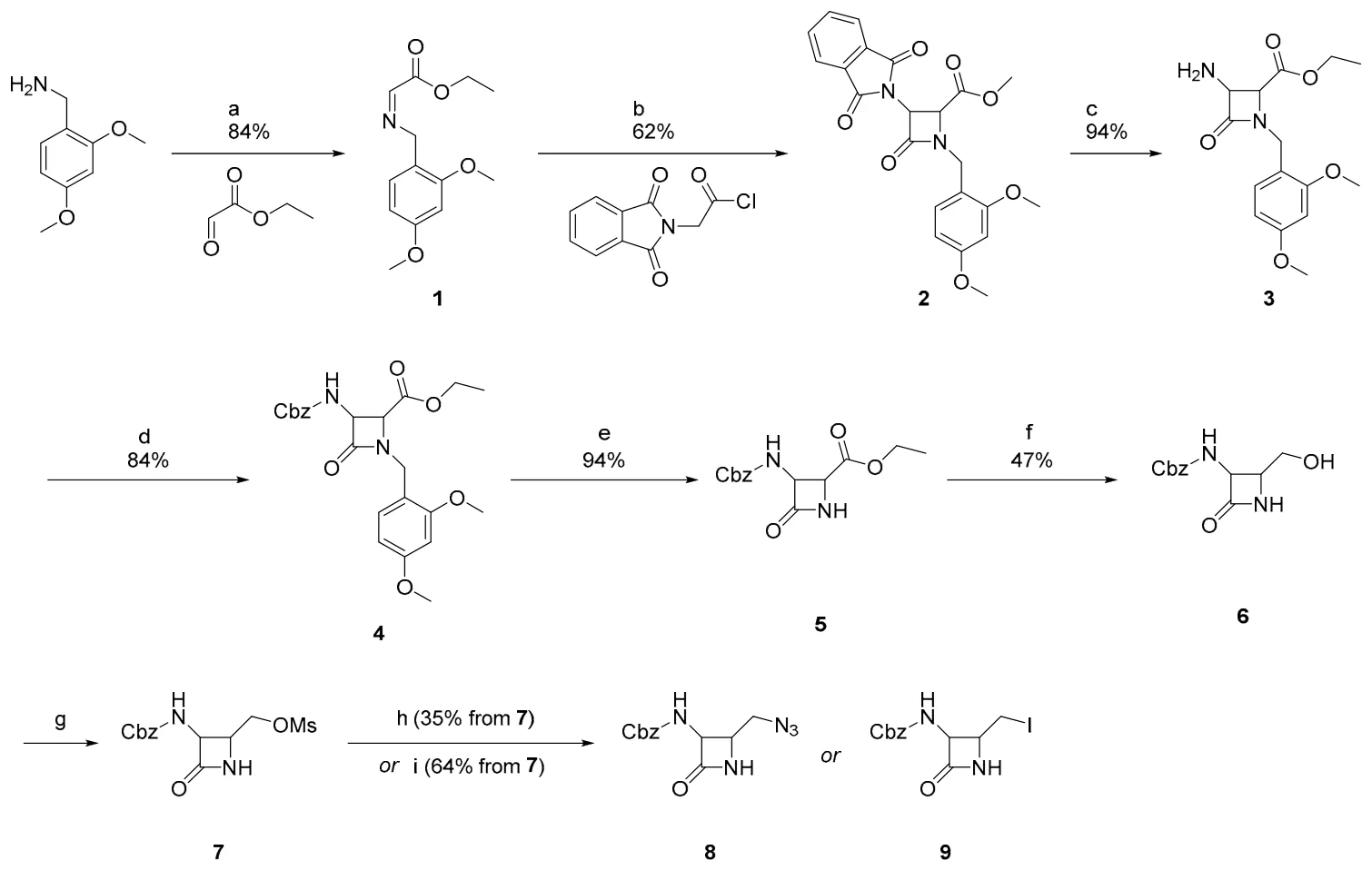
Synthetic route starting with ethyl glyoxylate. a—ethyl glyoxylate, MgSO_4_, dichloromethane (DCM), argon, rt, 4 h; b—(1.) 2-(1,3-dioxoisoindolin-2-yl)acetyl chloride, Et_3_N, DCM, argon, 0 °C, 2 h, (2.) rt, 16 h; c—methyl hydrazine, DCM, argon, 35 °C, 16 h; d—(1.)benzyl chloroformate (CbzCl), *N*,*N*-diisopropylethylamine (DIPEA), DCM, argon, 0 °C, 30 min, (2.) rt, 2 h; e—K_2_S_2_O_8_, K_2_HPO_4_, MeCN/water, 95 °C, 3 h; f—(1.) NaBH_4_, THF/water, argon, 0 °C, 1 h, (2.) rt, 16 h; g—(1.) MsCl, Et_3_N, DCM, 0 °C, 15 min, (2.) rt, 3 h; h—NaN_3_, DIPEA, dimethylformamide (DMF), rt, 48 h; i—NaI, acetone, reflux, 16 h.

**Scheme 2:**
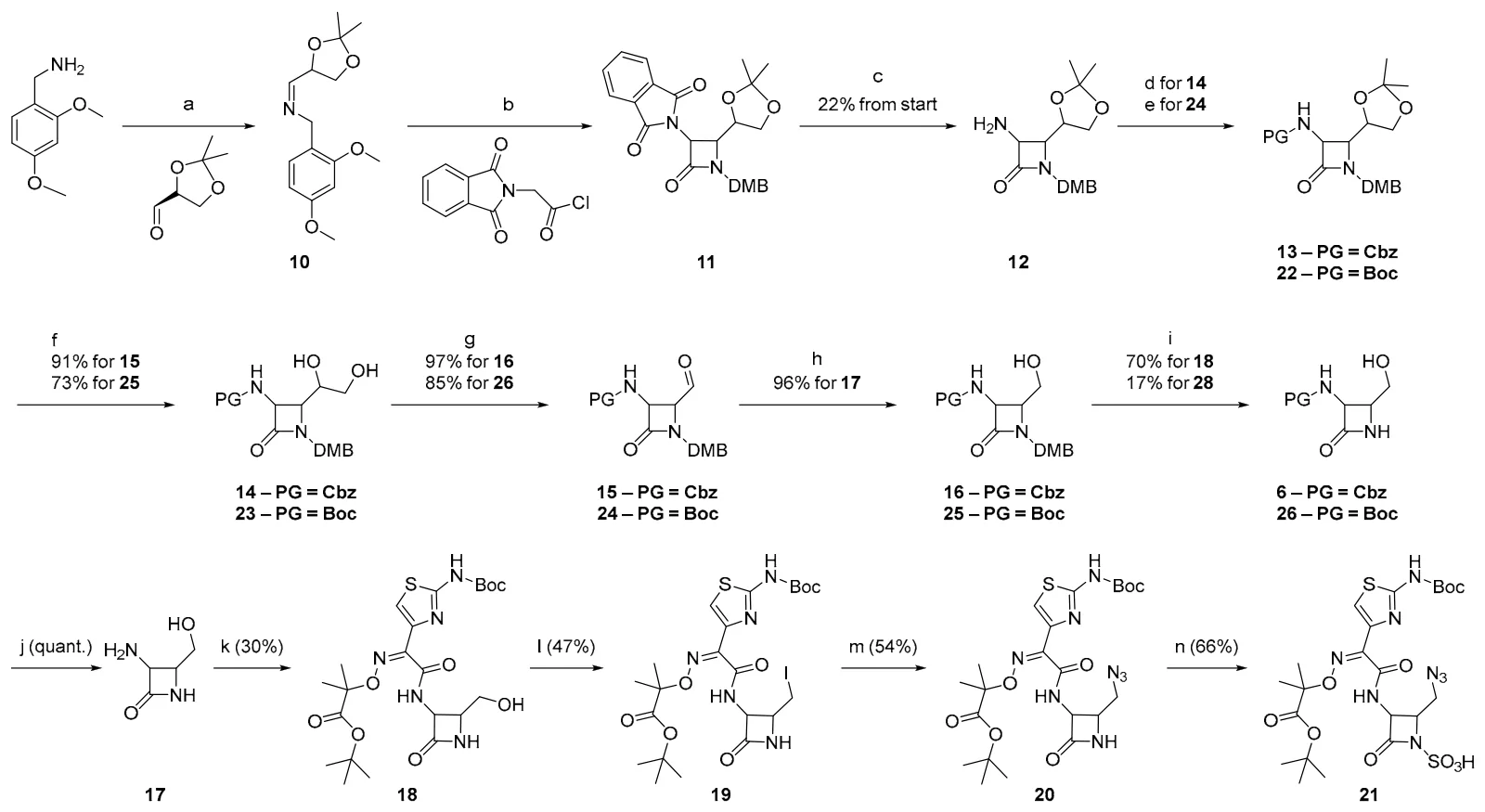
Glyceraldehyde acetonide synthetic route. a—(*R*)-2,2-dimethyl-1,3-dioxolane-4-carbaldehyde, MgSO_4_, DCM, rt, 16h; b—(1.) 2-(1,3-dioxoisoindolin-2-yl)acetyl chloride, Et_3_N, DCM, 0 °C, argon, 2 h, (2.) rt, 16 h; c—hydrazine hydrate, DCM, rt, 3 h; d—(1.) CbzCl, NaHCO_3_, ethyl acetate (EA), 0 °C, (2.) rt, 2 h; e—di-*tert*-butyl dicarbonate (Boc_2_O), argon, THF, rt, 16 h; f—*p*TsOH, THF/water, 70 °C, 16 h; g—NaIO_4_, EA/water, 50 °C, 2 h; h—NaBH_4_, DCM/MeOH, 0 °C, 2 h; i—K_2_S_2_O_8_, K_2_HPO_4_, MeCN/water, 95 °C, 4 h; j—H_2_, Pd/C, acetic acid (AcOH), MeOH, rt, 8 h; k—*tert*-butyl (*Z*)-2-(((2-(benzo[d]thiazol-2-ylthio)-1-(2-((*tert*-butoxycarbonyl)amino)thiazol-4-yl)-2-oxoethylidene)amino)oxy)-2-methylpropanoate (**20b**), DIPEA, DMF, 0 °C → rt, 16 h; l—Ph_3_P, imidazole, I_2_, rt, 16 h; m—tetrabutylammonium azide (TBAN_3_), Et_3_N, THF, rt, 16 h; n—DMF·SO_3_, DMF, rt, 4 h.

Two alternative synthetic routes toward target intermediate **I** (Figure 1) were explored, each starting from a different aldehyde precursor. The first, outlined in Scheme 1, commences from ethyl glyoxylate [26]; the second, presented in Scheme 2, employs glyceraldehyde acetonide as the starting material [24,27,28]. Both converge on the same Cbz-protected alcohol **6**, from which all subsequent chemistry proceeds. *Cis* and *trans* C4-substituted monobactams show activity [9,25], and the Staudinger conditions used here give the cis isomer predominantly.

In the synthetic route, starting from ethyl-glyoxylate (Scheme 1), the key intermediates **4** and **5** contain an ethyl ester; however, attempts at basic hydrolysis to the corresponding acid were unsuccessful. Cbz protecting group was evaluated, and the sequence of transformations was adjusted to minimize unnecessary protection–deprotection steps, with the goal of improved yields. Ethyl glyoxylate was prepared from diethyl D-tartrate using periodic acid-mediated cleavage and purified by vacuum distillation. Due to its rapid polymerization, the crude product was used immediately for imine formation. This step required anhydrous conditions and the presence of magnesium sulfate drying agent in the reaction mixture was essential, as residual water and water released during imine formation significantly reduced conversion. On larger scale, mild heating (35 °C) was applied to accelerate formation of the imine **1**. Construction of the β-lactam core (**2**) was achieved following established procedures [26], with the product isolated by crystallization from ethyl acetate. Subsequent deprotection of the phthalimide group using methylhydrazine afforded the free amine **3**, which was then protected as a Cbz derivative (**4**) to enable orthogonal downstream transformations.

Removal of the 2,4-dimethoxybenzyl (DMB) group, a widely used protection strategy in monobactam synthesis [12], from the β-lactam nitrogen was performed using potassium persulfate oxidation at elevated temperature, yielding compound **5**. This step proved highly concentration-dependent: dilution led to negligible conversion, whereas excessive concentration on a larger scale resulted in diminished yields (Table 1).

Reduction of the ester to the corresponding alcohol was achieved using sodium borohydride (2–8 eq.) in aqueous THF, with 2 equivalents sufficient for full conversion (43–57% yield), as shown in Table 2. The sequence of ester reduction and DMB deprotection was found to significantly influence overall yield. Performing reduction prior to DMB removal improved the reduction yield (up to 74% crude yield) but led to poor deprotection efficiency. Conversely, DMB removal prior to reduction afforded moderate overall yields. Mesylate activation of the Cbz-protected alcohol **6** gave the azide **8** and the iodide **9** in 35% and 64% yield, respectively. These two compounds are included because they establish the C4 branch point while C3 still carries only the Cbz group: an azide suitable for copper-catalyzed cycloaddition and an iodide suitable for nucleophilic displacement are both accessible before the C3 side chain is installed, so the order in which C3 and C4 are addressed is interchangeable. Neither was advanced to a final compound, the glyoxylate route having been superseded by the acetonide route described below, and the final compounds were instead prepared from the corresponding ATMO-substituted iodide **19** and azide **20**. The conversion of ester **5** to iodide **9** by the same sequence of reactions has been reported previously in higher yield [25]. Cbz removal by hydrogenolysis then allows the C3 side chain to be introduced, followed by N1 sulfonation, with any remaining protecting groups removed in the final step and purification by reverse-phase chromatography.

The synthetic route starting from glyceraldehyde acetonide roughly followed previously published procedures as shown in Scheme 2 [24]. The route was developed from commercially available (*R*)-2,2-dimethyl-1,3-dioxolane-4-carbaldehyde, avoiding the need for glyoxylate preparation and vacuum distillation, while also addressing the low efficiency of ester reduction observed in the glyoxylate-based approach described above. The same DMB-protected amine and phthaloyl glycine were employed, with variation introduced only at the aldehyde component during β-lactam formation. In addition, this route circumvents the use of some outdated or hazardous reagents reported in earlier procedures, for example, distillation of ethyl glyoxylate from tetrahydrofuran, and use of methylhydrazine and propylene oxide.

Following β-lactam formation, phthalimide deprotection was achieved using hydrazine hydrate, and the resulting amine **12** was reprotected with either Cbz (**13**) or Boc (**22**) groups. Subsequent acetonide cleavage under acidic conditions (*p*-toluenesulfonic acid) afforded diols **14** and **23**—for the latter, a partial deprotection was observed and was corrected after confirming acetonide removal with the addition of di-*tert*-butyl dicarbonate and DIPEA) (Scheme 2). The resulting diols were subjected to oxidative cleavage with sodium periodate, followed by reduction to the corresponding primary alcohols (**16** and **25**) using sodium borohydride (DCM/MeOH, 0 °C). These transformations proceeded in near-quantitative yields in both routes.

DMB deprotection proceeded less efficiently than in the glyoxylate route (Scheme 1) for analogous substrates, with notably higher yields observed for the Cbz-protected derivative **6** (70%) compared to the Boc analogue **26** (17%). Removal of the DMB group from **16** gave the alcohol **6**, identical to the material obtained through the glyoxylate route (Scheme 1). The downstream chemistry therefore proceeds from a single intermediate and is independent of which route delivered it: mesylate activation gives the azide **8** and the iodide **9**, while Cbz removal gives **17** and opens the sequence leading to **18** to **21**. Subsequent deprotection of the Cbz group afforded intermediate **17**, which was functionalized at C3 with the protected (*Z*)-2-((((2-aminothiazol-4-yl)(carboxy)methylene)amino)oxy)-2-methylpropanoic acid (ATMO) side chain, present in aztreonam. The primary alcohol was then converted to the corresponding iodide via an Appel reaction, providing a key diversifiable intermediate **19**. In the initial reaction, iodide **19** was transformed into the corresponding azide (**20**), followed by β-lactam sulfonation (using SO_3_·DMF) to **21**.

**Scheme 3:**
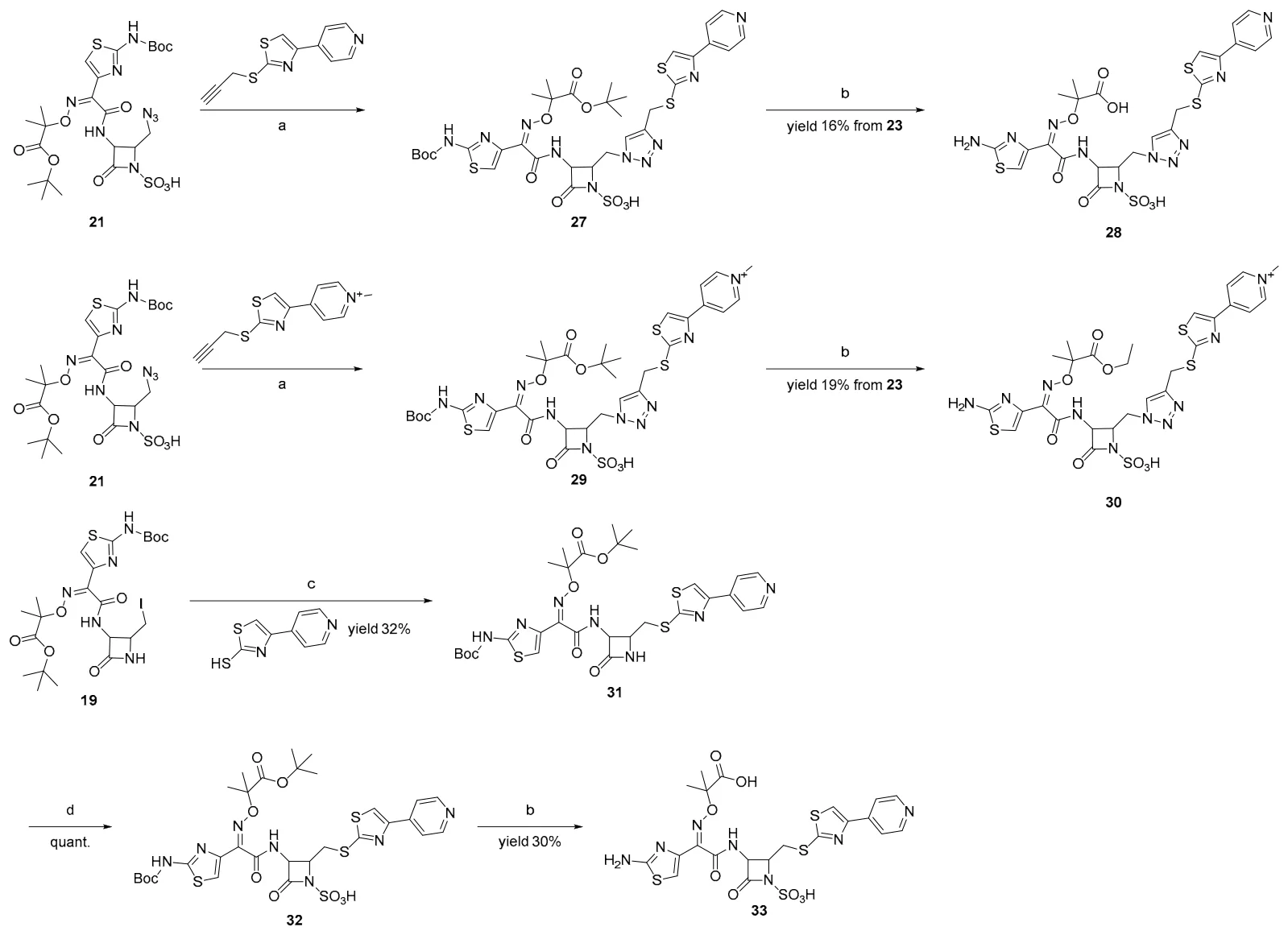
Synthesis of final compounds through C4 modification. a—sodium ascorbate, CuSO_4_, MeCN, rt, 16 h; b—TFA, DCM, rt, 16 h; c—K_2_CO_3_, MeCN, 80 °C, 16 h; d—DMF·SO_3_, DMF, rt, 4 h.

From this point forward, two diversification pathways were explored (Scheme 3). Click reaction with alkyne-functionalized (*N*-methylated or non-methylated) ceftaroline side chains and azide **21** was performed affording **27** and **29**. Final deprotection with TFA afforded products **28** and **30**. In the second approach, direct alkylation of non-methylated ceftaroline-derived thiol was performed at C4 with iodide **19** prior to β-lactam sulfonation (**32**), followed by global deprotection (TFA/DCM) to yield product **33**. Notably, in this pathway, the introduction of the C4 substituent occurs prior to the sulfonation of β-lactam, in contrast to the click-based route. An additional advantage of this path is the ability to access distinct C4 linkages, enabling installation of either thioether or triazole functionalities. This dual approach expands the accessible chemical space and provides enhanced flexibility for tuning the physicochemical and biological properties of the resulting monobactam derivatives.

The sequence is not efficient in absolute terms, and this is stated plainly here. Compounds **28**, **30** and **33** were obtained in 0.10 to 0.29% overall yield over a longest linear sequence of 15 steps from (*R*)-2,2-dimethyl-1,3-dioxolane-4-carbaldehyde, which corresponds to an average of roughly 63 to 68% per step. The low overall figure is therefore the cumulative product of a long sequence rather than the consequence of a single poor transformation. Among the steps examined systematically leading to the common Cbz-protected alcohol **6**, removal of the DMB group is the largest documented loss in the acetonide route (70%, Table 1) and is strongly concentration dependent, while in the glyoxylate route the NaBH_4_ reduction of the ester did not exceed 57% under any conditions tested (Table 2) and was one reason that route was not carried forward. The value of the sequence does not lie in its overall yield. It lies in the fact that a single late-stage intermediate can be diverted to either a triazole or a thioether at C4, that the order of reduction, deprotection and functional group interconversion can be varied so that the same intermediate is reachable by more than one path, and that every step is reported with an isolated yield, including the conditions that failed.

### 2.2. Docking Study

To rationalize the design of the novel C4-functionalized monobactams and assess their potential for PBP1b inhibition prior to synthesis, a molecular docking study was performed. Compounds differing in C4 attachment style (triazole and thioether) were evaluated against PBP1b from *S. pneumoniae* (PDB ID: 9SGB). Two complementary docking protocols were employed: SCAR docking [32]—in which the active-site serine was mutated to alanine and a positional constraint was applied to place the β-lactam ring in proximity to the catalytic residue—thereby isolating the non-covalent component of binding, and covalent docking, in which both the pre- and post-reaction binding poses were evaluated.

**Figure 2:**
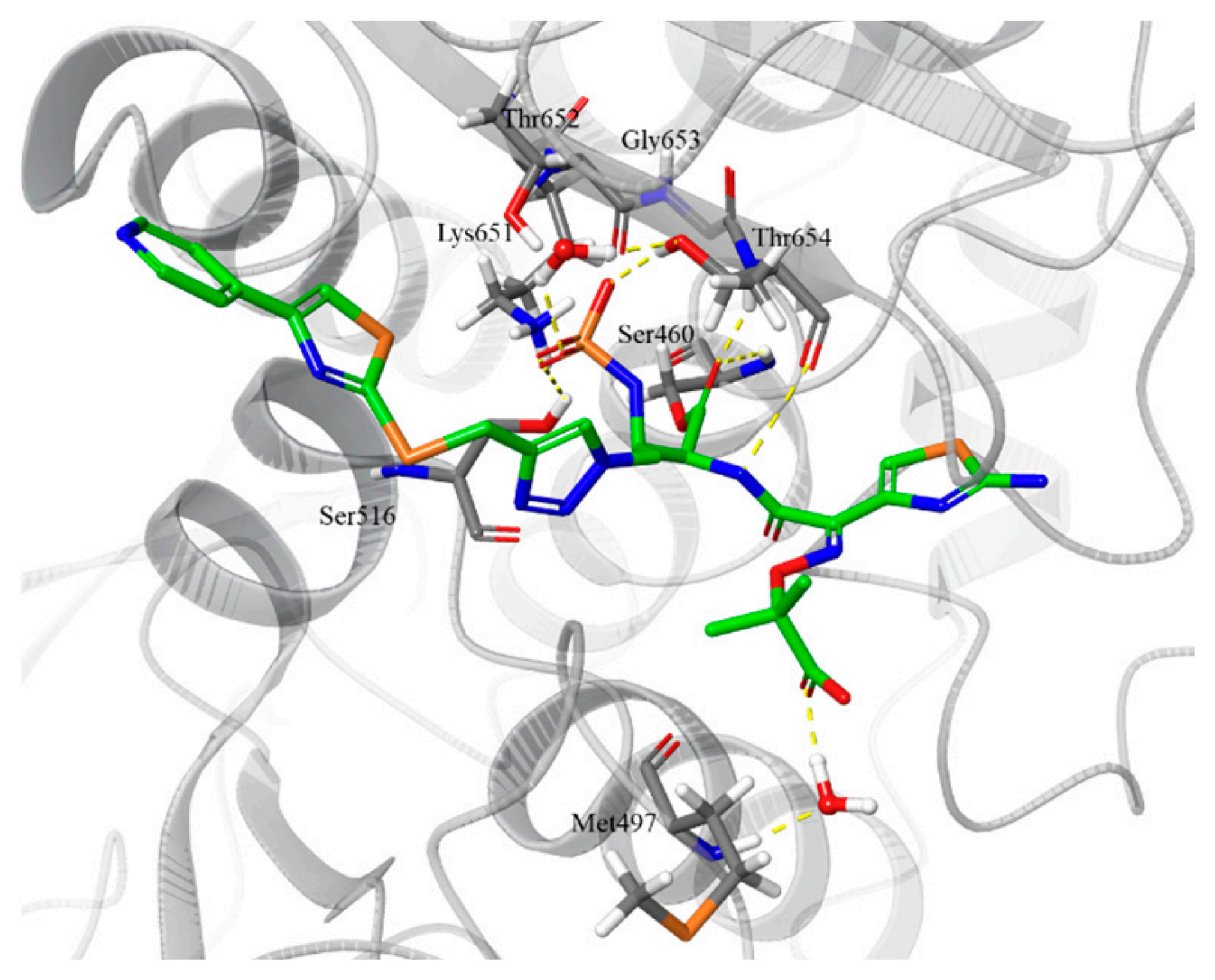
Covalent docking pose of compound **28** in PBP1b* from *S. pneumoniae* with amino acid residues forming interactions shown. Yellow dashed lines represent hydrogen bonds.

In the SCAR docking workflow, the permanently charged, *N*-methylated triazole-linked compound **30** ranked highest, with a docking score of −10.05, forming nine hydrogen bonds and an additional salt bridge between the charged nitrogen and Glu561. Thioether-linked compound **33** ranked second (score −9.85), forming six hydrogen bonds and a salt bridge between the sulfonic acid moiety and Lys651, with no additional interactions from the sidechain. The non-methylated triazole-linked compound **28** ranked third (score −9.50), forming seven hydrogen bonds in total, including one between the sidechain and Asn492. For reference, aztreonam scored −7.83, with seven hydrogen bonds originating predominantly from the β-lactam ring atoms and the N1-sulfonic acid. Covalent docking using a maximum common substructure approach produced a different ranking (Figure 2). Compound **28** ranked highest (score −9.30), forming five hydrogen bonds and a covalent linkage to Ser460. Compound **33** ranked second (score −8.80), with six hydrogen bonds and the covalent linkage, followed by compound **30** (score −8.50), forming two hydrogen bonds alongside the covalent bond to Ser460. Aztreonam again ranked lowest among the evaluated compounds, scoring −7.21 with four hydrogen bonds and the covalent linkage to Ser460.

### 2.3. Enzyme Inhibition Study

The seven compounds, comprising the non-sulfonated β-lactam **19** and six N1-sulfonated analogues (**21**, **27**, **28**, **30**, **32** and **33**), were assayed against PBP1b from *S. pneumoniae*, used here as an accessible model transpeptidase (Table 3). Aztreonam was assayed under identical conditions, including the same 30 min preincubation, and inhibited the enzyme with an IC_50_ of 4.93 μM, which confirms that the assay reports inhibition by this chemotype.

None of the seven compounds reduced residual enzyme activity below 50% at 100 μM after 30 min preincubation. The most active was the thioether-linked **33** (55% residual activity), followed by **28** (70%). Their IC_50_ values therefore exceed 100 μM, corresponding to a loss of potency of at least 20-fold relative to aztreonam. The non-sulfonated **19** showed no inhibition (100% residual activity), consistent with the requirement for N1 sulfonation and serving as an internal negative control.

The docking scores did not predict these results. Aztreonam, the only compound with measurable inhibition, ranked last under both protocols (SCAR −7.83; covalent −7.21), whereas the inactive **30** ranked first under the SCAR protocol (−10.05). The two protocols ranked the three final compounds differently. Both protocols score the non-covalent component of a pre-acylation pose and therefore do not report the acylation rate, which governs inhibition by a β-lactam. The calculations were used to prioritize which C4 linkage to prepare, and we do not present them as evidence of binding.

C4 thioether monobactams are active in both *cis* and *trans* series [9,14], C4 triazole-linked monobactams are active on a *cis* scaffold with linkers ranging from direct attachment of the aryl group to elongated variants [25,33], and both linkage types therefore occupy a position that tolerates aryl substitution. Neither the linkage chemistry, nor the linker length, nor the *cis* configuration can by itself account for the loss of potency observed here. What distinguishes the present compounds is the identity of the C4 substituent: a rigid 2-(pyridin-4-yl)-1,3-thiazol-5-yl group taken from the C3′ position of ceftaroline, transplanted to a position two bonds from the β-lactam ring, where its exit vector differs from that in the cephem scaffold.

### 2.4. Antibacterial Activity

Minimum inhibitory concentrations (MICs) were determined for the same seven compounds against *S. aureus* ATCC 29213 and *E. coli* ATCC 25922 as wild-type Gram-positive and Gram-negative strains, and against two *E. coli* mutants with compromised barriers to compound entry, the efflux-deficient strain N43 (*acrA*) and the outer-membrane-permeabilized strain D22 (*lpxC/envA1*) (Table 3). The mutant strains were included to establish whether the outer membrane and efflux limit exposure of the target to these compounds. None of the compounds inhibited growth of either wild-type strain at concentrations up to 128 μg/mL. Against *E. coli* N43, the thioether-linked **33** and the permanently charged, *N*-methylated triazole **30** gave MICs of 64 μg/mL and the non-methylated triazole **28** gave 128 μg/mL, while intermediates **19**, **21**, **27** and **32** remained inactive. Against *E. coli* D22, **30** and **33** gave MICs of 128 μg/mL and all other compounds were inactive. Aztreonam, included as a monobactam comparator, gave MICs of 0.5 μg/mL against each of *E. coli* ATCC 25922, N43 and D22.

Growth inhibition was therefore detected only in the strains with impaired efflux or increased outer-membrane permeability, which is consistent with limited outer-membrane penetration and/or efflux. Intracellular accumulation was not measured, and the enzymatic data indicate that low intrinsic affinity for the target also contributes. Neither the *N*-methylated **30** nor the non-methylated **28** and **33** inhibited growth of the wild-type strains, and the single dilution step separating **30** from **28** in the compromised strains is within the reproducibility of the method, so the effect of the additional fixed positive charge cannot be assessed from this set. The published C4-substituted monobactams place these results in context. In the C4 thioether series, compounds bearing a siderophore are substantially more active than the corresponding thioether carrying only an aminoethyl chain, which is nearly inactive [9], and the C4 triazole-linked monobactams that reach Gram-negative targets carry a pyridone that acts as an iron-chelating uptake vector [25]. In both series, antibacterial activity therefore tracks with the presence of an uptake vector rather than with the linkage type or the point of attachment. The ceftaroline-derived side chain used here was selected as a binding element rather than as an uptake vector, and the absence of activity against the wild-type strains is consistent with that precedent. The enzymatic data show that it did not function as a binding element either. In the C4 thioether series, the siderophore-bearing analogue (compound 42 in ref. [9]) inhibited *P. aeruginosa* PAO1 at 0.4 μM and *E. coli* DC0 at less than 0.025 μM in iron-depleted medium, whereas the matched compound carrying only an aminoethyl thioether (compound 39) was more than 125-fold and more than 240-fold less potent, respectively (greater than 50 μM and 6 μM) [9].

## 3. Materials and Methods

### 3.1. PBP1b Purification

We employed a vector expressing PBP1b from *S. pneumoniae* (designated pGEX-GST-PBP1b) to transform chemically competent *E. coli* NiCo21(DE3) (obtained from New England Biolabs, Ipswich, MA, USA), following previously established protocols [34,35]. The transformed cells were cultured at 37 °C and 250 rpm in LB broth supplemented with 100 µg/mL ampicillin until reaching an OD_600_ of approximately 1. Expression of PBP1b was induced by adding 1 mM IPTG, and the culture was further incubated at 16 °C for an additional 20 h. After induction, cells were harvested by centrifugation (10 min at 3000× *g*, 4 °C), and the resulting cell pellets were stored at –80 °C for subsequent purification steps. To purify PBP1b, the cell pellet was resuspended in buffer A (composed of 50 mM Tris-HCl, 200 mM NaCl, 1 mM EDTA, 1 mM DTT, pH 8.0) and lysed using sonication on ice. Cell debris was removed by centrifugation (30 min at 16,000× *g*, 4 °C, repeated twice). The cleared lysate was loaded onto two interconnected 1-mL GSTrap HP columns (Cytiva, Marlborough, MA, USA), which had been pre-equilibrated with buffer A. Following loading, the column was washed with buffer A, and the protein of interest (PBP1b) was eluted using buffer B (containing 50 mM Tris, 200 mM NaCl, 1 mM EDTA, 1 mM DTT, 10 mM reduced glutathione, pH 8.0). The eluted PBP1b was subsequently transferred to buffer C (composed of 50 mM HEPES, 100 mM NaCl, 1 mM EDTA, and 10% glycerol, pH 7.0) through buffer exchange. Finally, the purified protein was concentrated using a 50-kDa molecular weight cut-off filter (Ultra-4 centrifugal filter units; Amicon, Darmstadt, Germany), aliquoted, flash-frozen in liquid nitrogen, and stored at –80 °C. Protein purity was checked by SDS-PAGE, and the concentration was determined fluorometrically with Invitrogen Qubit (Thermo Fisher, Waltham, MA, USA).

### 3.2. PBP1b Inhibition Assay

We investigated the inhibition of *S. pneumoniae* PBP1b using an assay with BOCILLIN FL, as reported previously [35]. To measure fluorescence anisotropy, we employed 60 nM purified PBP1b and 30 nM BOCILLIN FL in a 100 mM sodium phosphate buffer (pH 7.0) containing 0.01% Triton X-100. The inclusion of Triton X-100 helped reduce promiscuous inhibitor detection and protein binding to the plate. The assay was conducted in triplicate, using 50 μL volumes in black flat-bottom, 384-well microplates at 30 °C. We quantified the change in fluorescence anisotropy using a Biotek Synergy H4 Hybrid microplate reader equipped with polarizing filters (excitation wavelength λ = 482 nm, emission wavelength λ = 530 nm). The calculated fluorescence anisotropy (FA) followed this equation: (FA) = (F_para_ − F_perp_)/(F_para_ + 2F_perp_), where (F_para_) represents the fluorescence intensity parallel to the excitation plane, and (F_perp_) represents the fluorescence intensity perpendicular to the excitation plane. Additionally, we determined residual activities by preincubating the test compound (100 µM) and the protein for 30 min at 30 °C before initiating the reaction with BOCILLIN FL. To assess residual activity, we compared the change in FA after 30 min to the uninhibited (1% *v*/*v* DMSO) control. IC_50_ values were determined for aztreonam under the conditions described above, including the same 30-min preincubation, across seven inhibitor concentrations. Residual activities were fitted to a four-parameter non-linear regression model in GraphPad Prism 10.5.0 (GraphPad Software, San Diego, CA, USA). Values are reported as means with standard deviations. IC_50_ values were not determined for the test compounds, none of which reduced residual activity below 50% at 100 μM.

### 3.3. Bacterial Susceptibility Test

Minimum inhibitory concentrations were determined using the broth microdilution method in 96-well U-bottom microtiter plates according to the guidelines of the Clinical and Laboratory Standards Institute (CLSI) and the European Committee on Antimicrobial Susceptibility Testing (CLSI M07 and The European Committee on Antimicrobial Susceptibility Testing. Breakpoint tables for interpretation of MICs and zone diameters. Version 16.1, 2026. https://www.eucast.org, accessed on 27 May 2026). Bacterial strains included *S. aureus* (ATCC 29213), *E. coli* (ATCC 25922), *E. coli* (N43, CGSC no. 5583) and *E. coli* (D22, CGSC no. 5163). Following overnight cultivation on the blood agar, bacterial suspensions equivalent to the 0.5-McFarland turbidity standard were prepared and subsequently diluted in cation-adjusted Mueller-Hinton broth supplemented with TES (Thermo Fisher Scientific, USA) to obtained a final inoculum of 5 × 10^5^ CFU/mL. The compounds, dissolved in DMSO, were mixed with the bacterial inoculum and incubated at 35 °C for 18–24 h (2.5% final DMSO concentration). The MIC values were determined visually as the lowest compound concentration at which no visible bacterial growth was observed. Tetracycline was included as a positive control and 2.5% DMSO as growth control on each test plate, and all experiments were performed in duplicate.

### 3.4. Molecular Docking

Docking studies were conducted using Glide [36] with a 5 Å constraint to mutated Ala460 and default settings using standard precision (Schrödinger Suite 2024-2, Schrödinger, LLC, New York, NY, USA) on monobactam-bound complex with penicillin-binding protein 1b* (PBP1b*) from *Streptococcus pneumoniae* (PDB ID: 9SGB [37]) with either active site serine mutated to Ala for SCAR docking or not for covalent docking. Protein preparation was carried out using the Protein Preparation Wizard. Hydrogen atoms were added, protonation states were assigned at pH 7.4, and the hydrogen-bonding network was optimized. Water molecules located more than 3.0 Å from heteroatoms were removed, followed by restrained energy minimization. The receptor grid was centered on the co-crystallized ligand.

Aztreonam (fixed *S*,*S* configuration) was used as a control. Monobactam analogues bearing ceftaroline-derived side chains were docked with both possible *cis* stereochemical configurations. Covalent docking was performed using CovDock [38] using maximum common substructure with bound ligand and default settings (Schrödinger Suite 2024-2).

### 3.5. Chemistry

The reagents and solvents were used as received from commercial suppliers. After extraction, organic phases were dried over anhydrous sodium sulfate. Reactions were monitored using analytical thin-layer chromatography (TLC) on silica gel 60 F_254_ Al plates. Developed plates were inspected under UV light and, if necessary, visualized with ninhydrin or phosphomolybdic acid. Nuclear magnetic resonance spectra were recorded on a Bruker Avance III 400 MHz spectrometer at 400 MHz for ^1^H, 101 MHz for ^13^C nucleus, respectively, using CDCl_3_, DMSO-*d*_6_, acetone-*d*_6_, or D_2_O with TMS as the internal standard as solvent. Chemical shifts are reported in parts per million (ppm), TMS peak was calibrated to 0 ppm or, alternatively, the central peak of the residual solvent resonance was used as the internal reference. The multiplicities are reported as follows: s (singlet), d (doublet), t (triplet), q (quartet), m (multiplet), dd (doublet of doublets), ddd (doublet doublet of doublets), td (triplet of doublets), qd (quartet of doublets), and br (broad), number of equivalent nuclei (by integration), coupling constants (*J*) quoted in Hertz (Hz). Mass spectra were recorded on Thermo Scientific Q Exactive Plus LC–MS/MS. Column chromatography was performed on silica gel (Silica gel 60, particle size: 0.035–0.070 mm, Merck, Darmstadt, Germany). UPLC analyses were performed on Thermo Scientific Dionex UltiMate 3000 modular system (Thermo Fisher Scientific Inc.). The general method used a Waters Acquity UPLC^®^ HSS C18 SB column (2.1 mm × 50 mm, 1.8 μm) thermostated at 40 °C, with: injection volume, 1 μL; sample concentration, 0.1–0.2 mg/mL in MeOH; flow rate, 0.4 mL/min; detector λ, 220 and 254 nm; mobile phase A: 0.1% TFA (*v*/*v*) in MilliQ water (Merck Millipore); mobile phase B: MeCN. Gradient: 0–7 min, 10–100% B; 7–8 min, 10% B.

### 3.6. Synthetic Procedures

#### 3.6.1. Synthesis of Ethyl-2-((2,4-dimethoxybenzyl)imino)acetate (**1**) [26]

To a solution of diethyl D-tartrate (50 g, 0.242 mol, 1.0 eq.) in diethyl ether (450 mL) cooled to 0 °C under an argon atmosphere was added periodic acid (55.3 g, 0.242 mol, 1.0 eq.) portionwise over 1 h. The reaction mixture was stirred for an additional 12 h at 0 °C, then dried over Na_2_SO_4_. The solids were removed by filtration, and the solvent was evaporated under reduced pressure. The crude product was purified by vacuum distillation according to a previously reported procedure, which yielded a light yellow liquid (42 g, 86%) [39]. The crude product ethyl glyoxylate **1a** was used in the next step without further purification.

To a solution of (2,4-dimethoxyphenyl)methanamine (62.6 mL, 0.414 mol, 1.0 eq.) in dichloromethane (DCM, 500 mL) was added anhydrous MgSO_4_ (100 g), followed by compound **1a** (1 eq.). The reaction mixture was stirred under an argon atmosphere for 4 h at room temperature. The drying agent was removed by filtration, and the solvent was evaporated under reduced pressure. The residue was co-evaporated with toluene to afford a red viscous liquid (87.5 g, 84%). ^1^H NMR (400 MHz, CDCl_3_) δ 1.34 (t, *J* = 7.1 Hz, 3H), 3.77 (s, 3H), 3.80 (s, 3H), 4.32 (q, *J* = 7.2 Hz, 2H), 4.82 (d, *J* = 1.8 Hz, 2H), 6.43–6.50 (m, 2H), 7.11 (d, *J* = 8.4 Hz, 1H), 7.58 (t, *J* = 1.8 Hz, 1H).

#### 3.6.2. Synthesis of Ethyl 1-(2,4-Dimethoxybenzyl)-3-(1,3-dioxoisoindolin-2-yl)-4-oxoazetidine-2-carboxylate (**2**)

To a solution of phthaloyl glycine (50 g, 0.244 mol, 1.0 eq.) in dichloromethane (DCM, 250 mL) and *N*,*N*-dimethylformamide (DMF, 1 mL) cooled to 0 °C was added oxalyl chloride (41.8 mL, 0.487 mol, 2.0 eq.) portion wise and the gas that has formed was removed under reduced pressure using a water pump. The reaction mixture was stirred at room temperature for 18 h under an argon atmosphere. The solvent was removed under reduced pressure to afford the crude product 2-(1,3-dioxoisoindolin-2-yl)acetyl chloride **2a**, which was used in the next step without further purification. To a solution of compound **1** (87.5 g, 0.348 mol, 1.0 eq.) in dichloromethane (DCM, 300 mL), triethylamine (96.5 mL, 0.696 mol, 2.0 eq.) was added portion wise. The reaction mixture was cooled to 0 °C, and a solution of crude **2a** (77.86 g, 0.348 mol, 1.0 eq.) in DCM (200 mL) was added dropwise. The reaction was then stirred at room temperature for 16 h.

The mixture was washed successively with 1 M HCl (250 mL), water (250 mL), saturated aqueous NaHCO_3_ (250 mL), and brine (250 mL). The organic phase was dried over Na_2_SO_4_, filtered, and concentrated under reduced pressure. The crude product was crystallized from ethyl acetate (EA, 350 mL) to afford orange crystals (94.6 g, 62%). ^1^H NMR (400 MHz, CDCl_3_) δ 1.05 (t, *J* = 7.1 Hz, 3H), 3.79 (d, *J* = 2.7 Hz, 6H), 3.98 (dq, *J* = 10.7, 7.1 Hz, 1H), 4.09 (dq, *J* = 10.7, 7.1 Hz, 1H), 4.18 (d, *J* = 6.0 Hz, 1H), 4.38 (d, *J* = 14.3 Hz, 1H), 4.90 (d, *J* = 14.3 Hz, 1H), 5.46 (d, *J* = 6.0 Hz, 1H), 6.41–6.46 (m, 2H), 7.17 (d, *J* = 7.9 Hz, 1H), 7.69–7.75 (m, 2H), 7.78–7.86 (m, 2H); LC-MS: *m*/*z* (M + H)^+^ calculated 439.2, found 438.9.

#### 3.6.3. Synthesis of Ethyl 3-Amino-1-(2,4-dimethoxybenzyl)-4-oxoazetidine-2-carboxylate (**3**)

To a solution of compound **2** (30.69 g, 70.0 mmol, 1.0 eq.) in DCM (300 mL) under an argon atmosphere was added methylhydrazine (11.06 mL, 210 mmol, 3.0 eq.). The reaction mixture was heated to 35 °C and stirred for 8 h. The solvent was then removed under reduced pressure, and the residue was dissolved in EA (200 mL) and washed with 1 M HCl (2 × 60 mL). The combined aqueous layers were cooled to 0 °C, neutralized with saturated aqueous NaHCO_3_, and extracted with DCM (3 × 100 mL). The combined organic extracts were dried over Na_2_SO_4_, filtered, and concentrated under reduced pressure to afford a yellow viscous liquid (20.32 g, 65.9 mmol, 94%). ^1^H NMR (400 MHz, CDCl_3_) δ 1.24 (t, *J* = 7.1 Hz, 3H), 3.73 (s, 3H), 3.76 (s, 3H), 4.05 (d, *J* = 5.1 Hz, 1H), 4.14 (d, *J* = 14.3 Hz, 1H), 4.16–4.21 (m, 2H), 4.33 (d, *J* = 5.1 Hz, 1H), 4.51 (d, *J* = 14.3 Hz, 1H), 6.37–6.41 (m, 2H), 7.06 (d, *J* = 8.9 Hz, 1H); ^13^C NMR (101 MHz, CDCl_3_) δ 14.4, 39.9, 55.2, 55.4, 60.6, 61.3, 63.6, 98.4, 104.2, 115.5, 131.5, 158.7, 161.0, 169.0, 169.6; LC-MS: *m*/*z* (M + H)^+^ calculated 309.1, found 308.7.

#### 3.6.4. Synthesis of Ethyl 3-(((Benzyloxy)carbonyl)amino)-1-(2,4-dimethoxybenzyl)-4-oxoazetidine-2-carboxylate (**4**)

To a solution of compound **3** (20.32 g, 65.9 mmol, 1.0 eq.) in dichloromethane (DCM, 200 mL) was added *N*,*N*-diisopropylethylamine (DIPEA, 16.8 mL, 98.9 mmol, 1.5 eq.), and the reaction mixture was cooled to 0 °C. Benzyl chloroformate (CbzCl, 11.3 mL, 79.1 mmol, 1.2 eq.) was then added dropwise. The reaction was stirred at room temperature for 2 h, after which the solvent was removed under reduced pressure. Diethyl ether (160 mL) was added to the residue, and the mixture was stored at 4 °C for 48 h. The resulting light yellow crystals were collected by filtration (24.61 g, 84%). ^1^H NMR (400 MHz, CDCl_3_) δ 1.16 (t, *J* = 7.1 Hz, 3H), 3.76 (s, 3H), 3.80 (s, 3H), 4.06–4.22 (m, 3H), 4.56 (d, *J* = 14.3 Hz, 1H), 5.08 (s, 2H), 5.21–5.30 (m, 1H), 5.33 (d, *J* = 9.2 Hz, 1H), 6.40–6.46 (m, 2H), 7.07–7.11 (m, 1H), 7.30–7.35 (m, 5H).

#### 3.6.5. Synthesis of Ethyl 3-(((Benzyloxy)carbonyl)amino)-4-oxoazetidine-2-carboxylate (**5**)

To a solution of compound **4** (24.61 g, 55.62 mmol, 1.0 eq.) in a mixture of water/MeCN (1:2, 834 mL) were added potassium persulfate (21.05 g, 77.87 mmol, 1.4 eq.) and K_2_HPO_4_ (12.59 g, 72.31 mmol, 1.3 eq.). The reaction mixture was heated at 95 °C for 3 h, and the progress was monitored by TLC. After completion, MeCN was removed under reduced pressure, and the aqueous phase was extracted with ethyl acetate (3 × 100 mL). The combined organic layers were washed with saturated aqueous NaHCO_3_ (200 mL) and brine (100 mL), dried over Na_2_SO_4_, filtered, and concentrated under reduced pressure. The crude product was purified by column chromatography on silica gel (hexane, H/EA = 1:1) and subsequently recrystallized from diethyl ether to afford off-white crystals (15.22 g, 94%). ^1^H NMR (400 MHz, CDCl_3_) δ 1.17 (t, *J* = 7.1 Hz, 3H), 4.10–4.22 (m, 2H), 4.43 (d, *J* = 5.3 Hz, 1H), 5.06–5.15 (m, 2H), 5.42 (dd, *J* = 9.7, 5.3 Hz, 1H), 5.68 (d, *J* = 9.7 Hz, 1H), 6.30 (s, 1H), 7.31–7.38 (m, 5H); LC-MS: *m*/*z* (M + H)^+^ calculated 292.1, found 292.5.

#### 3.6.6. Synthesis of Benzyl (2-(Hydroxymethyl)-4-oxoazetidin-3-yl)carbamate (**6**)

Compound **5** was dissolved in a mixture of THF/water (1:1, 210 mL) and cooled to 0 °C. Sodium borohydride (6.37 g, 168.4 mmol, 4.0 eq.) was added portion wise, and the reaction mixture was stirred at room temperature for 16 h. The reaction was then cooled to 0 °C and quenched with saturated aqueous citric acid. THF was removed under reduced pressure, and the aqueous phase was extracted with ethyl acetate (3 × 100 mL). The combined organic layers were washed with saturated aqueous NaHCO_3_ (2 × 50 mL), dried over Na_2_SO_4_, filtered, and concentrated under reduced pressure. The crude product was purified by column chromatography on silica gel (DCM/acetone = 3:1) to afford the product as a white solid (4.95 g, 47%). ^1^H NMR (400 MHz, Acetone-*d*_6_) δ 3.66–3.77 (m, 1H), 3.84–3.94 (m, 2H), 4.23 (s, 1H), 5.06–5.14 (m, 3H), 6.71 (d, *J* = 10.0 Hz, 1H), 7.23 (s, 1H), 7.29–7.43 (m, 5H); ^13^C NMR (101 MHz, Acetone-*d*_6_) δ 55.2, 60.8, 61.7, 67.2, 128.8, 128.9, 129.3, 138.1, 156.8, 168.6; LC-MS: *m*/*z* (M + H)^+^ calculated 250.1, found 250.6.

Alternatively, to a solution of compound **17** (13.46 g, 33.6 mmol, 1.0 eq.) in MeCN (336 mL) was added potassium persulfate (12.73 g, 47.1 mmol, 1.4 eq.), followed by a solution of dipotassium hydrogen phosphate (K_2_HPO_4_, 7.62 g, 43.7 mmol, 1.3 eq.) in water (168 mL). The resulting mixture was heated to 95 °C and stirred for 4 h, with progress monitored by TLC. Subsequently, additional potassium persulfate (2.73 g, 10.1 mmol, 0.3 eq.) and K_2_HPO_4_ (1.76 g, 10.1 mmol, 0.3 eq.) were added, and the reaction was heated for a further 2 h. After completion, the reaction mixture was cooled to room temperature and concentrated under reduced pressure to remove most of the MeCN. The residue was extracted with EtOAc (3 × 150 mL), and the combined organic layers were washed with water (100 mL) and brine (100 mL), dried over Na_2_SO_4_, filtered, and concentrated under reduced pressure. The crude product was purified by silica gel column chromatography (DCM/acetone = 10:1 → 1:3) to afford the title compound as a white solid (5.89 g, 70%). ^1^H NMR (400 MHz, CDCl_3_) δ 2.78 (s, 1H), 3.72 (d, *J* = 12.1 Hz, 1H), 3.91 (d, *J* = 9.9 Hz, 2H), 5.11 (s, 2H), 5.21 (dd, *J* = 9.7, 5.0 Hz, 1H), 6.03 (d, *J* = 9.7 Hz, 1H), 6.27 (s, 1H), 7.31–7.39 (m, 5H); ^13^C NMR (101 MHz, Acetone-*d*_6_) δ 55.1, 60.7, 61.6, 67.1, 128.8, 128.8, 129.3, 138.0, 156.7, 168.7.

#### 3.6.7. Synthesis of (3-(((Benzyloxy)carbonyl)amino)-4-oxoazetidin-2-yl)methyl Methanesulfonate (**7**)

Compound **6** (550 mg, 2.19 mmol, 1.0 eq.) was dissolved in DCM (20 mL), and DIPEA (745 μL, 4.38 mmol, 2.0 eq.) was added. The reaction mixture was cooled to 0 °C, and methanesulfonyl chloride (MsCl, 204 μL, 1.2 eq.) was added dropwise. The reaction was stirred under argon atmosphere at room temperature for 3 h, and the progress was monitored by TLC. The organic phase was washed with saturated aqueous NaHCO_3_ (20 mL), dried over Na_2_SO_4_, filtered, and concentrated under reduced pressure. The crude product was used directly in subsequent steps without further purification. ^1^H NMR (400 MHz, Acetone-*d*_6_) δ 3.10 (s, 3H), 4.11–4.17 (m, 1H), 4.37–4.48 (m, 2H), 5.11 (s, 2H), 5.19 (ddd, *J* = 9.4, 5.2, 1.4 Hz, 1H), 7.17 (d, *J* = 9.5 Hz, 1H), 7.30–7.43 (m, 5H), 7.67 (s, 1H); LC-MS: *m*/*z* (M + H)^+^ calculated 328.1, found 328.6.

#### 3.6.8. Synthesis of Benzyl (2-(Azidomethyl)-4-oxoazetidin-3-yl)carbamate (**8**)

Compound **7** (0.65 g, 1.99 mmol, 1.0 eq.) was dissolved in DMF (10 mL), then DIPEA (1.04 mL, 5.97 mmol, 3.0 eq.) and NaN_3_ (130 mg, 1.99 mmol, 1.0 eq.) were added. The reaction mixture was stirred at 60 °C for 70 h under an argon atmosphere. DMF was removed under reduced pressure, and the residue was taken up in DCM and washed with water (2 × 10 mL) and brine (10 mL). The organic phase was dried over Na_2_SO_4_, filtered, and concentrated under reduced pressure. The crude product was purified by column chromatography on silica gel (DCM/acetone = 10:1) to afford the product as an orange viscous liquid (193 mg, 35% from **6**). ^1^H NMR (400 MHz, CDCl_3_) δ 3.41 (dd, *J* = 13.2, 5.1 Hz, 1H), 3.67 (dd, *J* = 13.2, 3.8 Hz, 1H), 3.98 (q, *J* = 4.8 Hz, 1H), 5.07–5.17 (m, 2H), 5.19 (dd, *J* = 9.1, 5.1 Hz, 1H), 5.73 (d, *J* = 9.0 Hz, 1H), 6.25 (s, 1H), 7.32–7.42 (m, 5H); ^13^C NMR (101 MHz, CDCl_3_) δ 51.2, 53.4, 60.3, 67.7, 128.4, 128.6, 128.8, 135.9, 155.9, 167.3; HRMS (ESI-) *m*/*z* calcd for C_12_H_12_N_3_O_5_ [M − H]^−^: 274.0946; found: 274.0947.

#### 3.6.9. Synthesis of Benzyl (2-(Iodomethyl)-4-oxoazetidin-3-yl)carbamate (**9**)

Compound **7** (1.77 g, 5.38 mmol, 1.0 eq.) was dissolved in acetone (20 mL), and sodium iodide (4.04 g, 26.9 mmol, 5.0 eq.) was added. The reaction mixture was refluxed for 16 h. After completion, acetone was removed under reduced pressure, the residue was taken up in DCM (20 mL) and washed with water. The organic phase was dried over Na_2_SO_4_, filtered, and concentrated under reduced pressure. The crude product was purified by column chromatography on silica gel (DCM/acetone = 10:1) to afford the product as a white solid (1.24 g, 64% from **6**). ^1^H NMR (400 MHz, Acetone-*d*_6_) δ 3.33 (dd, *J* = 10.0, 7.0 Hz, 1H), 3.41 (dd, *J* = 10.0, 7.5 Hz, 1H), 4.15–4.21 (m, 1H), 5.09 (ddd, *J* = 9.7, 5.0, 1.5 Hz, 1H), 5.12 (d, *J* = 1.5 Hz, 2H), 7.18 (d, *J* = 9.8 Hz, 1H), 7.25–7.43 (m, 5H), 7.71 (s, 1H); ^13^C NMR (101 MHz, Acetone-*d*_6_) δ 4.9, 56.4, 61.8, 67.3, 128.8, 128.9, 129.3, 138.0, 156.9, 167.3; LC-MS: *m*/*z* (M + H)^+^ calculated 360.0, found 360.4.

#### 3.6.10. Synthesis of *N*-(2,4-Dimethoxybenzyl)-1-(2,2-dimethyl-1,3-dioxolan-4-yl)methanimine (**10**)

2,2-Dimethyl-1,3-dioxolane-4-carbaldehyde (25 g, 192 mmol, 1.0 eq.) was dissolved in dichloromethane (DCM, 500 mL), and MgSO_4_ (100 g) was added. The reaction mixture was cooled to 0 °C, and (2,4-dimethoxyphenyl)methanamine (29.15 mL, 194 mmol, 1.01 eq.) was added dropwise. The reaction was stirred at room temperature for 2 h under an argon atmosphere. The mixture was filtered through Celite and washed with DCM. The filtrate was concentrated under reduced pressure, and the crude product as an orange viscous fluid was assumed to be formed quantitatively and used directly in the next synthetic step. ^1^H NMR (400 MHz, CDCl_3_) δ 1.39 (s, 3H), 1.44 (s, 3H), 3.78 (s, 6H), 3.92 (dd, *J* = 8.4, 6.2 Hz, 1H), 4.18 (dd, *J* = 8.4, 6.8 Hz, 1H), 4.55 (s, 2H), 4.58–4.67 (m, 1H), 6.44 (h, *J* = 2.5 Hz, 2H), 7.06–7.13 (m, 1H), 7.68 (dt, *J* = 5.0, 1.5 Hz, 1H).

#### 3.6.11. Synthesis of 2-(1-(3,4-Dimethoxybenzyl)-2-(2,2-dimethyl-1,3-dioxolan-4-yl)-4-oxoazetidin-3-yl)isoindoline-1,3-dione (**11**)

To a solution of compound **10** in DCM (300 mL), triethylamine (54.1 mL, 388 mmol, 2.02 eq.) was added, and the reaction mixture was cooled to 0 °C. In a separate vessel, **2a** (48.1 g, 215 mmol, 1.12 eq.) was dissolved in DCM (150 mL) and added dropwise to the reaction mixture at 0 °C. After completion of the addition, the reaction mixture was stirred at room temperature for 16 h under an argon atmosphere. The progress of the reaction was monitored by TLC, after which the mixture was concentrated under reduced pressure to approximately half of its original volume. The resulting precipitate was collected by filtration and washed with DCM. The combined filtrates were washed with 1 M HCl (1 × 100 mL), saturated aqueous NaHCO_3_ (1 × 100 mL), and brine (1 × 100 mL). The organic phase was concentrated under reduced pressure, and the product was recrystallized from ethyl acetate. The resulting white solid was dried on air at 40 °C. ^1^H NMR (400 MHz, CDCl_3_) δ 1.19 (s, 3H), 1.25 (s, 3H), 3.58–3.66 (m, 2H), 3.75 (s, 3H), 3.79 (s, 3H), 4.19 (d, *J* = 14.5 Hz, 1H), 4.25–4.38 (m, 2H), 4.86 (d, *J* = 14.5 Hz, 1H), 5.19 (d, *J* = 5.4 Hz, 1H), 6.41 (d, *J* = 8.0 Hz, 2H), 7.20 (d, *J* = 8.0 Hz, 1H), 7.72 (dd, *J* = 5.5, 3.1 Hz, 2H), 7.81 (dd, *J* = 5.5, 3.0 Hz, 2H); LC-MS: *m*/*z* (M + H)^+^ calculated 467.2, found 467.7.

#### 3.6.12. Synthesis of Benzyl (1-(2,4-Dimethoxybenzyl)-2-(2,2-dimethyl-1,3-dioxolan-4-yl)-4-oxoazetidin-3-yl)carbamate (**13**)

Crude compound **11** (89.4 g, 0.192 mol, 1.0 eq.) was dissolved in ethanol (EtOH, 1.36 L), and hydrazine hydrate (32.6 mL, 0.672 mol, 3.5 eq.) was added. The reaction mixture was refluxed at 80 °C for 3 h, then cooled to room temperature. The resulting solids were collected by filtration and washed with EtOH. The filtrate was concentrated under reduced pressure, and the residue was taken up in EA (650 mL). Any precipitated solids were removed by filtration, and the organic phase was washed with water (2 × 150 mL). The resulting EA solution of 3-amino-1-(3,4-dimethoxybenzyl)-4-(2,2-dimethyl-1,3-dioxolan-4-yl)azetidin-2-one **12** was used directly in the next step without further purification; LC-MS: *m*/*z* (M + H)^+^ calculated 337.2, found 337.5.

To a solution of compound **12** in EA (from the previous step), saturated aqueous NaHCO_3_ (345 mL) was added, and the mixture was cooled to 0 °C. Benzyl chloroformate (33.8 mL, 0.24 mol, 1.25 eq.) was then added dropwise. The reaction mixture was stirred at room temperature for 2 h. The phases were separated, and the aqueous phase was extracted with EA (2 × 50 mL). The combined organic layers were washed with water (2 × 200 mL) and brine (200 mL), dried over Na_2_SO_4_, filtered, and concentrated under reduced pressure. The crude product was purified by crystallization from methanol (MeOH, 400 mL), affording a white solid, which was washed with MeOH (2 × 50 mL). The filtrate was concentrated, and the crystallization was repeated from MeOH (340 mL). The collected solids were obtained as the final product (18.72 g, 22% from start). ^1^H NMR (400 MHz, CDCl_3_) δ 1.32 (s, 3H), 1.44 (s, 3H), 3.53 (dd, *J* = 5.3, 3.6 Hz, 1H), 3.78–3.80 (m, 6H), 3.84 (dd, *J* = 8.9, 4.4 Hz, 1H), 3.97–4.02 (m, 1H), 4.04 (d, *J* = 14.6 Hz, 1H), 4.10–4.15 (m, 1H), 4.80 (d, *J* = 14.6 Hz, 1H), 5.04 (dd, *J* = 9.9, 5.3 Hz, 1H), 5.09 (d, *J* = 1.7 Hz, 2H), 5.85 (d, *J* = 9.9 Hz, 1H), 6.41–6.45 (m, 2H), 7.11 (d, *J* = 8.0 Hz, 1H), 7.30–7.38 (m, 5H); ^13^C NMR (101 MHz, CDCl_3_) δ 25.1, 26.3, 40.7, 55.5, 58.7, 59.0, 66.1, 67.4, 74.4, 98.6, 104.2, 110.4, 115.6, 127.0, 128.3, 128.4, 128.6, 131.4, 136.2, 155.9, 158.7, 161.1, 167.3; LC-MS: *m*/*z* (M + H)^+^ calculated 471.2, found 471.7.

#### 3.6.13. Synthesis of Benzyl (2-(1,2-Dihydroxyethyl)-1-(2,4-dimethoxybenzyl)-4-oxoazetidin-3-yl)carbamate (**14**)

Compound **13** (18.72 g, 39.8 mmol, 1.0 eq.) was dissolved in THF/H_2_O (1:4, 268 mL), and *p*-toluenesulfonic acid (*p*TsOH, 3.79 g, 19.9 mmol, 0.5 eq.) was added. The reaction mixture was heated to 70 °C and stirred for 16 h. After completion, the reaction mixture was neutralized to pH 7 using saturated aqueous NaHCO_3_. THF was removed under reduced pressure, and the resulting solid was collected by filtration and washed with water to afford an off-white solid (15.49 g, 91%). ^1^H NMR (400 MHz, DMSO) δ 3.18–3.32 (m, 2H), 3.54–3.62 (m, 1H), 3.64–3.70 (m, 1H), 3.75 (s, 3H), 3.76 (s, 3H), 4.16 (d, *J* = 15.5 Hz, 1H), 4.47 (d, *J* = 15.5 Hz, 1H), 4.58 (s, 1H), 4.87 (dd, *J* = 9.8, 5.2 Hz, 1H), 4.94 (s, 1H), 5.00–5.13 (m, 2H), 6.46 (dd, *J* = 8.3, 2.4 Hz, 1H), 6.54 (d, *J* = 2.4 Hz, 1H), 7.11 (d, *J* = 8.3 Hz, 1H), 7.32–7.39 (m, 5H), 7.75 (d, *J* = 9.8 Hz, 1H); ^13^C NMR (101 MHz, DMSO-*d*_6_) δ 55.2, 55.4, 57.5, 57.9, 63.1, 65.8, 71.0, 98.3, 104.4, 116.1, 127.7, 127.9, 128.4, 129.6, 136.8, 155.7, 157.7, 159.9, 166.8; LC-MS: *m*/*z* (M + H)^+^ calculated 431.2, found 431.7.

#### 3.6.14. Synthesis of Benzyl (1-(2,4-Dimethoxybenzyl)-2-formyl-4-oxoazetidin-3-yl)carbamate (**15**)

Compound **14** (15.49 g, 36.0 mmol, 1.0 eq.) was dissolved in a mixture of EA/H_2_O (4:1, 401 mL), and sodium periodate (8.56 g, 40.0 mmol, 1.11 eq.) was added. The reaction mixture was heated to 50 °C and stirred for 2 h, with progress monitored by TLC. After completion, the reaction mixture was cooled to room temperature and the phases were separated. The aqueous phase was extracted with EA (50 mL), and the combined organic layers were washed with water (150 mL) and brine (150 mL), dried over Na_2_SO_4_, filtered, and concentrated under reduced pressure to afford a light orange solid (13.89 g, 97%). ^1^H NMR (400 MHz, CDCl_3_) δ 3.75 (s, 3H), 3.79 (s, 3H), 4.15 (dd, *J* = 5.6, 2.1 Hz, 1H), 4.34 (d, *J* = 14.3 Hz, 1H), 4.55 (d, *J* = 14.1 Hz, 1H), 4.93 (dd, *J* = 7.6, 5.5 Hz, 1H), 5.03–5.10 (m, 2H), 5.55 (d, *J* = 7.6 Hz, 1H), 6.40–6.48 (m, 2H), 7.13 (d, *J* = 7.9 Hz, 1H), 7.25–7.38 (m, 5H), 9.52 (d, *J* = 2.1 Hz, 1H); ^13^C NMR (101 MHz, CDCl_3_) δ 41.0, 55.4, 55.6, 61.1, 64.0, 67.7, 98.7, 104.7, 115.0, 128.2, 128.5, 128.7, 131.8, 135.7, 155.6, 158.6, 161.5, 164.9, 198.3; LC-MS: *m*/*z* (M + H)^+^ calculated 399.2, found 399.5.

#### 3.6.15. Synthesis of Benzyl (1-(2,4-Dimethoxybenzyl)-2-(hydroxymethyl)-4-oxoazetidin-3-yl)carbamate (**16**)

Compound **15** (13.89 g, 34.9 mmol, 1.0 eq.) was dissolved in DCM/MeOH (4:1, 145 mL) and cooled to 0 °C in an ice bath. Sodium borohydride (2.64 g, 69.8 mmol, 2.0 eq.) was added portionwise. The reaction mixture was stirred at 0 °C for 2 h, after which cold water (100 mL) was added to quench the reaction, and the mixture was stirred for an additional 30 min. The phases were separated, and the aqueous layer was extracted with DCM (3 × 100 mL). The combined organic layers were washed with water (200 mL) and brine (200 mL), dried over Na_2_SO_4_, filtered, and concentrated under reduced pressure to afford the product as a light-yellow solid (13.46 g, 96%). ^1^H NMR (400 MHz, Acetone-*d*_6_) δ 3.68–3.75 (m, 2H), 3.79 (s, 3H), 3.84 (s, 3H), 3.93–4.01 (m, 1H), 4.12 (d, *J* = 14.9 Hz, 1H), 4.20–4.24 (m, 1H), 4.49 (d, *J* = 14.9 Hz, 1H), 5.04 (dd, *J* = 10.1, 5.2 Hz, 1H), 5.09 (s, 2H), 6.49 (dd, *J* = 8.3, 2.4 Hz, 1H), 6.56 (d, *J* = 2.4 Hz, 1H), 6.63 (d, *J* = 10.2 Hz, 1H), 7.17 (d, *J* = 8.3 Hz, 1H), 7.29–7.40 (m, 5H); ^13^C NMR (101 MHz, Acetone-*d*_6_) δ 38.7, 54.8, 55.0, 57.7, 58.3, 59.2, 66.2, 98.3, 104.5, 116.4, 127.9, 127.9, 128.4, 130.5, 137.1, 155.7, 158.5, 160.9, 166.7.

#### 3.6.16. Synthesis of 3-Amino-4-(hydroxymethyl)azetidin-2-one (**17**)

Compound **6** (2.65 g, 10.6 mmol, 1.0 eq.) was dissolved in MeOH (35 mL), and acetic acid (1.21 mL, 21.2 mmol, 2.0 eq.) was added. Subsequently, 10 wt% Pd/C (150 mg) was added, and the reaction mixture was stirred under a hydrogen atmosphere (balloon) for 8 h, with progress monitored by TLC. Upon completion, the reaction mixture was filtered through Celite and concentrated under reduced pressure to afford a colorless oil, which was used in the next step without further purification. ^1^H NMR (400 MHz, CDCl_3_) δ 3.79 (td, *J* = 5.0, 3.2 Hz, 1H), 3.88 (dd, *J* = 12.2, 5.0 Hz, 1H), 3.95 (dd, *J* = 12.3, 3.2 Hz, 1H), 4.39 (dd, *J* = 5.0, 1.2 Hz, 1H), 5.90 (s, 1H).

#### 3.6.17. Synthesis of (*Z*)-2-(((1-(*tert*-Butoxy)-2-methyl-1-oxopropan-2-yl)oxy)imino)-2-(2-((*tert*-butoxycarbonyl)amino)thiazol-4-yl)acetic Acid (**18a**)

Compound **18a** was prepared according to a previously reported procedure [40] from (*Z*)-2-(2-aminothiazol-4-yl)-2-(((1-(*tert*-butoxy)-2-methyl-1-oxopropan-2-yl)oxy)imino)acetic acid (1.65 g, 5.0 mmol, 1.0 eq.), affording the product as a white powder (732 mg, 44%). ^1^H NMR (400 MHz, DMSO-*d*_6_) δ 1.38 (s, 9H), 1.42 (s, 6H), 1.47 (s, 9H), 7.34–7.39 (m, 1H), 11.79 (s, 1H), 13.83 (s, 1H).

#### 3.6.18. Synthesis of *tert*-Butyl (*Z*)-2-(((1-(2-((*tert*-Butoxycarbonyl)amino)thiazol-4-yl)-2-((2-(hydroxymethyl)-4-oxoazetidin-3-yl)amino)-2-oxoethylidene)amino)oxy)-2-methylpropanoate (**18**)

To a suspension of **18a** (5.28 g, 12.3 mmol, 1.0 eq.) and 2,2′-dibenzothiazolyl disulfide (5.73 g, 17.2 mmol, 1.4 eq.) in DCM (100 mL) was added triphenylphosphine (TPP, 4.68 g, 17.8 mmol, 1.45 eq.), followed by dropwise addition of triethylamine (2.4 mL, 17.2 mmol, 1.4 eq.). The reaction mixture was stirred at room temperature for 16 h, then concentrated under reduced pressure to afford **18b**. LC-MS: *m*/*z* (M + H)^+^ calculated 579.1, found 578.8. To a solution of **18b** (483 mg, 0.835 mmol, 1.16 eq.) in DMF (10 mL) at 0 °C was added a solution of compound **17** (127 mg, 0.72 mmol, assumed quantitative from the previous step, 1.0 eq.) in DMF (10 mL), followed by DIPEA (374 μL, 2.16 mmol, 3.0 eq.). The reaction mixture was allowed to warm to room temperature and stirred for an additional 16 h. After completion, the reaction mixture was concentrated under reduced pressure and purified by silica gel column chromatography (EA/H = 1/4 → 4/1) to afford the crude title compound as a pale-yellow solid (114 mg, 30%). ^1^H NMR (400 MHz, CDCl_3_) δ 1.43 (s, 9H), 1.52 (s, 9H), 1.55 (s, 3H), 1.56 (s, 3H), 1.90 (s, 1H), 3.82–3.87 (m, 2H), 4.02 (q, *J* = 4.6 Hz, 1H), 4.26 (s, 1H), 5.43 (ddd, *J* = 7.7, 4.8, 1.1 Hz, 1H), 6.40 (s, 1H), 7.30 (s, 1H), 7.41–7.51 (m, 2H), 7.52–7.59 (m, 1H), 7.61–7.72 (m, 2H), 7.86 (d, *J* = 7.6 Hz, 1H), 8.67 (s, 1H); ^13^C NMR (101 MHz, CDCl_3_) δ 23.7, 24.3, 28.1, 28.3, 31.6, 36.6, 54.9, 58.1, 60.5, 82.3, 83.4, 113.4, 132.1, 148.9, 160.5, 162.7, 163.2, 167.0, 173.8; LC-MS: *m*/*z* (M − H)^–^ calculated 526.2, found 526.2.

#### 3.6.19. Synthesis of *tert*-Butyl (*Z*)-2-(((1-(2-((*tert*-Butoxycarbonyl)amino)thiazol-4-yl)-2-((2-(iodomethyl)-4-oxoazetidin-3-yl)amino)-2-oxoethylidene)amino)oxy)-2-methylpropanoate (**19**)

Compound **18** (114 mg, 0.216 mmol, 1.0 eq.), TPP (113 mg, 0.432 mmol, 2.0 eq.), and imidazole (29 mg, 0.432 mmol, 2.0 eq.) were dissolved in DCM (10 mL); next, iodine (110 mg, 0.432 mmol, 2.0 eq.) was added. The reaction mixture was stirred at room temperature for 16 h. The reaction mixture was then diluted with DCM (10 mL) and extracted with saturated aqueous sodium thiosulfate (10 mL), water (20 mL), and brine (20 mL). The organic phase was dried over Na_2_SO_4_, filtered, and concentrated under reduced pressure. The crude product was purified by silica gel column chromatography (H/EA = 3:1 → 1:3) to afford the product as a light-yellow solid (64 mg, 47%). ^1^H NMR (400 MHz, CDCl_3_) δ 1.42 (s, 9H), 1.50 (s, 9H), 1.54 (s, 3H), 1.55 (s, 3H), 3.23 (t, *J* = 10.3 Hz, 1H), 3.35 (dd, *J* = 10.3, 4.0 Hz, 1H), 4.24 (dt, *J* = 10.3, 4.4 Hz, 1H), 5.27 (ddd, *J* = 7.8, 4.8, 1.6 Hz, 1H), 6.76 (s, 1H), 7.30 (s, 1H), 8.44 (d, *J* = 7.8 Hz, 1H), 9.10 (s, 1H); ^13^C NMR (101 MHz, CDCl_3_) δ 5.6, 23.9, 24.2, 28.1, 28.2, 56.5, 58.7, 82.4, 83.3, 115.0, 141.7, 148.9, 160.4, 163.0, 166.0, 173.9; LC-MS: *m*/*z* (M − H)^–^ calculated 636.1, found 636.0.

#### 3.6.20. Synthesis of *tert*-Butyl (*Z*)-2-(((2-((2-(Azidomethyl)-4-oxoazetidin-3-yl)amino)-1-(2-((*tert*-butoxycarbonyl)amino)thiazol-4-yl)-2-oxoethylidene)amino)oxy)-2-methylpropanoate (**20**)

Compound **19** (62 mg, 0.097 mmol, 1.0 eq.) was dissolved in THF (7 mL) and cooled to 0 °C. Triethylamine (27.8 μL, 0.195 mmol, 2.0 eq.) and tetrabutylammonium azide (37 mg, 0.127 mmol, 1.3 eq.) were added, and the reaction mixture was stirred for 16 h. The reaction mixture was poured onto an ice–water mixture (20 mL) and extracted with EA (3 × 20 mL). The combined organic layers were washed with brine, dried over Na_2_SO_4_, filtered, and concentrated under reduced pressure. The crude product was purified by silica gel column chromatography (EA/H = 1:2 → 2:1) to afford the title compound as an off-white solid (29 mg, 54%). ^1^H NMR (400 MHz, CDCl_3_) δ 1.44 (s, 9H), 1.52 (s, 9H), 1.57 (s, 6H), 3.51 (dd, *J* = 13.0, 7.2 Hz, 1H), 3.75 (dd, *J* = 13.0, 3.7 Hz, 1H), 4.03–4.12 (m, 1H), 5.44 (ddd, *J* = 8.3, 5.1, 1.3 Hz, 1H), 6.63–6.68 (m, 1H), 7.29 (s, 1H), 8.19 (d, *J* = 8.2 Hz, 1H), 8.87 (s, 1H); ^13^C NMR (101 MHz, CDCl_3_) δ 23.8, 24.2, 28.1, 28.1, 28.3, 52.0, 53.4, 58.4, 82.4, 83.4, 114.7, 141.8, 148.8, 152.4, 160.3, 163.0, 166.7, 173.8; LC-MS: *m*/*z* (M − H)^–^ calculated 551.2, found 551.3.

#### 3.6.21. Synthesis of (*Z*)-2-(Azidomethyl)-3-(2-(((1-(*tert*-butoxy)-2-methyl-1-oxopropan-2-yl)oxy)imino)-2-(2-((*tert*-butoxycarbonyl)amino)thiazol-4-yl)acetamido)-4-oxoazetidine-1-sulfonic Acid (**21**)

Compound **20** (29 mg, 0.0524 mmol, 1.0 eq.) was dissolved in DMF (2 mL), and DMF·SO_3_ complex (80 mg, 0.524 mmol, 10 eq.) was added. The reaction mixture was stirred at room temperature for 4 h, with progress monitored by LC-MS. After completion, EA (20 mL) was added, and the mixture was washed with water (10 mL) and brine until the aqueous phase reached approximately neutral pH (~7). The organic phase was dried over Na_2_SO_4_, filtered, and concentrated under reduced pressure to afford the product as a colorless viscous liquid (22 mg, 66%). ^1^H NMR (400 MHz, MeOD) δ 1.49 (s, 9H), 1.55–1.57 (m, 15H), 3.77 (dd, *J* = 13.4, 3.3 Hz, 1H), 3.94 (dd, *J* = 13.4, 5.2 Hz, 1H), 4.33 (td, *J* = 5.4, 3.3 Hz, 1H), 5.44 (d, *J* = 5.7 Hz, 1H), 7.35 (s, 1H); ^13^C NMR (101 MHz, MeOD_4_) 24.2, 24.4, 28.2, 28.4, 50.2, 57.7, 59.0, 83.0, 83.1, 84.3, 114.7, 143.2, 150.0, 154.5, 162.1, 164.6, 165.2, 174.8; LC-MS: *m*/*z* (M − H)^–^ calculated 631.2, found 631.2.

#### 3.6.22. Synthesis of *tert*-Butyl (1-(2,4-Dimethoxybenzyl)-2-(2,2-dimethyl-1,3-dioxolan-4-yl)-4-oxoazetidin-3-yl)carbamate (**22**)

Compound **12** (93.02 g, 276.0 mmol, 1.0 eq.) was dissolved in THF (150 mL), and di-*tert*-butyl dicarbonate (66.41 g, 304.0 mmol, 1.1 eq.) was added. The reaction mixture was stirred at room temperature for 16 h under an argon atmosphere. After completion, the resulting precipitate was removed by filtration, and the filtrate was concentrated under reduced pressure. The residue was dissolved in DCM (200 mL) and washed with water (100 mL), 10% aqueous citric acid (100 mL), and brine (100 mL). The organic phase was concentrated under reduced pressure. Due to the hygroscopic nature of the product, complete drying was not achieved and crude material was used directly in the next step without further purification. ^1^H NMR (400 MHz, CDCl_3_) δ 1.43 (s, 6H), 1.45–1.48 (m, 9H), 3.47–3.55 (m, 2H), 3.77–3.83 (m, 6H), 3.98–4.08 (m, 1H), 4.07–4.15 (m, 1H), 4.80 (d, *J* = 14.5 Hz, 1H), 4.91–5.03 (m, 1H), 5.29–5.36 (m, 1H), 6.39–6.48 (m, 2H), 7.12 (t, *J* = 8.7 Hz, 1H); LC-MS: *m*/*z* (M + H)^+^ calculated 437.2, found 436.7.

#### 3.6.23. Synthesis of *tert*-Butyl (1-(2,4-Dimethoxybenzyl)-2-(hydroxymethyl)-4-oxoazetidin-3-yl)carbamate (**25**)

Compound **22** (54.45 g, 124.7 mmol, 1.0 eq.) was dissolved in THF (500 mL), and a solution of *p*TsOH (9.5 g, 49.9 mmol, 0.4 eq.) in water (125 mL) was added. The reaction mixture was heated to 70 °C and stirred for 16 h under an argon atmosphere. LC–MS analysis indicated partial cleavage of the Boc protecting group under these conditions. Therefore, Boc_2_O (27.2 g, 124.7 mmol, 1.0 eq.) and DIPEA (43.4 mL, 249.6 mmol, 2.0 eq.) were added, and the reaction mixture was stirred at room temperature for an additional 3 h under argon. The solvent was removed under reduced pressure, and the residue was extracted with EA (5 × 50 mL). The combined organic layers were washed with saturated aqueous NaHCO_3_ (100 mL), dried over Na_2_SO_4_, filtered, and concentrated under reduced pressure. This afforded the title product **23** as an orange viscous liquid (35.9 g, 73.0% crude); LC-MS: *m*/*z* (M + H)^+^ calculated 397.2, found 397.3. Compound **25** (35.9 g, 90.6 mmol, 1.0 eq.) was dissolved in EA (400 mL), and a solution of sodium periodate (19.4 g, 90.6 mmol, 1.0 eq.) in water (100 mL) was added. The reaction mixture was heated to 50 °C and stirred for 3 h under an argon atmosphere. The phases were separated, and the aqueous phase was extracted with EA (3 × 100 mL). The combined organic layers were washed with water (100 mL) and brine (100 mL), dried over Na_2_SO_4_, filtered, and concentrated under reduced pressure. This afforded the product **24** as an orange viscous liquid (28.05 g, 85% crude).

Compound **24** (28.1 g, 77.0 mmol, 1.0 eq.) was dissolved in DCM/MeOH (4:1, 100 mL) and cooled to 0 °C. Sodium borohydride (5.82 g, 154.0 mmol, 2.0 eq.) was added portion wise, and the reaction mixture was stirred at room temperature for 16 h under an argon atmosphere. Water (20 mL) was added, and the mixture was stirred for an additional 30 min. The phases were separated, and the aqueous phase was extracted with DCM (3 × 80 mL). The combined organic layers were washed with water (50 mL) and brine (50 mL), dried over Na_2_SO_4_, filtered, and concentrated under reduced pressure. This afforded the crude title product as a yellow oil (28.2 g, quant., crude), which was used directly in the next step without further purification. ^1^H NMR (400 MHz, Acetone) δ 1.41 (s, 9H), 3.67–3.72 (m, 3H), 3.79 (s, 3H), 3.84 (s, 3H), 3.91–4.04 (m, 1H), 4.10 (d, *J* = 14.9 Hz, 1H), 4.16–4.26 (m, 1H), 4.48 (d, *J* = 14.9 Hz, 1H), 4.97 (dd, *J* = 10.1, 5.1 Hz, 1H), 6.24 (d, *J* = 10.1 Hz, 1H), 6.49 (dd, *J* = 8.3, 2.4 Hz, 1H), 6.56 (d, *J* = 2.4 Hz, 1H), 7.17 (d, *J* = 8.3 Hz, 1H); LC-MS: *m*/*z* (M + H)^+^ calculated 367.2, found 366.8.

#### 3.6.24. Synthesis of *tert*-Butyl (2-(Hydroxymethyl)-4-oxoazetidin-3-yl)carbamate (**26**)

Compound **25** (14.01 g, 38.2 mmol, 1.0 eq.) was dissolved in a mixture of MeCN/H_2_O (2:1, 574 mL), and K_2_S_2_O_8_ (14.47 g, 53.5 mmol, 1.4 eq.) and K_2_HPO_4_ (8.66 g, 49.7 mmol, 1.3 eq.) were added. The reaction mixture was heated to 95 °C and stirred for 3 h under an argon atmosphere. After completion, MeCN was removed under reduced pressure, and the residue was extracted with EA (5 × 50 mL). The combined organic layers were concentrated under reduced pressure, and the crude product was purified by column chromatography on silica gel (DCM/acetone = 10:1) to afford the product as an orange powder (2.91 g, 17%). ^1^H NMR (400 MHz, MeOD_4_) δ 1.44 (s, 9H), 3.63 (dd, *J* = 11.8, 5.5 Hz, 1H), 3.67–3.75 (m, 1H), 3.76–3.80 (m, 1H), 3.81–3.85 (m, 1H), 4.98 (d, *J* = 5.0 Hz, 1H).

#### 3.6.25. Synthesis of 2-(Prop-2-yn-1-ylthio)-4-(pyridin-4-yl)thiazole (**27a**)

4-(Pyridin-4-yl)thiazole-2-thiol (583 mg, 3.0 mmol, 1.0 eq.) was dissolved in MeCN (20 mL), and K_2_CO_3_ (829 mg, 6.0 mmol, 2.0 eq.) was added. After several minutes, 3-bromoprop-1-yne (271 μL, 3.6 mmol, 1.2 eq.) was added, and the reaction mixture was stirred at 80 °C under an argon atmosphere, with progress monitored by TLC. After completion, the solvent was removed under reduced pressure, and the residue was dissolved in EA (20 mL). The mixture was washed with water (1 × 20 mL) and 1.0 M aqueous NaOH (1 × 20 mL). The organic phase was dried over Na_2_SO_4_, filtered, and concentrated under reduced pressure. This afforded the title product as a brown solid (0.52 g, 74%). 1H NMR (400 MHz, CDCl_3_) δ 2.30 (t, *J* = 2.6 Hz, 1H), 4.06 (d, *J* = 2.6 Hz, 2H), 7.64 (s, 1H), 7.73–7.81 (m, 2H), 8.66–8.72 (m, 2H).

#### 3.6.26. Synthesis of (*Z*)-3-(2-(((1-(*tert*-Butoxy)-2-methyl-1-oxopropan-2-yl)oxy)imino)-2-(2-((*tert*-butoxycarbonyl)amino)thiazol-4-yl)acetamido)-2-oxo-4-((4-(((4-(pyridin-4-yl)thiazol-2-yl)thio)methyl)-1*H*-1,2,3-triazol-1-yl)methyl)azetidine-1-sulfonic Acid (**27**)

Compound **21** (54 mg, 0.0854 mmol, 1.0 eq.) was dissolved in MeCN (10 mL), and compound **27a** (24 mg, 0.102 mmol, 1.2 eq.) was added. Aqueous sodium ascorbate (0.25 M, 1.0 mL) and aqueous CuSO_4_ (0.25 M, 0.5 mL) were then added, and the reaction mixture was stirred at room temperature for 16 h. After completion, water was added, and the resulting solids were collected by filtration, washed with EA and DCM, and dried to afford the crude product, which was used in the next step without further purification. ^1^H NMR (400 MHz, Acetone-*d*_6_) δ 1.44 (s, 15H), 1.45 (s, 9H), 3.93–3.98 (m, 1H), 4.22–4.32 (m, 1H), 4.44 (dd, *J* = 9.1, 4.0 Hz, 1H), 4.62–4.71 (m, 1H), 4.73 (s, 1H), 5.42 (t, *J* = 7.0 Hz, 1H), 7.38 (s, 1H), 7.81–7.86 (m, 1H), 7.96–8.04 (m, 1H), 8.14 (s, 1H), 8.21–8.31 (m, 1H), 8.51 (s, 1H), 8.52–8.60 (m, 1H), 8.68 (d, *J* = 8.4 Hz, 1H); LC-MS: *m*/*z* (M − H)^–^ calculated 863.2, found 863.5.

#### 3.6.27. Synthesis of (*Z*)-2-(((1-(2-Aminothiazol-4-yl)-2-oxo-2-((2-oxo-4-((4-(((4-(pyridin-4-yl)thiazol-2-yl)thio)methyl)-1*H*-1,2,3-triazol-1-yl)methyl)-1-sulfoazetidin-3-yl)amino)ethylidene)amino)oxy)-2-methylpropanoic Acid (**28**)

Crude compound **27** from the previous step was dissolved in DCM (3 mL), and trifluoroacetic acid (TFA, 1 mL) was added. The reaction mixture was stirred at room temperature for 4 h. The solvents were then removed under reduced pressure, and the residue was dissolved in 10% TFA in MeOH and purified by reversed-phase flash chromatography on an Isolera One system (Biotage) using a SNAP KP-C18-HS column (30 g) and a mobile phase of 0.1% TFA in deionized water/MeCN with the following gradient: 0% MeCN for 1 column volume, 0–100% MeCN over 6 column volumes, followed by 100% MeOH for 2 column volumes. This afforded the title product as a pale green solid (9.8 mg, 16% from compound **21**). ^1^H NMR (400 MHz, MeOD_4_) δ 1.57 (s, 6H), 4.17 (d, *J* = 2.7 Hz, 1H), 4.41–4.48 (m, 1H), 4.59–4.85 (m, 3H), 5.37 (d, *J* = 5.4 Hz, 1H), 7.24 (s, 1H), 8.26 (s, 1H), 8.58 (d, *J* = 6.3 Hz, 2H), 8.83 (s, 2H). Purity (t_r_ = 3.793 min; λ = 254 nm) = 91.6%; HRMS (ESI^+^) *m*/*z* calcd for C_24_H_25_N_10_O_8_S_4_ [M + H]^+^: 709.07342; found: 709.07355.

#### 3.6.28. Synthesis of 1-Methyl-4-(2-(prop-2-yn-1-ylthio)thiazol-4-yl)pyridin-1-ium (**29a**)

Compound **27a** (131 mg, 0.56 mmol, 1.0 eq.) was dissolved in MeCN (5 mL) and transferred to a pressure tube. Methyl iodide (106 μL, 1.69 mmol, 3.0 eq.) was added, and the reaction mixture was stirred at 80 °C for 16 h. After completion, the reaction mixture was cooled to room temperature, and diethyl ether (10 mL) was added. The resulting precipitate was collected by filtration and washed with ether to afford the product as a light-yellow solid (128 mg, 92.6%). ^1^H NMR (400 MHz, DMSO-*d*_6_) δ 3.36 (t, *J* = 2.6 Hz, 1H), 4.26 (d, *J* = 2.6 Hz, 2H), 4.30 (s, 3H), 8.52–8.58 (m, 2H), 8.95 (s, 1H), 8.97–9.04 (m, 2H).

#### 3.6.29. Synthesis of (*Z*)-4-(2-(((1-((3-(2-(2-Aminothiazol-4-yl)-2-(((2-carboxypropan-2-yl)oxy)imino)acetamido)-4-oxo-1-sulfoazetidin-2-yl)methyl)-1*H*-1,2,3-triazol-4-yl)methyl)thio)thiazol-4-yl)-1-methylpyridin-1-ium (**30**)

Compound **21** (54 mg, 0.0854 mmol, 1.0 eq.) was dissolved in MeCN (10 mL), and compound **29a** (21 mg, 0.0854 mmol, 1.0 eq.) was added. Aqueous sodium ascorbate (0.25 M, 1.0 mL) and aqueous CuSO_4_ (0.25 M, 0.5 mL) were then added, and the reaction mixture was stirred at room temperature for 16 h. After completion, water was added, and the resulting solids were collected by filtration, washed with EA and DCM, and dried to afford the crude product, which was used in the next step without further purification. LC-MS: *m*/*z* (M)^+^ calculated 879.2, found 879.4.

Crude compound **29** from the previous step was dissolved in DCM (3 mL), and trifluoroacetic acid (TFA, 1 mL) was added. The reaction mixture was stirred at room temperature for 4 h. The solvents were then removed under reduced pressure, and the residue was dissolved in 10% TFA in MeOH and purified by reversed-phase flash chromatography on an Isolera One system (Biotage) using a SNAP KP-C18-HS column (30 g) and a mobile phase of 0.1% TFA in deionized water/MeCN with the following gradient: 0% MeCN for 1 column volume, 0–100% MeCN over 6 column volumes, followed by 100% MeOH for 2 column volumes. This afforded the title product as a pale green solid (12 mg, 19% from compound **23**). ^1^H NMR (400 MHz, DMSO) δ 1.21–1.28 (m, 6H), 4.21 (q, *J* = 5.3 Hz, 1H), 4.29 (s, 3H), 4.65 (s, 2H), 4.69 (t, *J* = 7.5 Hz, 1H), 4.81 (dd, *J* = 14.8, 4.1 Hz, 1H), 5.24 (dd, *J* = 8.8, 5.4 Hz, 1H), 6.64 (s, 1H), 7.32 (s, 2H), 8.15 (s, 1H), 8.53–8.57 (m, 2H), 8.87 (s, 1H), 8.92–8.96 (m, 2H); Purity (t_r_ = 3.843 min; λ = 254 nm) = 91.2%; HRMS (ESI^+^) *m*/*z* calcd for C_25_H_27_N_10_O_8_S_4_ [M + H]^+^: 723.08907; found: 723.08588.

#### 3.6.30. Synthesis of *tert*-Butyl (*Z*)-2-(((1-(2-((*tert*-Butoxycarbonyl)amino)thiazol-4-yl)-2-oxo-2-((2-oxo-4-(((4-(pyridin-4-yl)thiazol-2-yl)thio)methyl)azetidin-3-yl)amino)ethylidene)amino)oxy)-2-methylpropanoate (**31**)

4-(Pyridin-4-yl)thiazole-2-thiol (37 mg, 0.188 mmol, 1.5 eq.) was dissolved in MeCN (5 mL), and K_2_CO_3_ (35 mg, 0.250 mmol, 2.0 eq.) was added. The reaction mixture was stirred at room temperature for 30 min, after which compound **19** (80 mg, 0.125 mmol, 1.0 eq.) was added. The reaction mixture was heated to 80 °C under an argon atmosphere and stirred for 16 h. After completion, the solvent was removed under reduced pressure, and the crude was taken up in EA (20 mL) and washed with water (20 mL). The phases were separated, and the aqueous layer was extracted with EA (20 mL). The combined organic layers were washed with brine (20 mL), dried over Na_2_SO_4_, filtered, and concentrated under reduced pressure. The crude product was purified by reversed-phase flash chromatography on an Isolera One system (Biotage) using a SNAP KP-C18-HS column (30 g) and a mobile phase of 0.1% TFA in deionized water/MeCN with the following gradient: 0% MeCN for 1 column volume, 0–100% MeCN over 6 column volumes, followed by 100% MeOH for 2 column volumes. This afforded the title product as a light-brown solid (28 mg, 32%). ^1^H NMR (400 MHz, CDCl_3_) δ 1.41 (s, 9H), 1.50 (s, 9H), 1.56 (s, 6H), 3.47 (dd, *J* = 14.4, 7.6 Hz, 1H), 3.72 (dd, *J* = 14.4, 4.9 Hz, 1H), 4.37 (dt, *J* = 7.6, 4.9 Hz, 1H), 5.41 (ddd, *J* = 7.6, 4.9, 1.3 Hz, 1H), 6.95 (s, 1H), 7.30 (s, 1H), 7.58 (s, 1H), 7.67–7.74 (m, 2H), 8.31 (d, *J* = 7.6 Hz, 1H), 8.61–8.70 (m, 2H), 9.62 (s, 1H); ^13^C NMR (101 MHz, CDCl_3_) δ 23.8, 24.2, 28.1, 28.3, 35.1, 54.1, 59.4, 82.3, 83.4, 114.5, 116.8, 120.5, 140.7, 141.9, 149.1, 150.4, 152.8, 160.3, 163.2, 164.6, 166.5, 173.9; LC-MS: *m*/*z* (M − H)^–^ calculated 702.2, found 702.3.

#### 3.6.31. Synthesis of (*Z*)-3-(2-(((1-(*tert*-Butoxy)-2-methyl-1-oxopropan-2-yl)oxy)imino)-2-(2-((*tert*-butoxycarbonyl)amino)thiazol-4-yl)acetamido)-2-oxo-4-(((4-(pyridin-4-yl)thiazol-2-yl)thio)methyl)azetidine-1-sulfonic Acid (**32**)

Compound **31** (25 mg, 0.0356 mmol, 1.0 eq.) was dissolved in DMF (2 mL), and DMF·SO_3_ complex (55 mg, 0.356 mmol, 10 eq.) was added. The reaction mixture was stirred at room temperature for 4 h, with progress monitored by LC-MS. After completion, EA (20 mL) was added, and the mixture was washed with water (10 mL) and brine until the aqueous phase reached approximately neutral pH (~7). The organic phase was dried over Na_2_SO_4_, filtered, and concentrated under reduced pressure to afford the product as a white solid (28 mg, quantitative yield). ^1^H NMR (400 MHz, MeOD) δ 1.47 (s, 9H), 1.53 (s, 15H), 3.60 (dd, *J* = 14.2, 9.0 Hz, 1H), 4.20 (dd, *J* = 14.2, 4.4 Hz, 1H), 4.67 (ddd, *J* = 9.0, 5.6, 4.4 Hz, 1H), 5.38 (d, *J* = 5.6 Hz, 1H), 7.34 (s, 1H), 8.03–8.07 (m, 2H), 8.10 (s, 1H), 8.53–8.58 (m, 2H); LC-MS: *m*/*z* (M − H)^–^ calculated 782.1, found 782.2.

#### 3.6.32. Synthesis of (*Z*)-2-(((1-(2-Aminothiazol-4-yl)-2-oxo-2-((2-oxo-4-(((4-(pyridin-4-yl)thiazol-2-yl)thio)methyl)-1-sulfoazetidin-3-yl)amino)ethylidene)amino)oxy)-2-methylpropanoic Acid (**33**)

Compound **32** (28 mg, 0.0356 mmol, 1.0 eq.) was dissolved in DCM (3 mL), then TFA (500 μL) was added and the reaction was stirred for 16 h at room temperature. The crude product was purified by reversed-phase flash chromatography on an Isolera One system (Biotage) using a SNAP KP-C18-HS column (30 g) and a mobile phase of 0.1% TFA in deionized water/MeCN with the following gradient: 0% MeCN for 1 column volume, 0–100% MeCN over 6 column volumes, followed by 100% MeOH for 2 column volumes. This afforded the title product as a light-brown solid (7.67 mg, 30.4%). ^1^H NMR (400 MHz, D_2_O) δ 1.43 (s, 6H), 3.47 (dd, *J* = 14.2, 7.6 Hz, 1H), 3.59 (dd, *J* = 14.2, 5.6 Hz, 1H), 4.30 (dt, *J* = 7.6, 5.6 Hz, 1H), 5.24 (d, *J* = 4.8 Hz, 1H), 7.08 (s, 1H), 8.30–8.37 (m, 2H), 8.38 (s, 1H), 8.62–8.65 (m, 2H); ^13^C NMR (101 MHz, DMSO-*d*_6_) 24.3, 24.3, 24.4, 24.4, 31.8, 43.3, 56.8, 57.0, 82.2, 82.4, 110.7, 115.1, 118.0, 122.4, 123.0, 124.4, 144.2, 149.7, 158.6, 158.9, 162.4, 166.7, 169.3, 175.0; Purity (t_r_ = 3.933 min; λ = 254 nm) = 100%; HRMS (ESI^+^) *m*/*z* calcd for C_21_H_22_N_7_O_8_S_4_ [M + H]^+^: 628.04072; found: 628.04089.

## 4. Conclusions

In conclusion, we developed two alternative synthetic routes toward the monocyclic β-lactam synthon **I**, which carries handles for late-stage functionalization at all three positions of the monobactam core (N1, C3 and C4), originating from ethyl glyoxylate and glyceraldehyde acetonide, respectively. The acetonide-based route proved more efficient overall, delivering the DMB-protected alcohols **16** and **25** in near-quantitative yields over the oxidative cleavage and reduction sequence and avoiding the low-yielding ester reduction required in the glyoxylate route, with subsequent DMB removal from the Cbz-protected **16** proceeding in 70% yield to give **6**. The C4 branch point provides access to distinct downstream pathways from a common late-stage intermediate, through mesylate activation to the azide **8** and the iodide **9**, and through Appel iodination of the ATMO-substituted alcohol to **19**, enabling either copper-catalyzed azide–alkyne cycloaddition or direct thioether alkylation. This was exploited to prepare three sulfonated monobactam derivatives (**28**, **30**, **33**) bearing a ceftaroline-derived side chain and differing in C4 linkage type and *N*-methylation status, without modification of the core synthetic sequence, although in low overall yields (0.10 to 0.29%).

The negative biological outcome is itself informative. The C4 position tolerates both triazole and thioether linkages and is compatible with the *cis* configuration, so the loss of activity is attributable to the appended group rather than to the point or mode of attachment. Across both published series, antibacterial activity at this position is associated with substituents that also promote uptake, siderophores in the thioether series and an iron-chelating pyridone in the triazole series [9,25]. The ceftaroline-derived side chain enhanced neither uptake nor measurable PBP affinity. Future C4 diversification of this scaffold should therefore prioritize substituents that contribute to uptake, to target affinity, or to both.

## Figures and Tables

**Table 1:** Optimization of DMB deprotection of Cbz-protected β-lactams **4** and **16** to β-lactam **5** or **6** respectively (Scheme 1 and Scheme 2). Reagent equivalents are relative to starting material.

Entry	Start Compound	Product	Reagent	Solvent	Ratio	Volume/mmol	T	t (h)	Yield (%) ^1^
1	**16**	**6**	4.3 eq. K_2_S_2_O_8_, 4.2 eq. K_2_HPO_4_	MeCN/H_2_O	2/1	12 mL	80 °C	16	0
2	**16**	**6**	1.4 eq. K_2_S_2_O_8_, 1.3 eq. K_2_HPO_4_	MeCN/H_2_O	2/1	12 mL	95 °C	4	0
3	**16**	**6**	3 eq. CAN ^2^	MeCN/H_2_O	1/1	8 mL	−10 °C	2	0
4	**16**	**6**	50 eq. TFA ^3^	DCM	/	7 mL	rt	18	0
5	**16**	**6**	4 eq. *p*TsOH	toluene	/	20 mL	110	2	0
6	**16**	**6**	1.4 eq. K_2_S_2_O_8_, 1.3 eq. K_2_HPO_4_^4^	MeCN/H_2_O	2/1	20 mL	95 °C	4	70
7	**4**	**5**	4.3 eq. K_2_S_2_O_8_, 4.2 eq. K_2_HPO_4_	MeCN/H_2_O	2/1	13 mL	95 °C	6	0
8	**4**	**5**	1.4 eq. K_2_S_2_O_8_, 1.3 eq. K_2_HPO_4_	MeCN/H_2_O	2/1	13 mL	95 °C	3	0
9	**4**	**5**	1.4 eq. K_2_S_2_O_8_, 1.3 eq. K_2_HPO_4_	MeCN/H_2_O	2/1	13 mL	95 °C	3	94
10	**4**	**5**	1.4 eq. K_2_S_2_O_8_, 1.3 eq. K_2_HPO_4_	MeCN/H_2_O	2/1	20 mL	95 °C	5	72

^1^ Isolated yield after purification. ^2^ Ceric ammonium nitrate. ^3^ Trifluoroacetic acid. ^4^ After 2 h, additional 0.3 eq. of both reagents were added.

**Table 2:** Optimization of NaBH_4_-mediated ester reduction of Cbz-protected β-lactam **5** to alcohol **6** (Scheme 1). Yields refer to isolated product after chromatographic purification.

Entry	Reductant	Solvent	Ratio	Volume/mmol	T	t (h)	Yield 7 (%)
1	8 eq. NaBH_4_	THF/H_2_O	1/1	5 mL	0 °C → rt	16	43
2	6 eq. NaBH_4_	THF/H_2_O	1/1	5 mL	0 °C → rt	16	47
3	4 eq. NaBH_4_	THF/H_2_O	1/1	5 mL	0 °C → rt	16	47
4	2 eq. NaBH_4_	THF/H_2_O	1/1	5 mL	0 °C → rt	16	52
5	8 eq. NaBH_4_	THF/H_2_O	1/1	10 mL	0 °C → rt	14	57
6	2 eq. NaBH_4_	MeOH	/	5 mL	0 °C → rt	13	34

**Table 3:** PBP1b inhibition and antibacterial activity results of selected monobactam derivatives. Residual activity and IC_50_ are recorded as mean ± standard deviation. Minimum inhibitory concentrations (MICs) were determined against: *S. aureus* ATCC 29213, *E. coli* ATCC 25922, *E. coli* N43 (CGSC no. 5583), and *E. coli* D22 (CGSC no. 5163).

Entry	Cpd	RA (%) at 100 μM/ IC_50_ (μM) at PBP1b ^1^	MIC *S. aureus* (μg/mL)	MIC *E. coli* (μg/mL)	MIC *E. coli* N43 (μg/mL)	MIC *E. coli* D22 (μg/mL)
1	**19**	100 ± 9%	>128	>128	>128	>128
2	**21**	83 ± 11%	>128	>128	>128	>128
3	**27**	78 ± 8%	>128	>128	>128	>128
4	**28**	70 ± 9%	>128	>128	128	>128
5	**30**	94 ± 7%	>128	>128	64	128
6	**32**	98 ± 8%	>128	>128	>128	>128
7	**33**	55 ± 9%	>128	>128	64	128
8	**aztreonam**	4.93 ± 0.42 μM	>128	0.5	0.5	0.5

^1^ Residual activity after 30-min preincubation.

## Data Availability

The original contributions presented in this study are included in the article/Supplementary Materials. Further inquiries can be directed to the corresponding author.

## Supplementary Materials

The following supporting information can be downloaded at https://www.mdpi.com/article/10.3390/molecules31203637/s1, ^1^H and ^13^C NMR spectra, HRMS and HPLC spectra for final compounds.

## Abbreviations

The following abbreviations are used in this manuscript:

AcOH	acetic acid
AMR	antimicrobial resistance
ATMO	(*Z*)-2-((((2-aminothiazol-4-yl)(carboxy)methylene)amino)oxy)-2-methylpropanoic acid
Boc_2_O	di-*tert*-butyl dicarbonate
CbzCl	benzyl chloroformate
CLSI	Clinical and Laboratory Standards Institute
DCM	dichloromethane
DIPEA	*N*,*N*-diisopropylethylamine
DMB	2,4-dimethoxybenzyl
DMF	dimethylformamide
EA	ethyl acetate
EUCAST	European Committee on Antimicrobial Susceptibility Testing
MeCN	acetonitrile
MeOH	methanol
MIC	minimum inhibitory concentration
PBP	penicillin-binding proteins
Ph_3_P	triphenylphosphine
TBAN3	tetrabutylammonium azide
TFA	trifluoroacetic acid
