# Supplementary material for: Synthesis of 3-Amino-4-(Hydroxymethyl)-β-Lactam Branch Point and Its Late-Stage C4 Functionalization with Ceftaroline-Sidechain Monobactams and Their Biological Evaluation
Source: Molecules. 2026 Oct 12;31(20):3637. doi: 10.3390/molecules31203637 (PMC13707450; doi:10.3390/molecules31203637)
Supplement: Supplementary file 1 [file molecules-31-03637-s001.zip › molecules-4571690-supplementary.pdf]

Supplementary Materials

Synthesis of 3-Amino-4-(Hydroxymethyl)-β-Lactam Branch Point and Its Late-Stage C4 Functionalization with Ceftaroline-Sidechain Monobactams and Their Biological Evaluation

Vid Kavaš[1], Majda Golob[2], Anže Meden[1], Martina Hrast Rambaher[1], Stanislav Gobec[1]*

[1] University of Ljubljana, Faculty of Pharmacy, Department of Pharmaceutical Chemistry, Aškerčeva cesta 7, 1000 Ljubljana, Slovenia.

[2] University of Ljubljana, Veterinary Faculty, Institute of Microbiology and Parasitology, Gerbičeva 60, 1000 Ljubljana, Slovenia

^{1}H and ^{13}C NMR spectra, HRMS and HPLC spectra for final compounds

Compound **1**

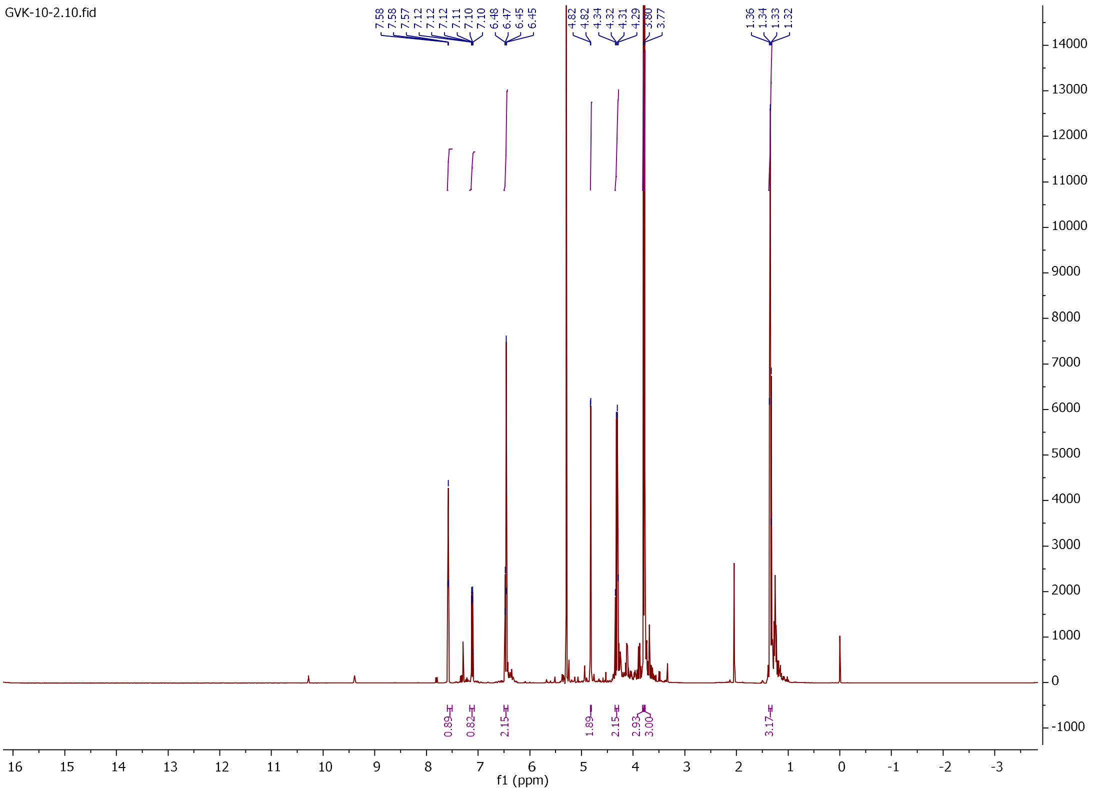

Compound **2**

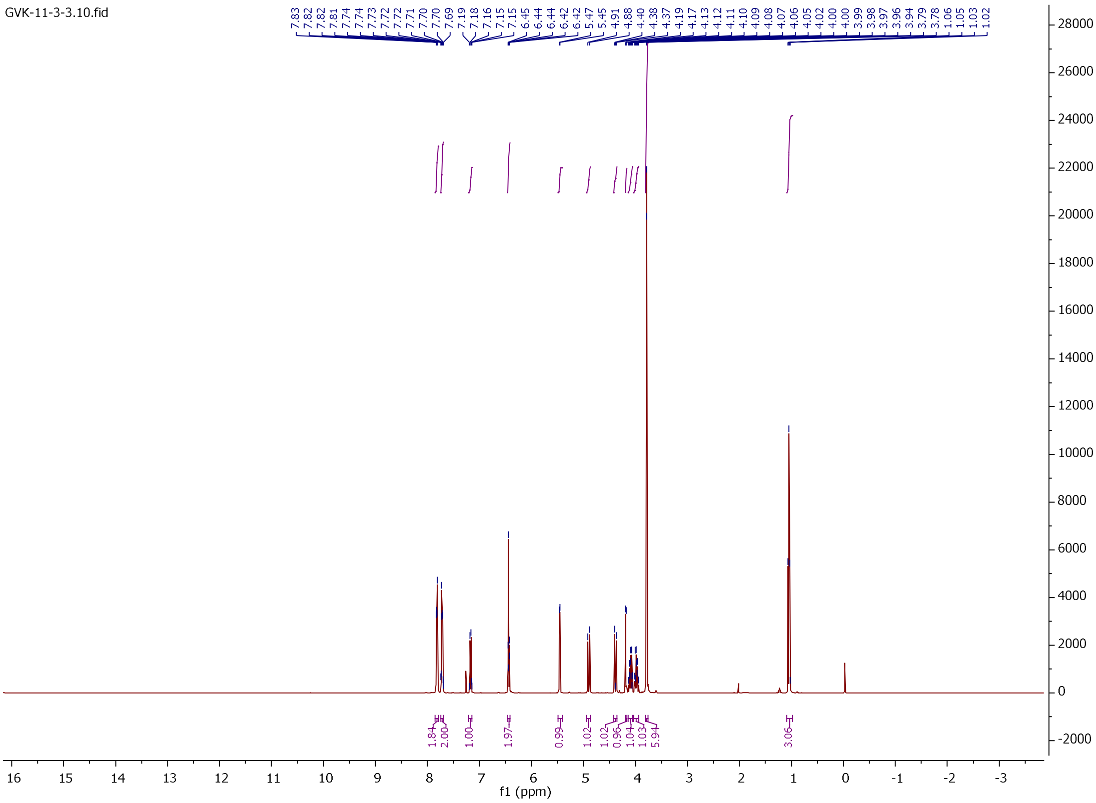

Compound **3**

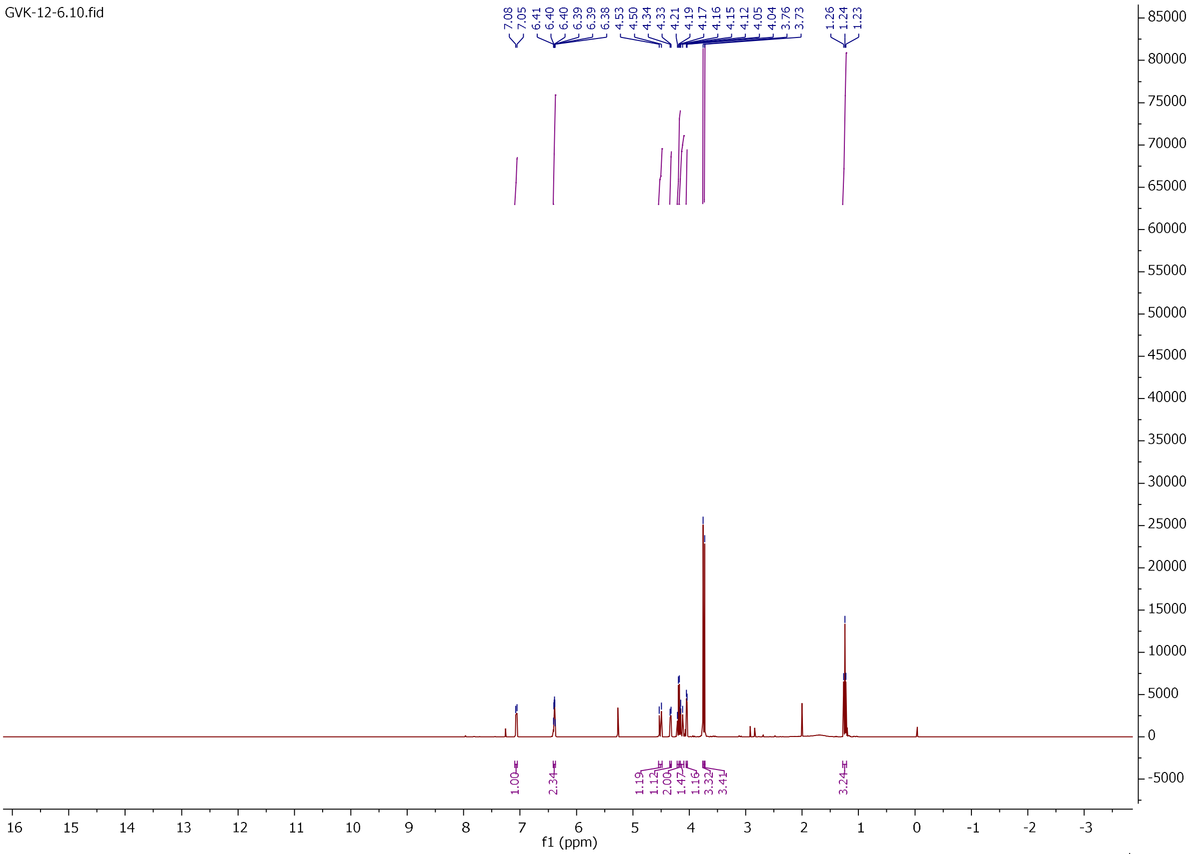
GVK-12-6.10.fid
7.08
7.05
6.41
6.40
6.40
6.39
6.39
6.38
4.53
4.50
4.34
4.33
4.21
4.19
4.17
4.16
4.15
4.12
4.05
4.04
3.76
3.73
1.26
1.24
1.23
1.00
2.34
1.19
1.13
2.00
1.47
1.16
3.32
3.41
3.24
f1 (ppm)

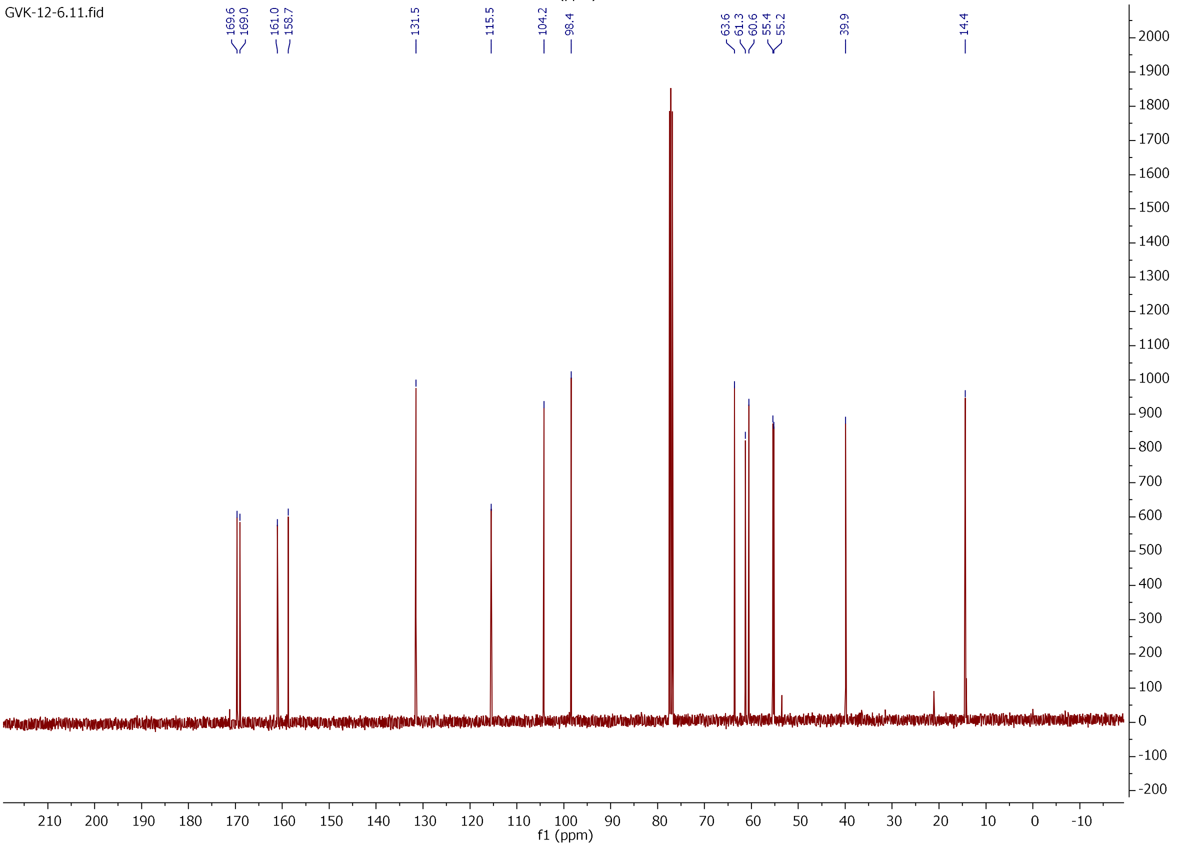
GVK-12-6.11.fid
169.6
169.0
161.0
158.7
131.5
115.5
104.2
98.4
63.6
61.3
60.6
55.4
55.2
39.9
14.4
f1 (ppm)

Compound **4**

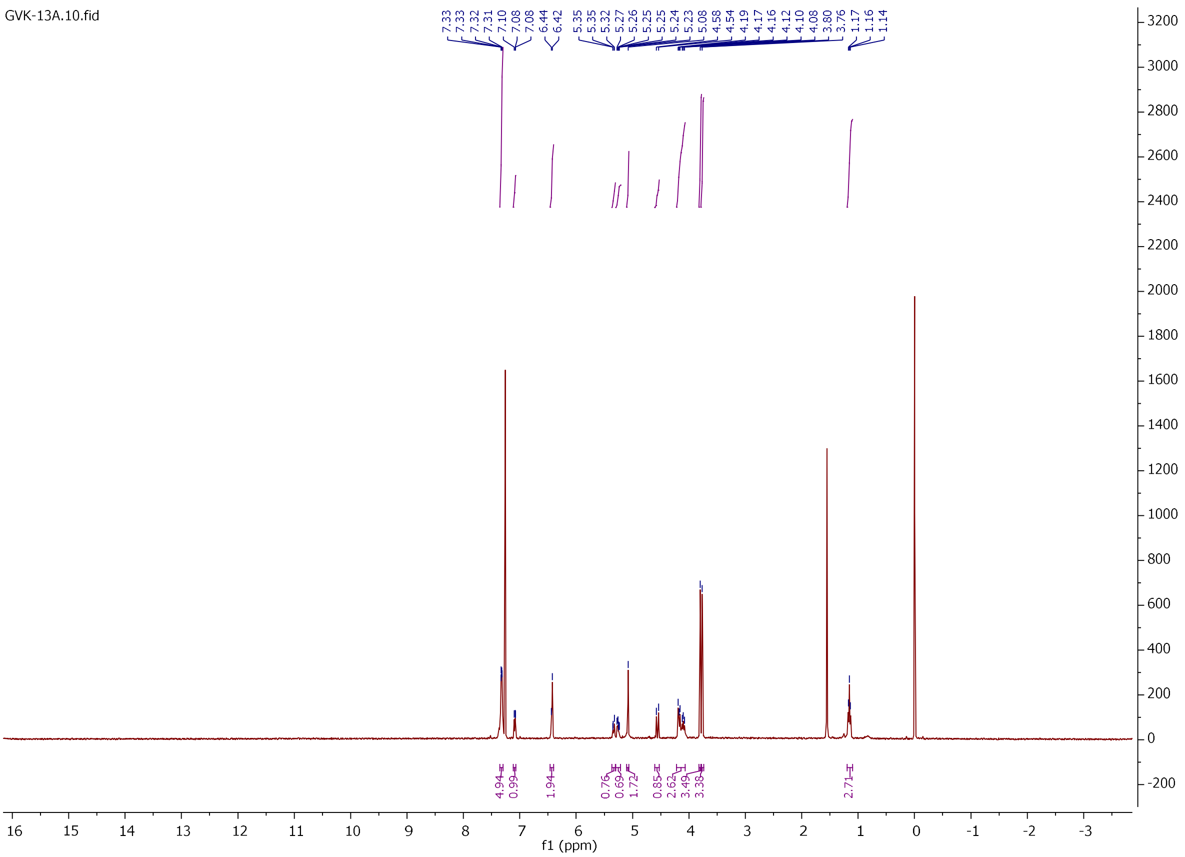

Compound **5**

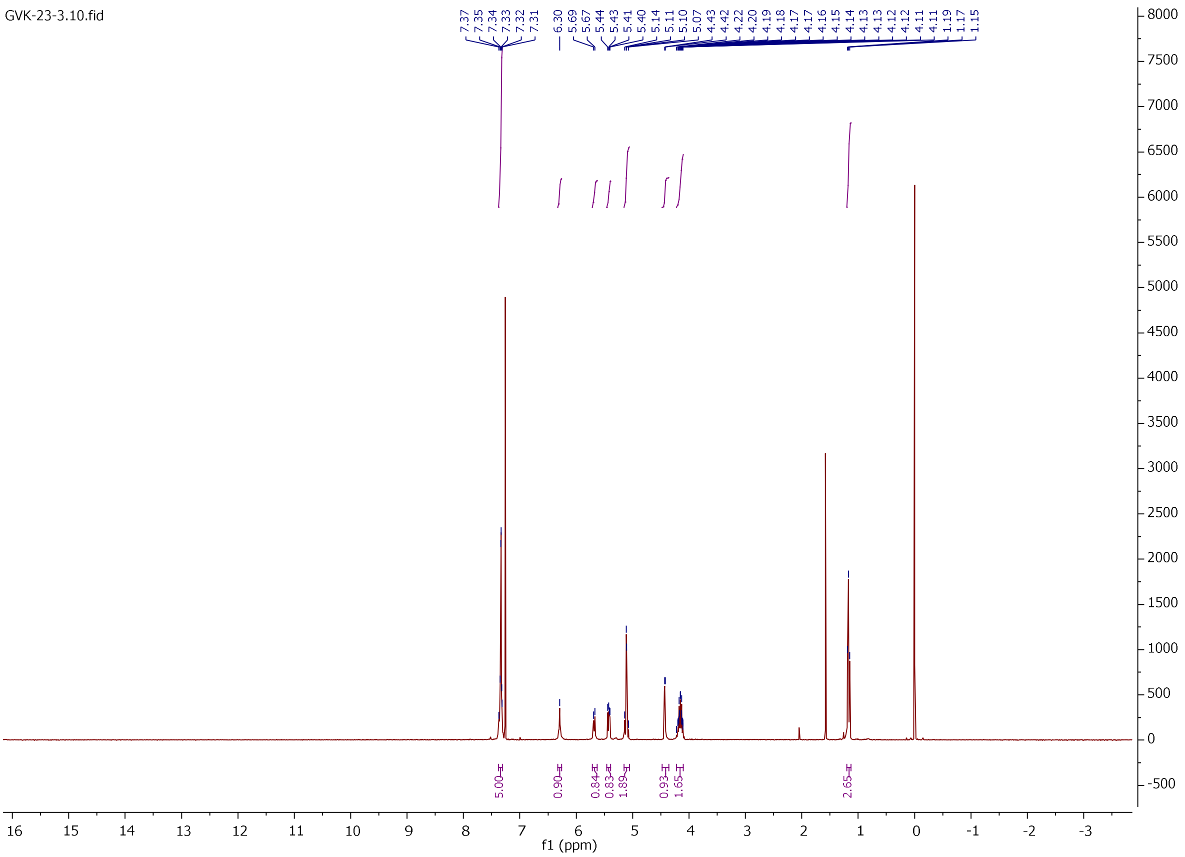

Compound **6**

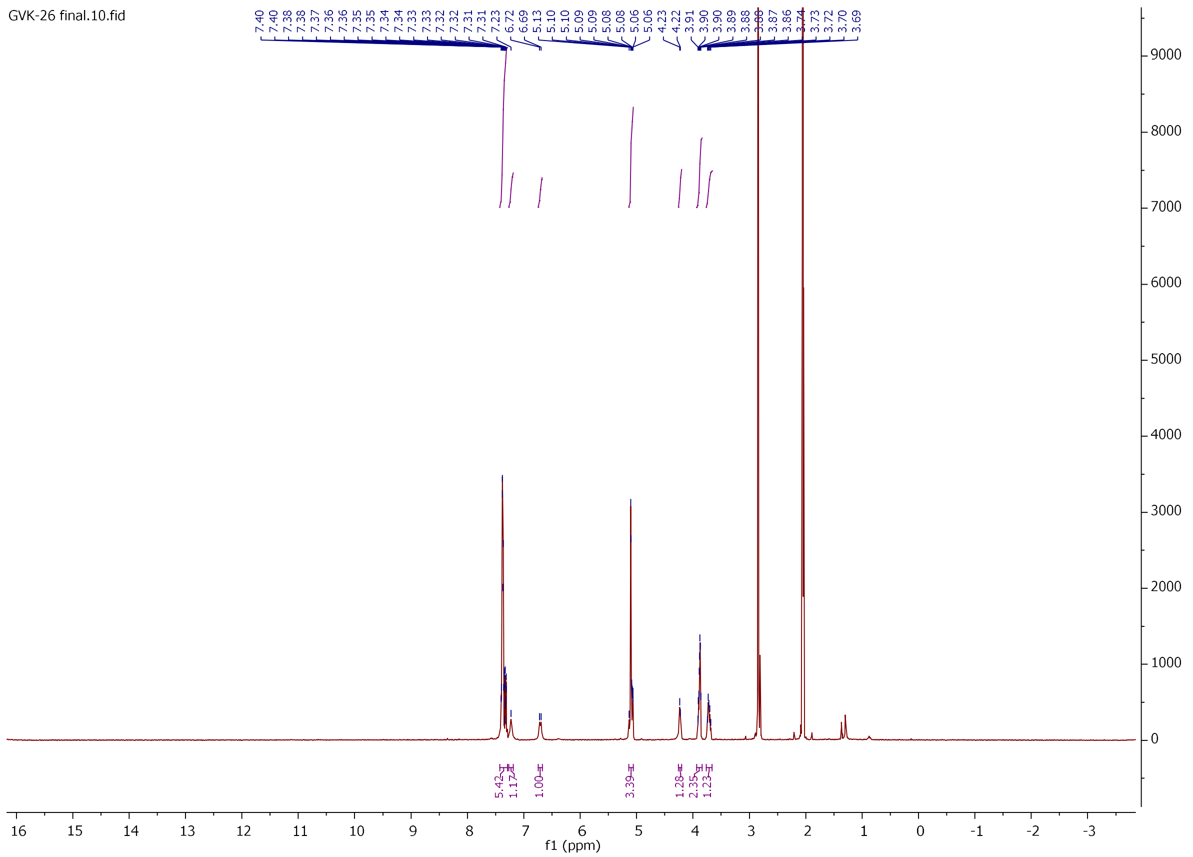
GVK-26 final.10.fid
7.40
7.40
7.38
7.38
7.37
7.36
7.36
7.35
7.35
7.34
7.34
7.33
7.33
7.32
7.32
7.31
7.31
7.23
6.72
6.69
5.13
5.10
5.10
5.09
5.09
5.08
5.08
5.06
5.06
4.23
4.22
3.91
3.90
3.90
3.89
3.88
3.88
3.87
3.86
3.74
3.73
3.72
3.70
3.69
5.42
1.17
1.00
3.39
1.28
2.35
1.23
f1 (ppm)

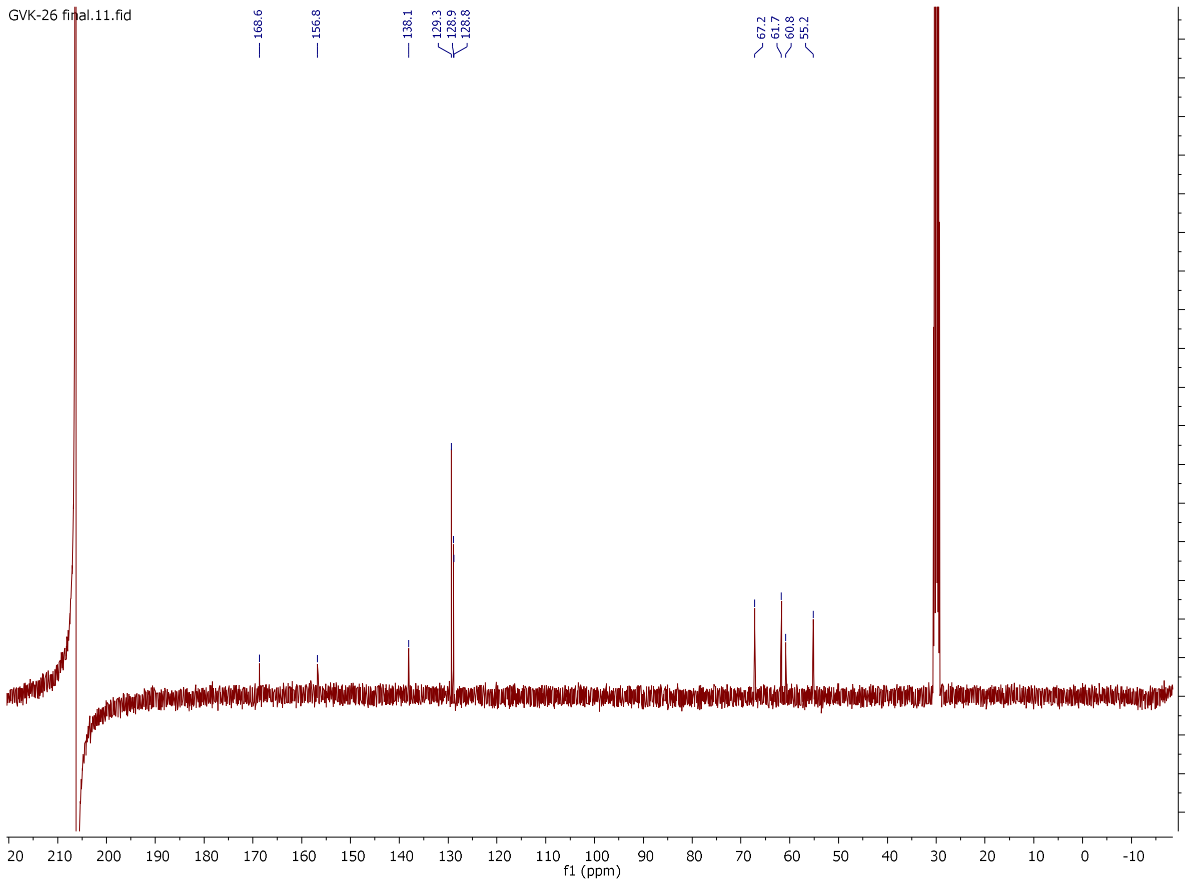
GVK-26 final.11.fid
168.6
156.8
138.1
129.3
128.9
128.8
67.2
61.7
60.8
55.2
f1 (ppm)

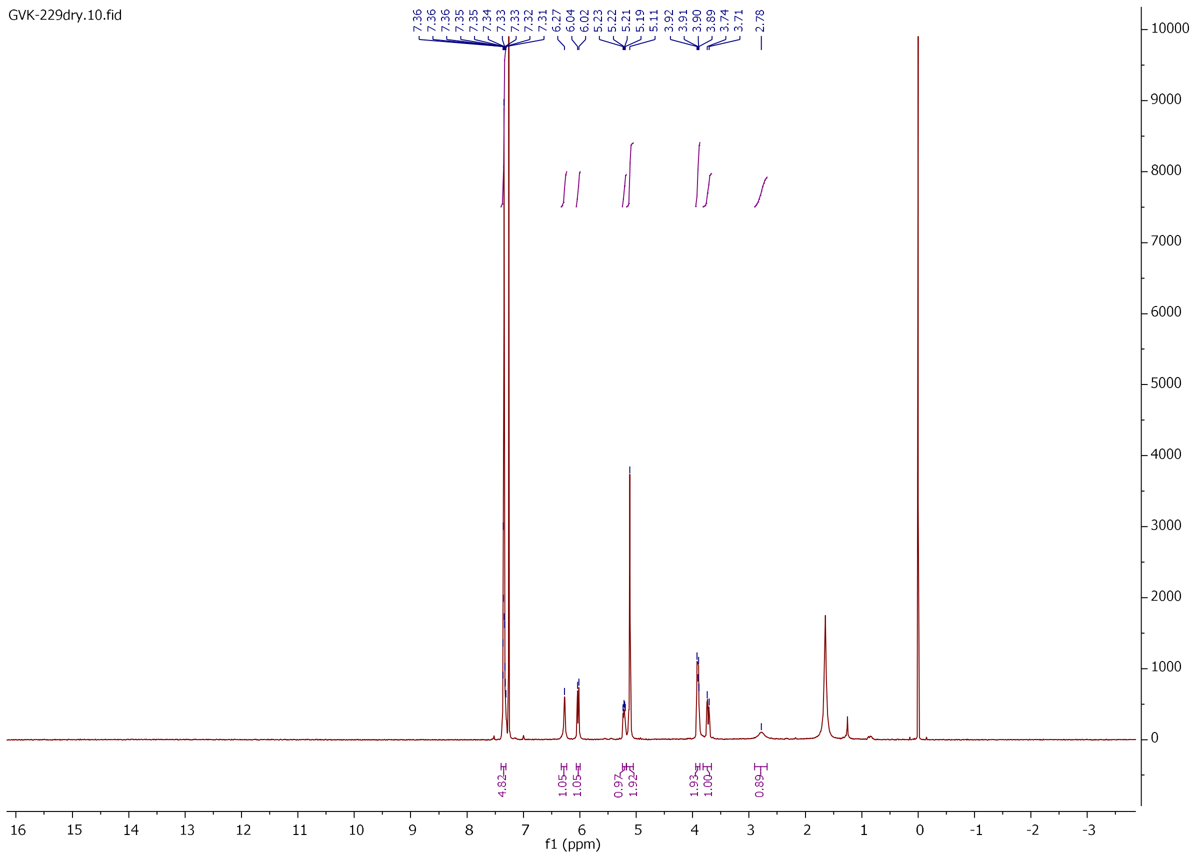
GVK-229dry.10.fid
7.36 7.36 7.36 7.35 7.35 7.34 7.33 7.33 7.32 7.31 6.27 6.04 6.02 5.23 5.22 5.21 5.19 5.11 3.92 3.91 3.90 3.89 3.74 3.71 2.78
4.82 1.05 1.05 0.97 1.92 1.93 1.00 0.89
f1 (ppm)

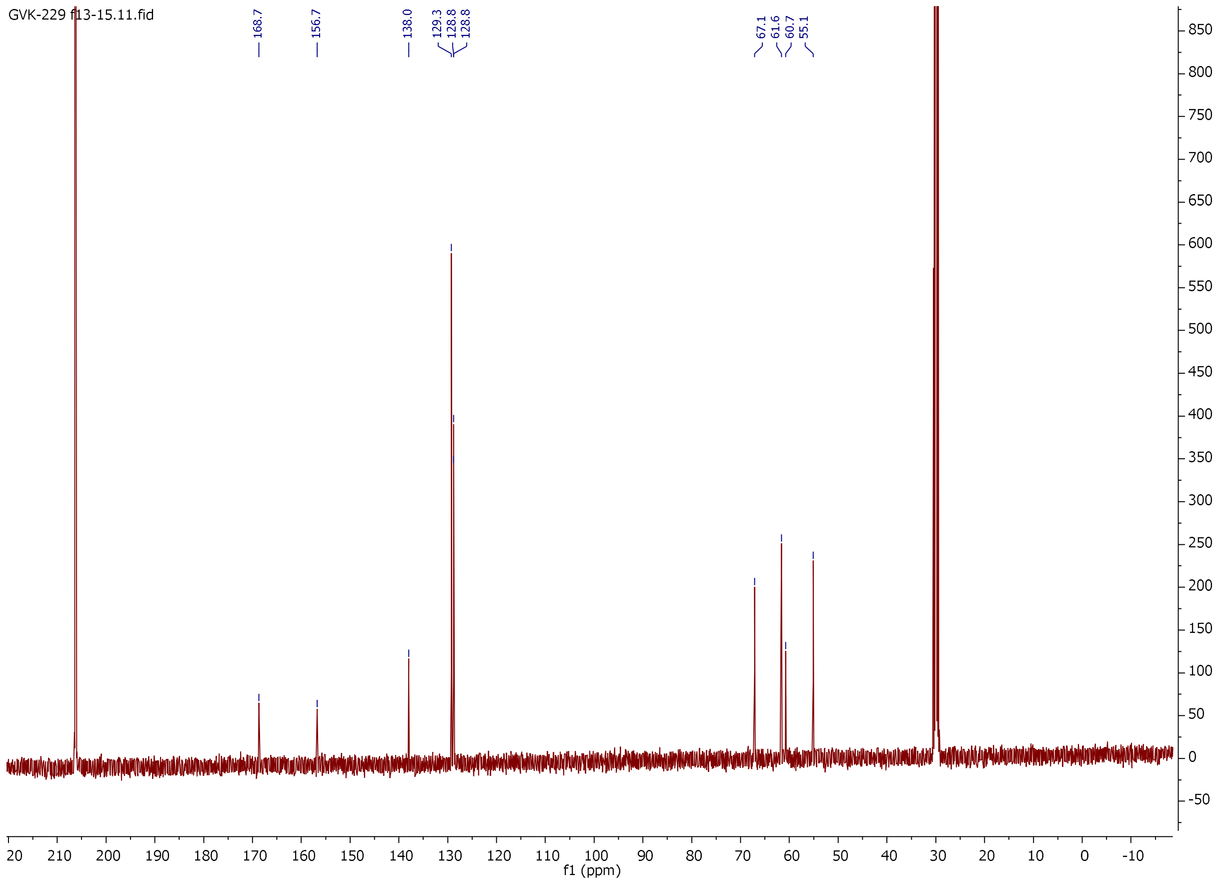
GVK-229 f13-15.11.fid
168.7 156.7 138.0 129.3 128.8 128.8 67.1 61.6 60.7 55.1
f1 (ppm)

Compound **7**

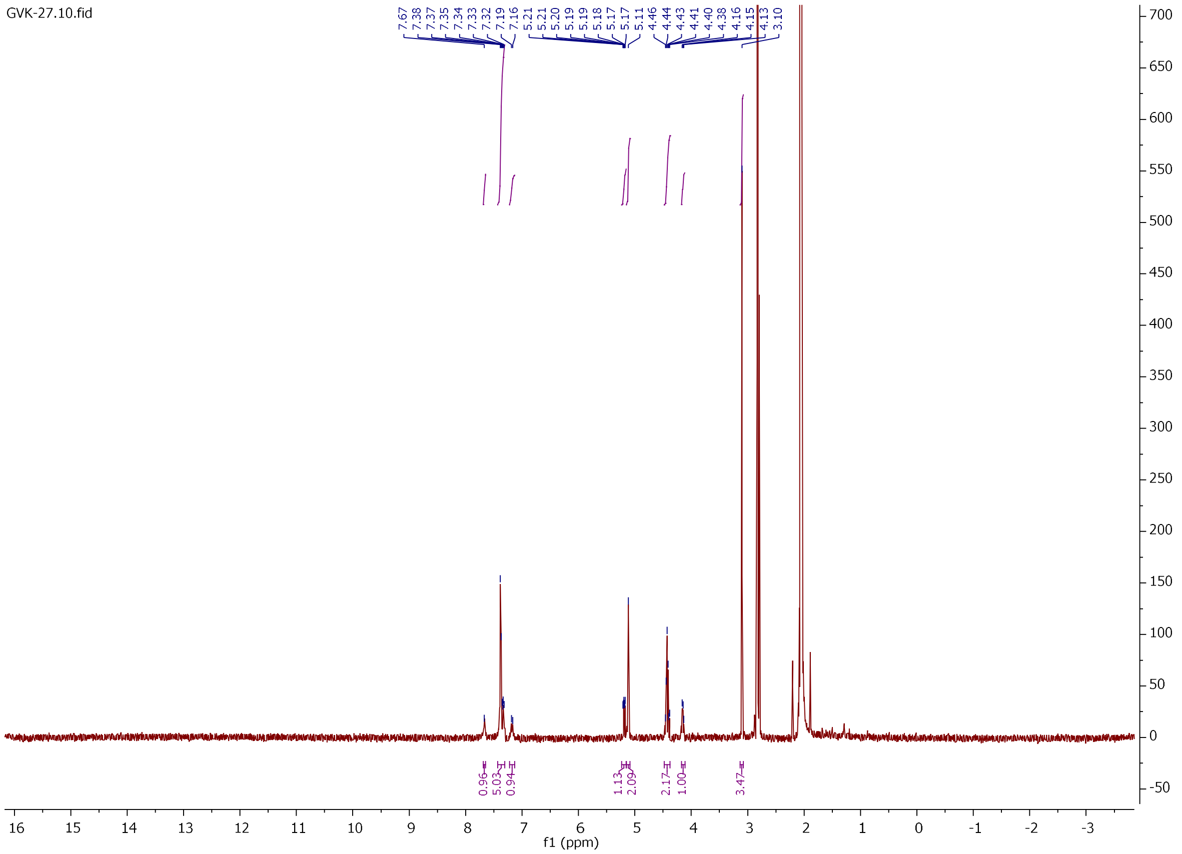
GVK-27.10.fid
7.67
7.38
7.37
7.35
7.34
7.33
7.32
7.19
7.16
5.21
5.21
5.20
5.19
5.19
5.18
5.17
5.17
5.11
4.46
4.44
4.43
4.41
4.40
4.38
4.16
4.15
4.13
3.10
0.96
5.03
0.94
1.13
2.09
2.17
1.00
3.47
f1 (ppm)

Compound **8**

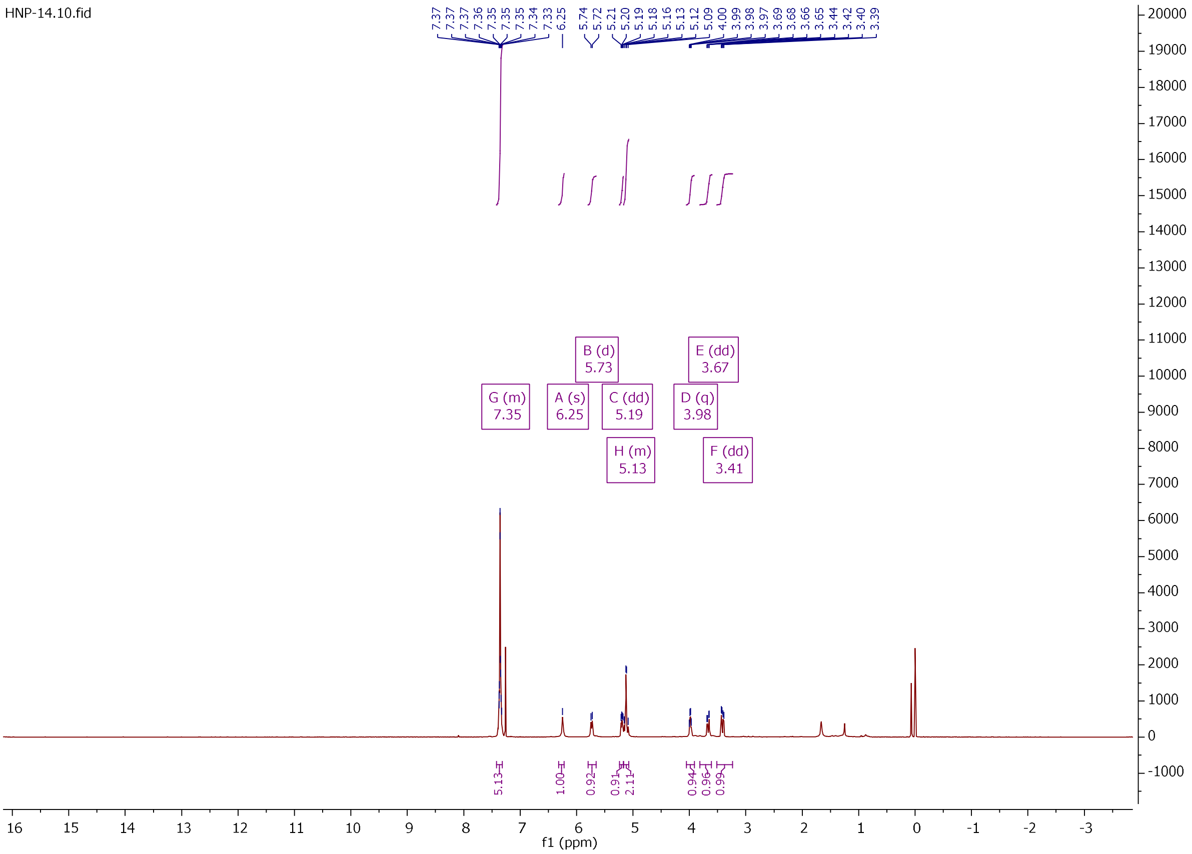

HNP-14.10.fid
7.37 7.37 7.37 7.36 7.35 7.35 7.35 7.34 7.33 6.25 5.74 5.72 5.21 5.20 5.19 5.18 5.16 5.13 5.12 5.09 4.00 3.99 3.98 3.97 3.69 3.68 3.66 3.65 3.44 3.42 3.40 3.39
B (d) 5.73
E (dd) 3.67
G (m) 7.35
A (s) 6.25
C (dd) 5.19
D (q) 3.98
H (m) 5.13
F (dd) 3.41
5.13 1.00 0.92 0.91 2.11 0.94 0.96 0.99
f1 (ppm)

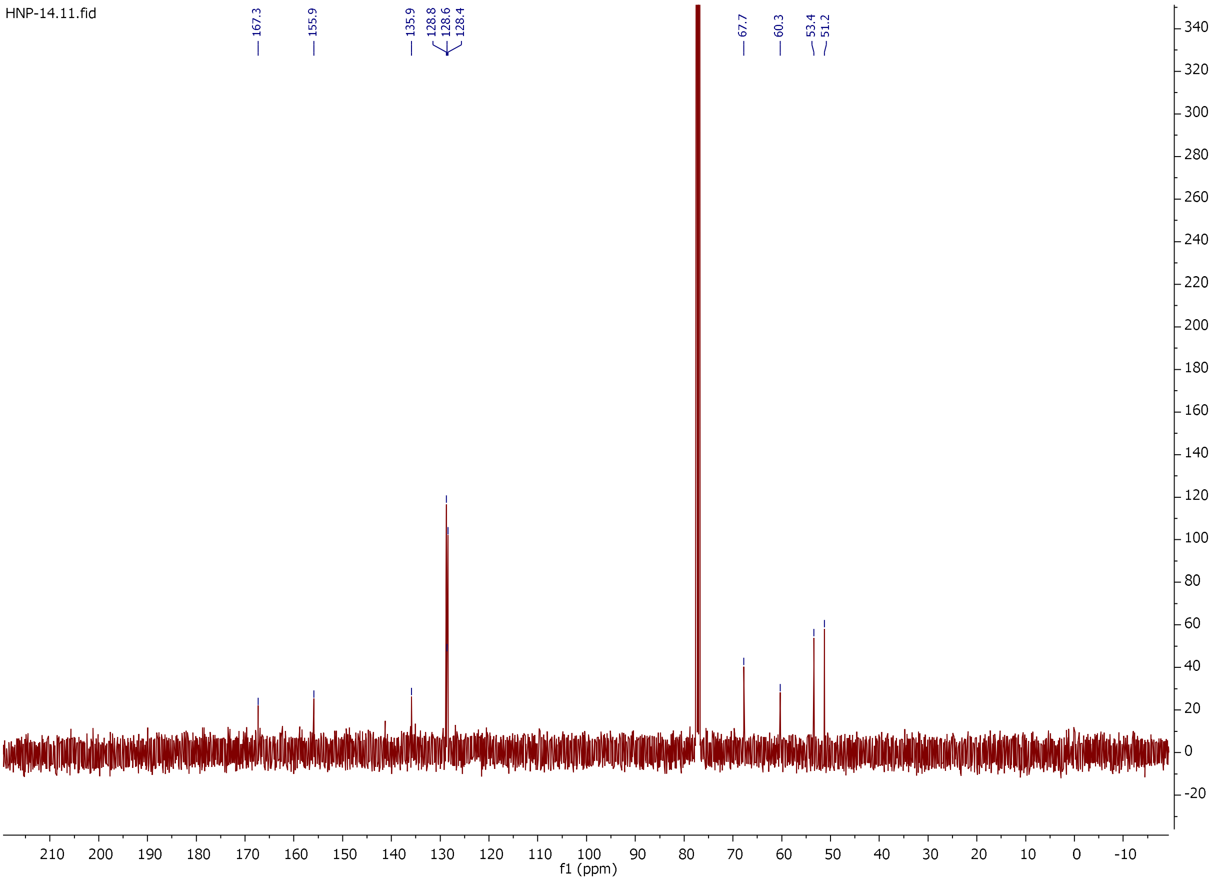

HNP-14.11.fid
167.3 155.9 135.9 128.8 128.6 128.4 67.7 60.3 53.4 51.2
f1 (ppm)

Compound **9**

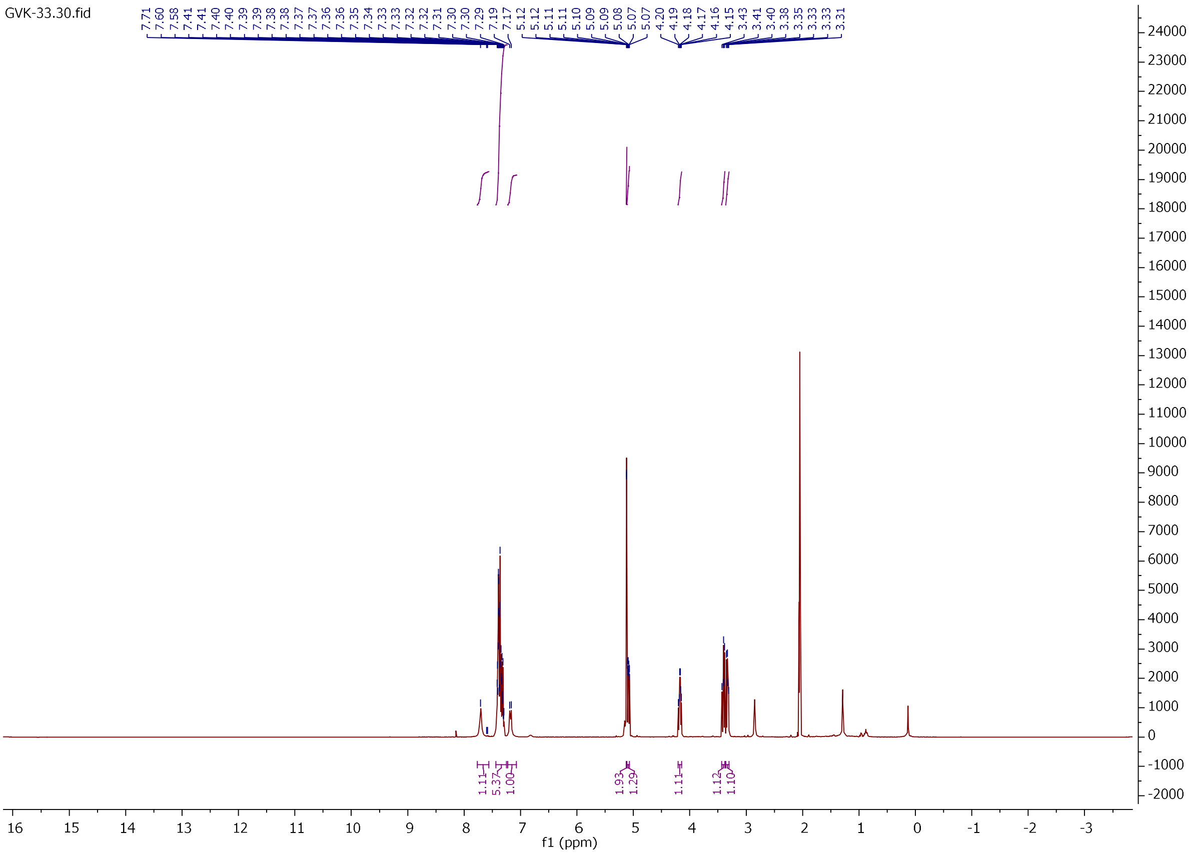
GVK-33.30.fid
7.71
7.60
7.58
7.41
7.41
7.40
7.40
7.39
7.39
7.38
7.38
7.37
7.37
7.36
7.36
7.35
7.34
7.33
7.33
7.32
7.32
7.31
7.30
7.30
7.29
7.19
7.17
5.12
5.12
5.11
5.11
5.10
5.09
5.08
5.07
5.07
4.20
4.19
4.18
4.17
4.16
4.15
3.43
3.41
3.40
3.38
3.35
3.33
3.33
3.31
1.11
5.37
1.00
1.93
1.29
1.11
1.12
1.10
f1 (ppm)

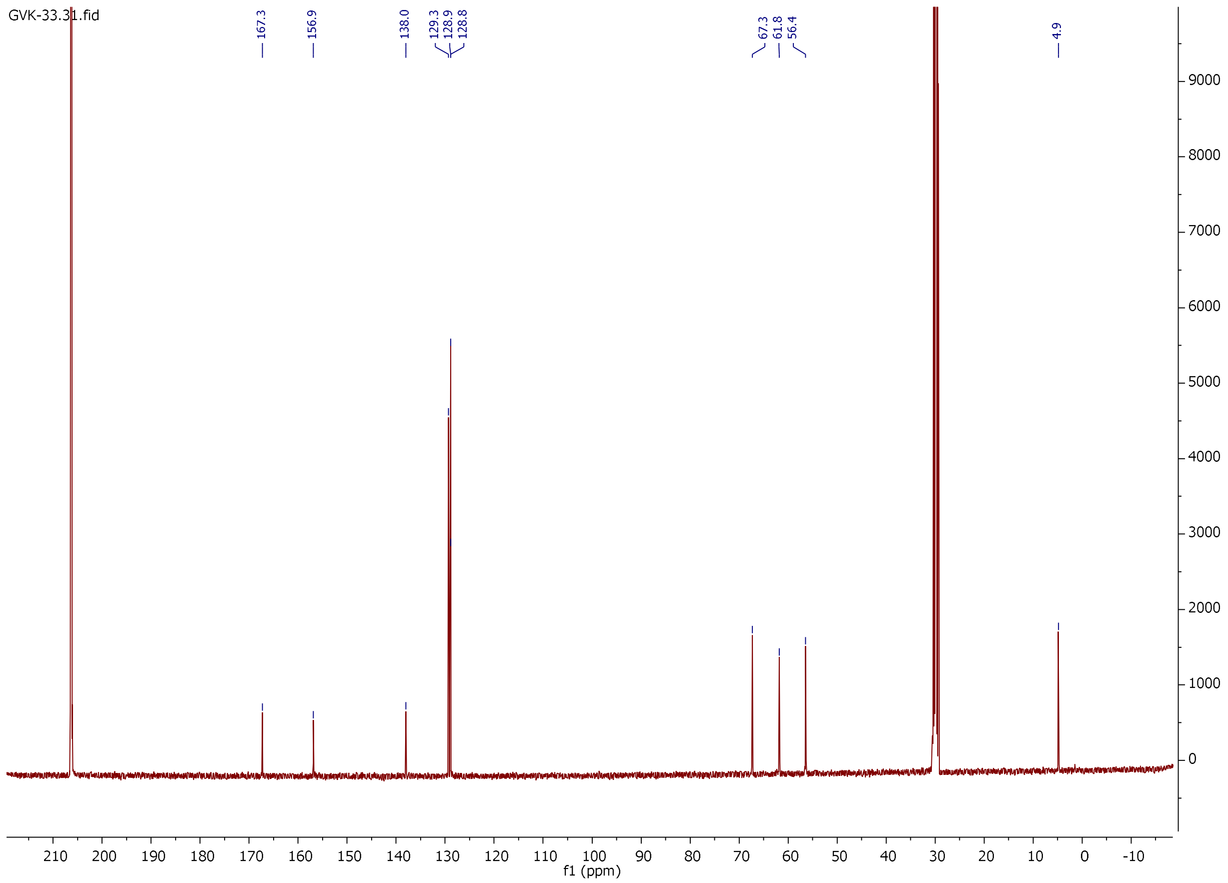
GVK-33.31.fid
167.3
156.9
138.0
129.3
128.9
128.8
67.3
61.8
56.4
4.9
9000
8000
7000
6000
5000
4000
3000
2000
1000
0
210 200 190 180 170 160 150 140 130 120 110 100 90 80 70 60 50 40 30 20 10 0 -10
f1 (ppm)

Compound **10**

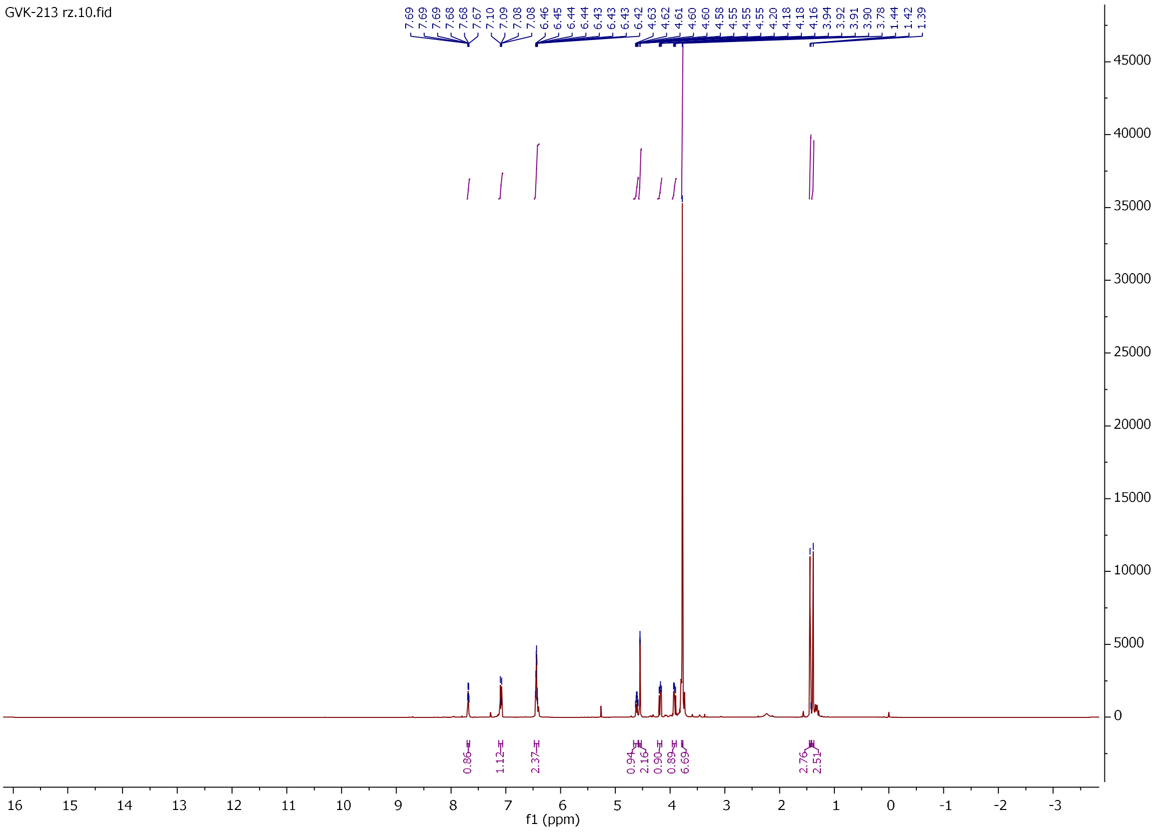

Compound **11**

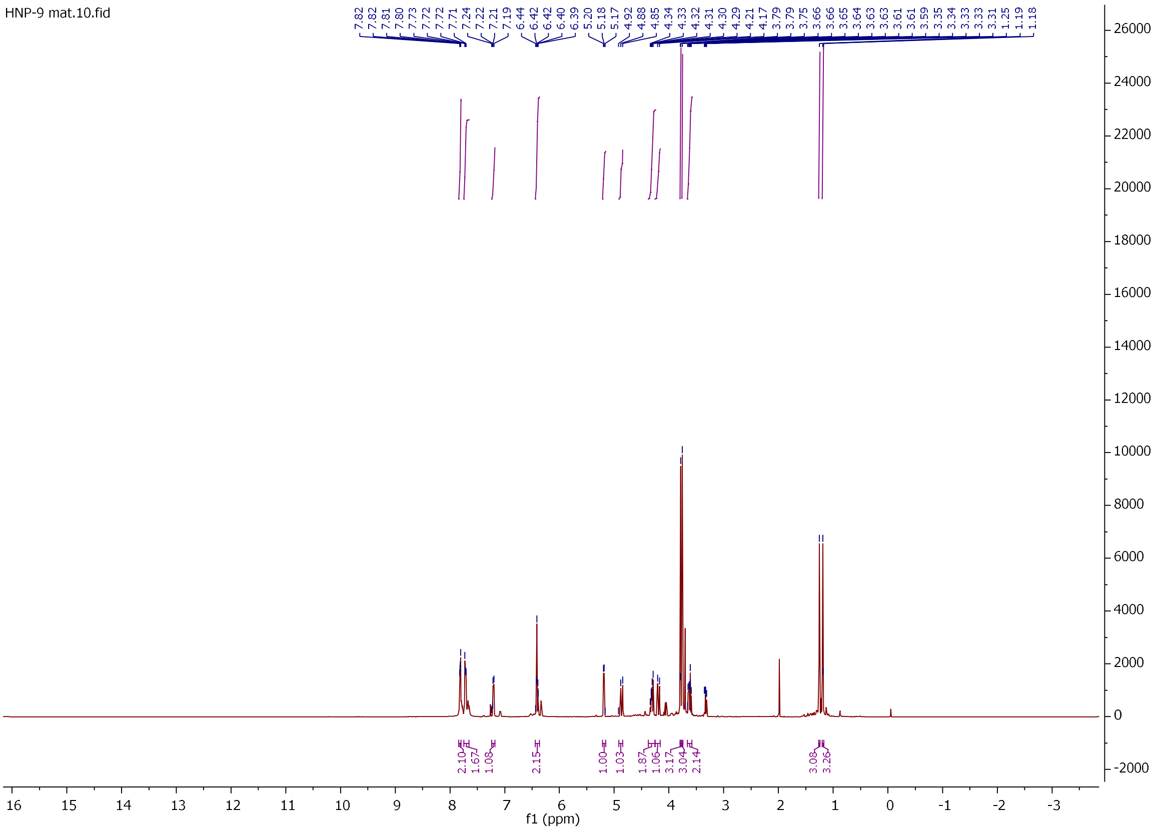

Compound **13**

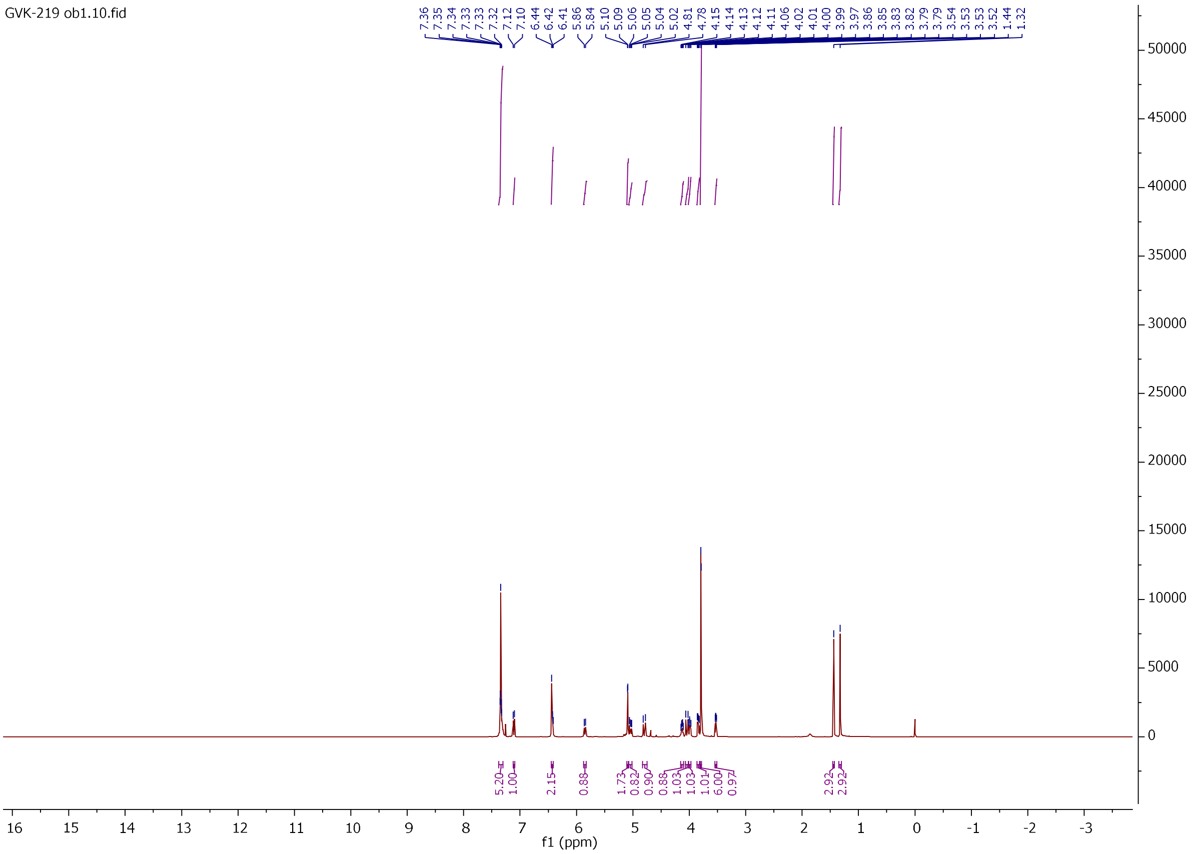
GVK-219 ob1.10.fid
7.36
7.35
7.34
7.33
7.33
7.32
7.12
7.10
6.44
6.42
6.41
5.86
5.84
5.10
5.09
5.06
5.05
5.04
5.02
4.81
4.78
4.15
4.14
4.13
4.12
4.11
4.06
4.02
4.01
4.00
3.99
3.97
3.86
3.85
3.83
3.82
3.79
3.79
3.54
3.53
3.53
3.52
1.44
1.32
5.20
1.00
2.15
0.88
1.73
0.82
0.90
0.88
1.03
1.03
1.01
6.00
0.97
2.92
2.92
f1 (ppm)

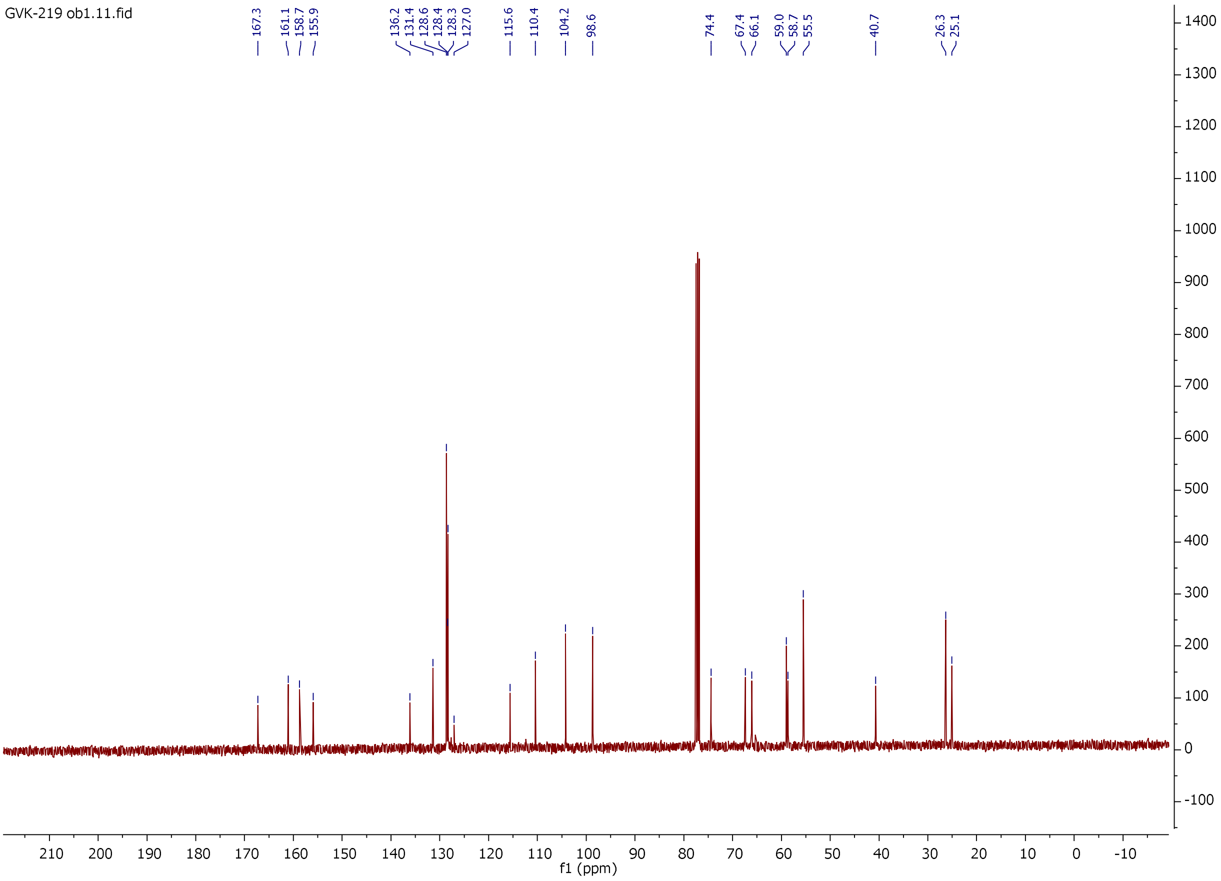
GVK-219 ob1.11.fid
167.3
161.1
158.7
155.9
136.2
131.4
128.6
128.4
128.3
127.0
115.6
110.4
104.2
98.6
74.4
67.4
66.1
59.0
58.7
55.5
40.7
26.3
25.1
f1 (ppm)

Compound **14**

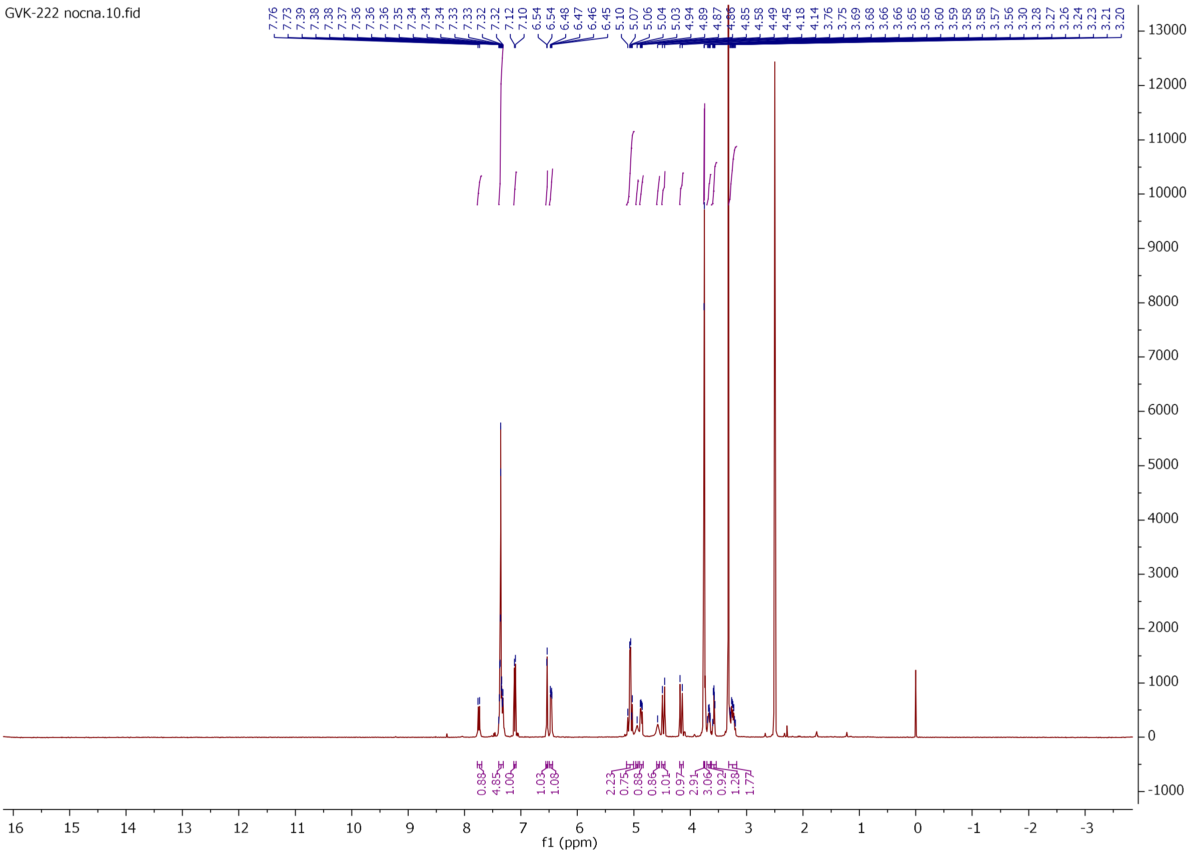
GVK-222 nocna.10.fid
7.76
7.73
7.39
7.38
7.38
7.37
7.36
7.36
7.35
7.34
7.34
7.33
7.32
7.32
7.12
7.10
6.54
6.54
6.48
6.47
6.46
6.45
5.10
5.07
5.06
5.04
5.03
4.94
4.89
4.87
4.86
4.85
4.58
4.49
4.45
4.18
4.14
3.76
3.75
3.69
3.68
3.66
3.66
3.65
3.60
3.59
3.58
3.58
3.57
3.56
3.30
3.28
3.27
3.26
3.24
3.23
3.21
3.20
0.88
4.85
1.00
1.03
1.08
2.23
0.75
0.88
0.86
1.01
0.97
2.91
3.06
0.92
1.28
1.77
f1 (ppm)

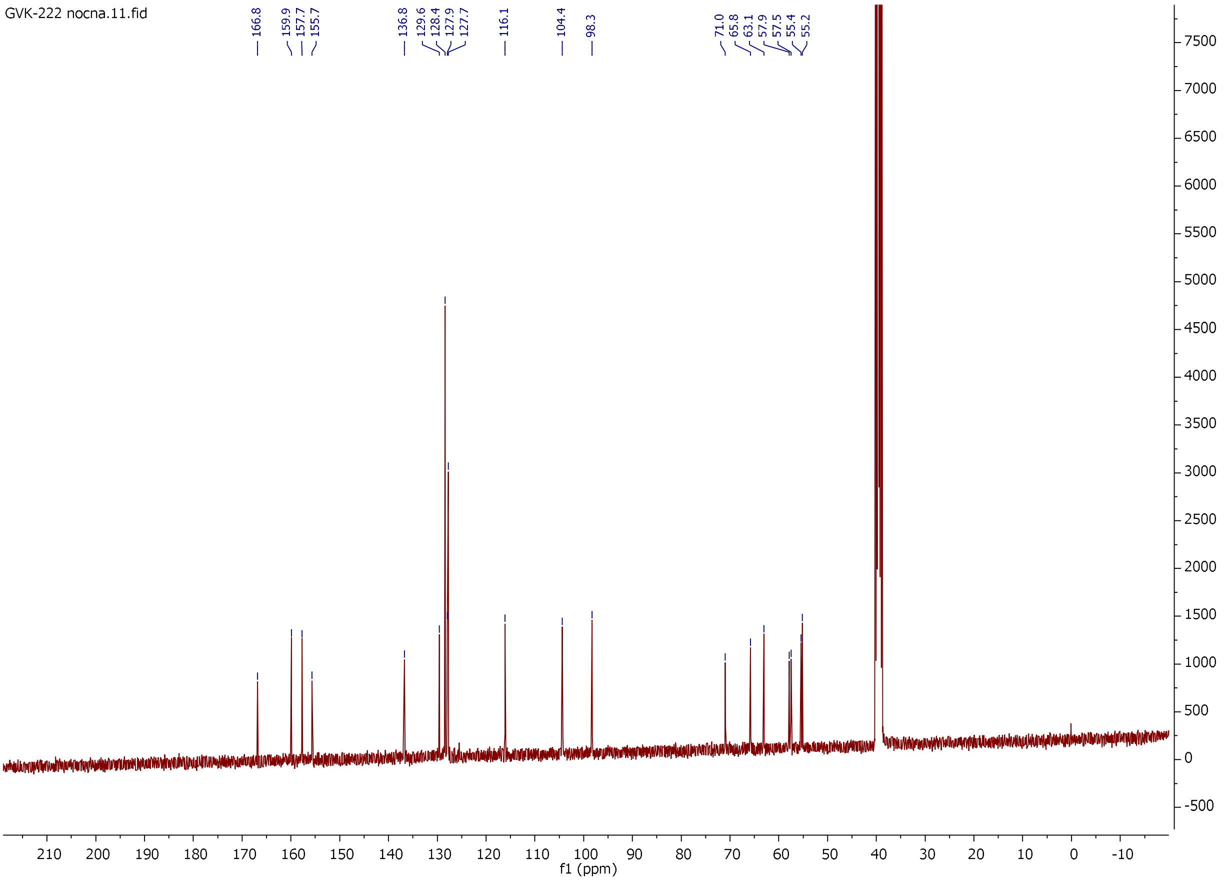
GVK-222 nocna.11.fid
166.8
159.9
157.7
155.7
136.8
129.6
128.4
127.9
127.7
116.1
104.4
98.3
71.0
65.8
63.1
57.9
57.5
55.4
55.2
f1 (ppm)

Compound **15**

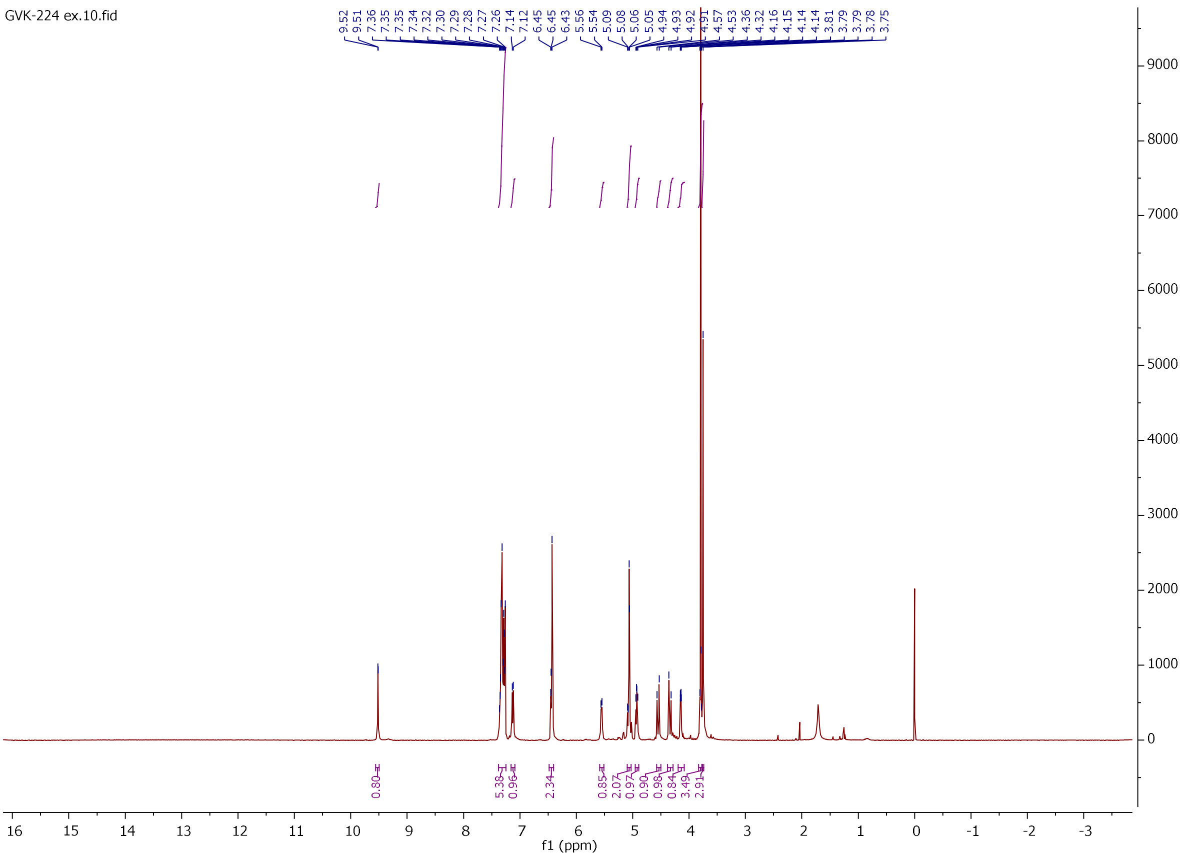
GVK-224 ex.10.fid
9.52 9.51 7.36 7.35 7.35 7.34 7.32 7.30 7.29 7.28 7.27 7.26 7.14 7.12 6.45 6.45 6.43 5.56 5.54 5.09 5.08 5.06 5.05 4.94 4.93 4.92 4.91 4.57 4.53 4.36 4.32 4.16 4.15 4.14 4.14 3.81 3.79 3.78 3.75
0.80 5.38 0.96 2.34 0.85 2.07 0.97 0.90 0.98 0.84 3.49 2.91
f1 (ppm)

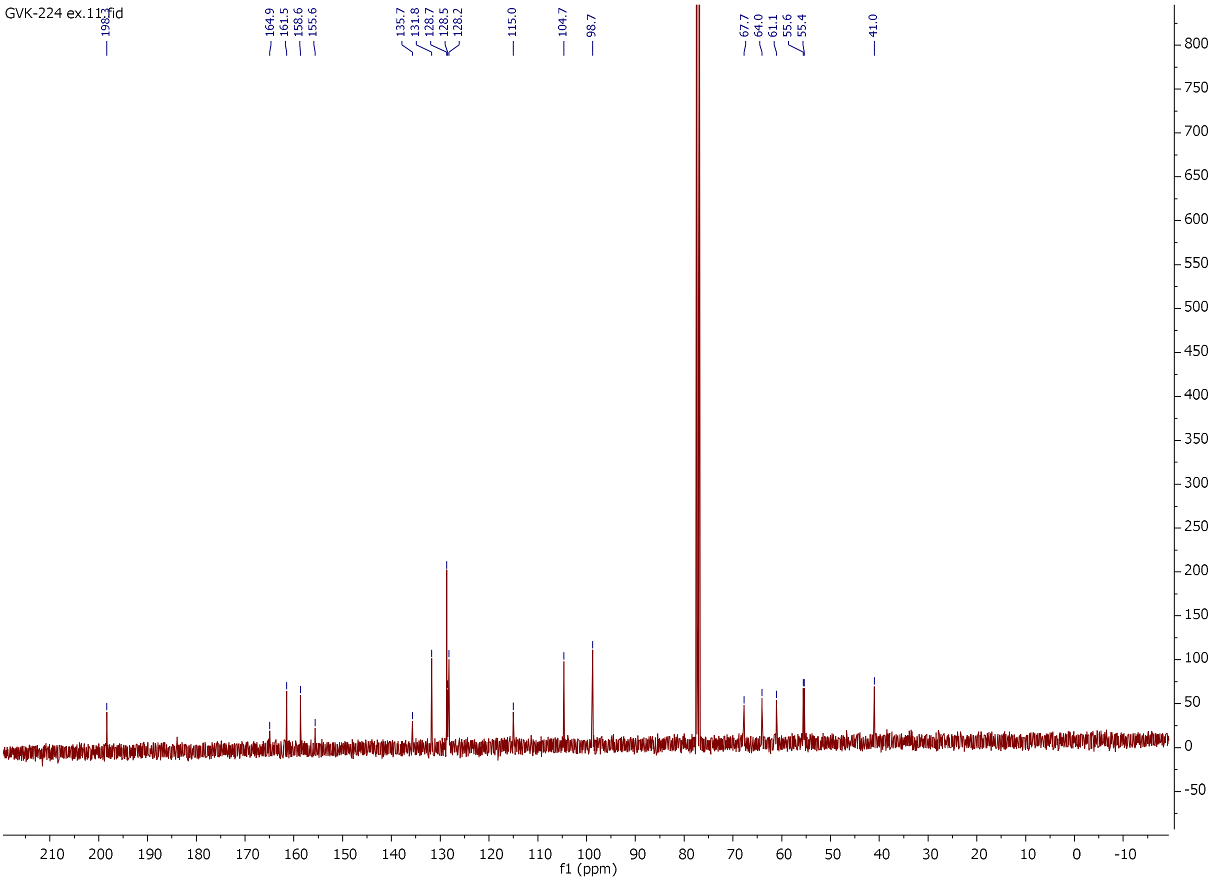
GVK-224 ex.11.fid
164.9 161.5 158.6 155.6 135.7 131.8 128.7 128.5 128.2 115.0 104.7 98.7 67.7 64.0 61.1 55.6 55.4 41.0
f1 (ppm)

Compound **16**

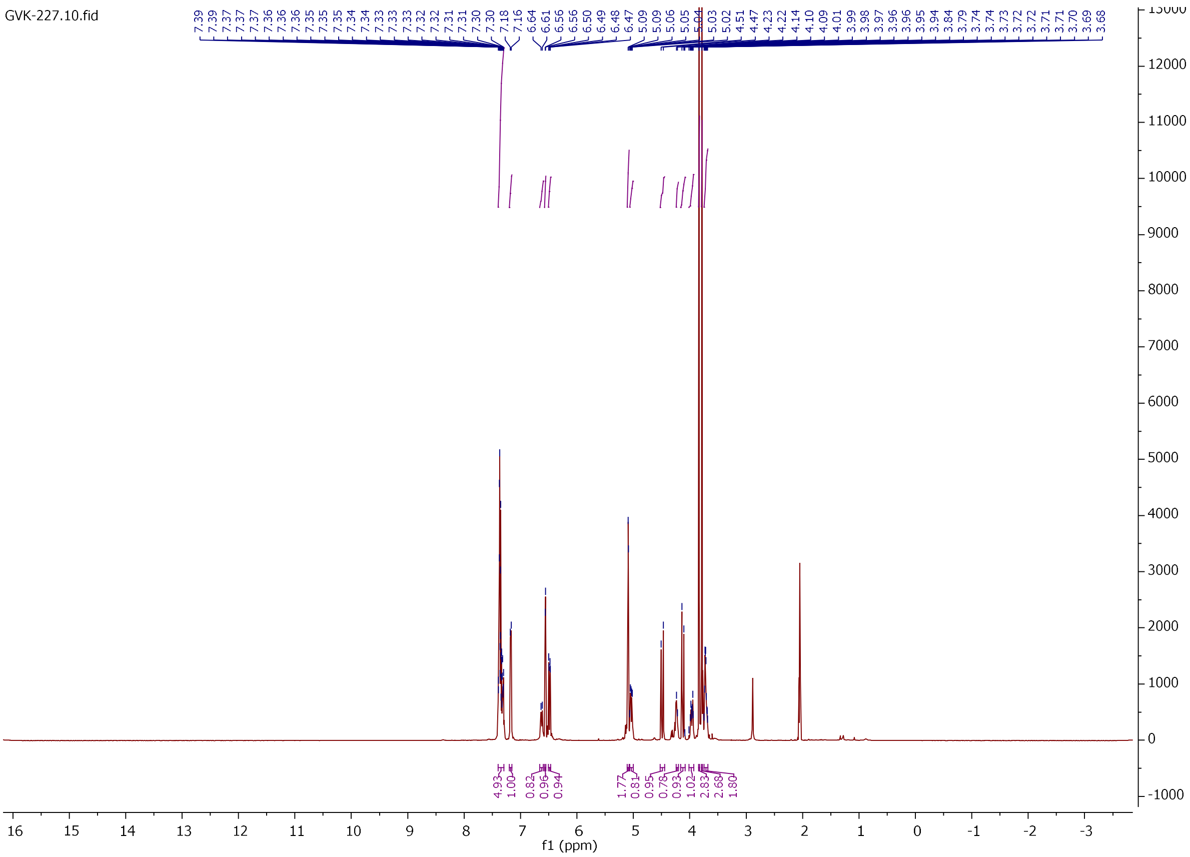
GVK-227.10.fid
7.39 7.39 7.37 7.37 7.37 7.36 7.36 7.36 7.35 7.35 7.35 7.34 7.34 7.33 7.33 7.33 7.32 7.32 7.31 7.31 7.30 7.30 7.18 7.16 6.64 6.61 6.56 6.56 6.50 6.49 6.48 6.47 5.09 5.09 5.06 5.05 5.04 5.03 5.02 4.51 4.47 4.23 4.22 4.14 4.10 4.09 4.01 3.99 3.98 3.97 3.96 3.96 3.95 3.94 3.84 3.79 3.74 3.74 3.73 3.72 3.72 3.71 3.70 3.69 3.68
4.93 1.00 0.82 0.96 0.94 1.77 0.81 0.95 0.78 0.93 1.02 2.83 2.68 1.80
f1 (ppm)

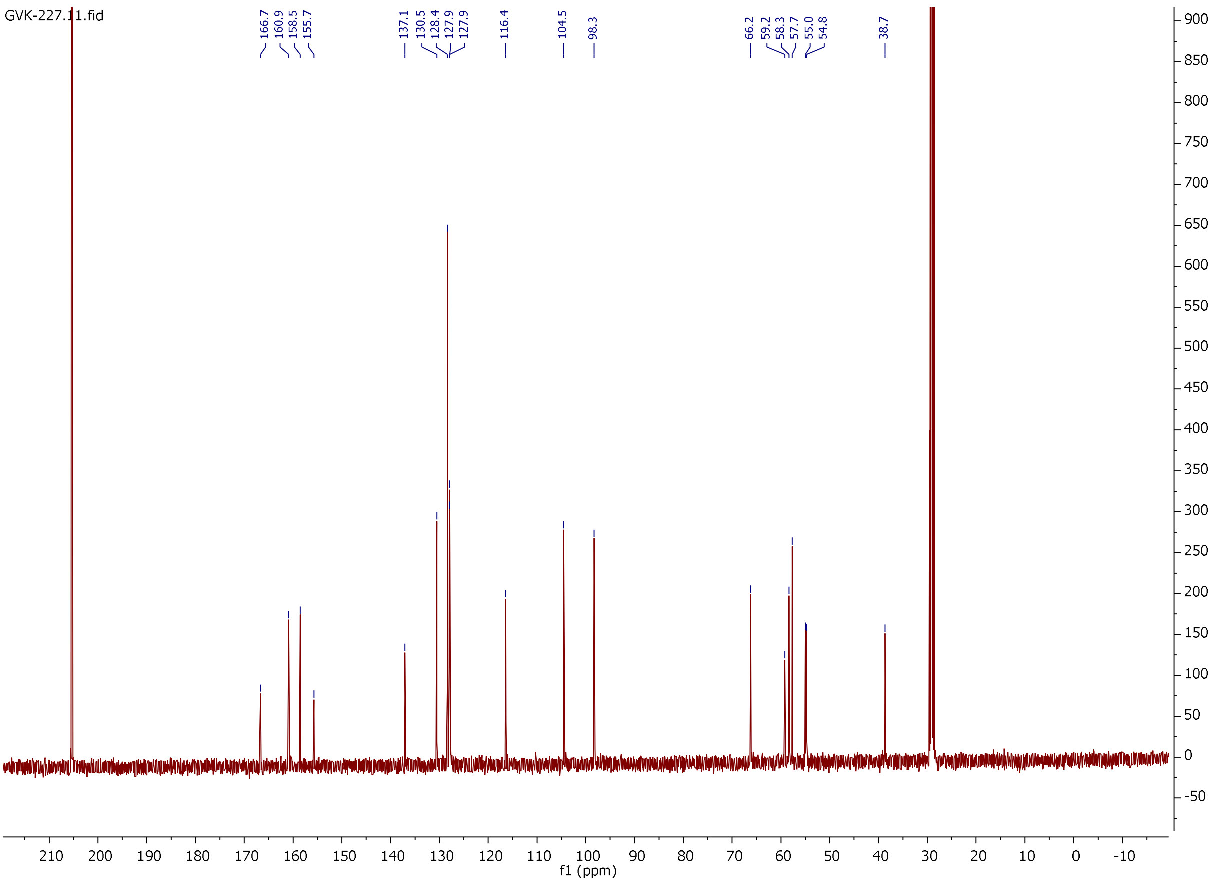
GVK-227.11.fid
166.7 160.9 158.5 155.7 137.1 130.5 128.4 127.9 127.9 116.4 104.5 98.3 66.2 59.2 58.3 57.7 55.0 54.8 38.7
f1 (ppm)

Compound **17**

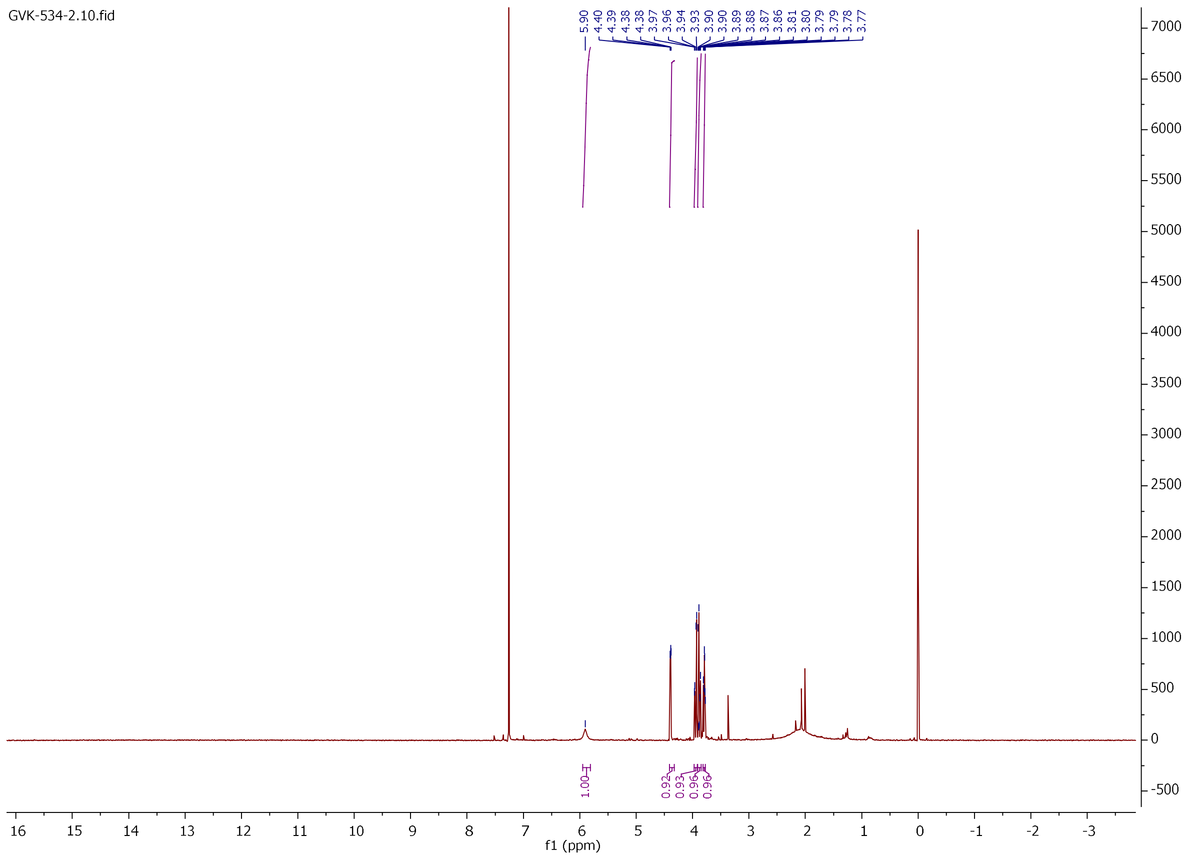

Compound **18a**

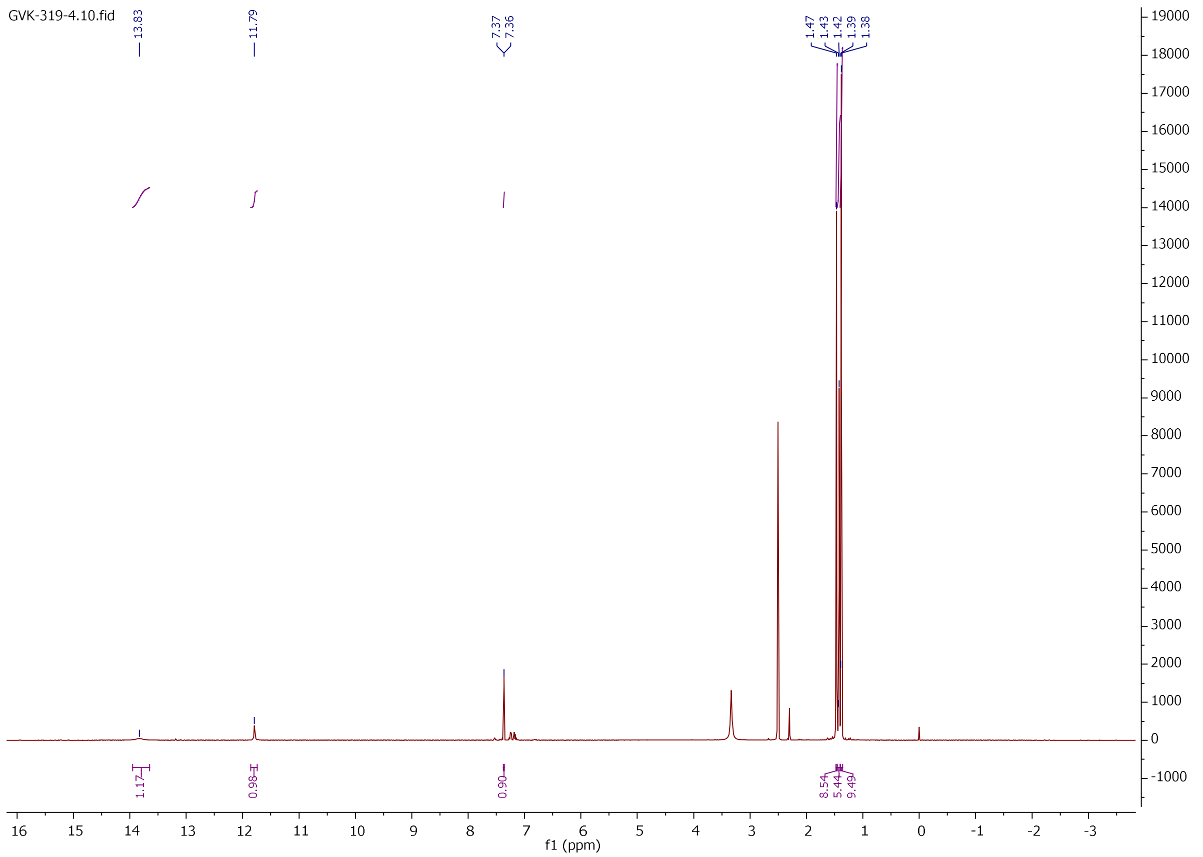

Compound **18**

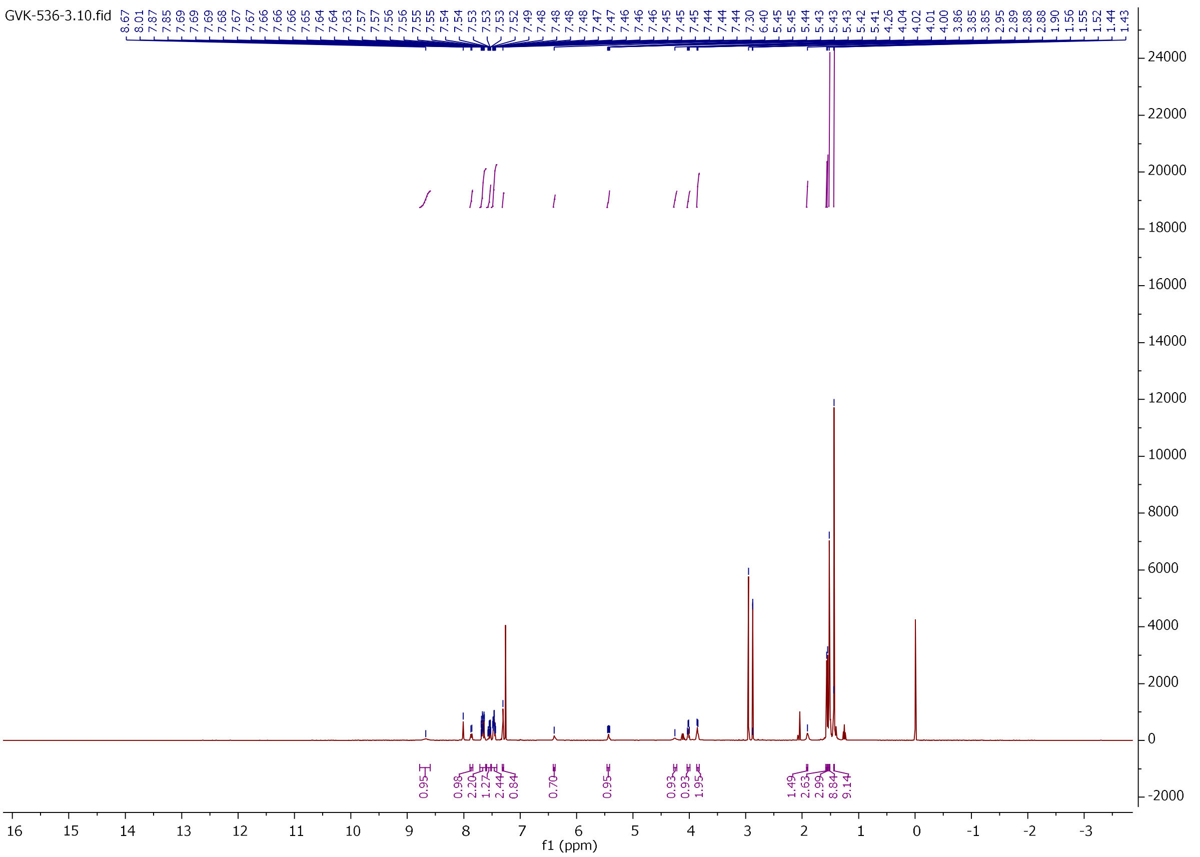
GVK-536-3.10.fid
f1 (ppm)

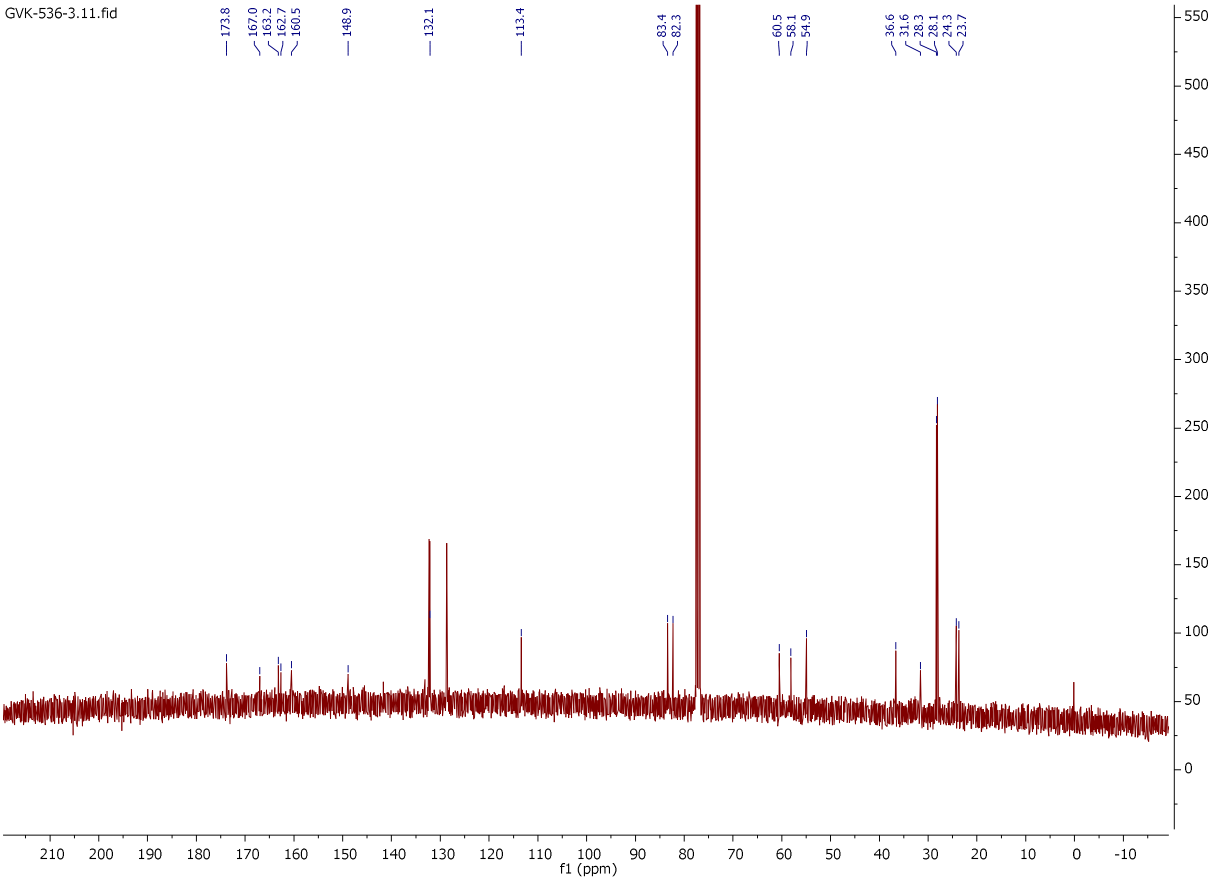
GVK-536-3.11.fid
173.8
167.0
163.2
162.7
160.5
148.9
132.1
113.4
83.4
82.3
60.5
58.1
54.9
36.6
31.6
28.3
28.1
24.3
23.7
f1 (ppm)

Compound **19**

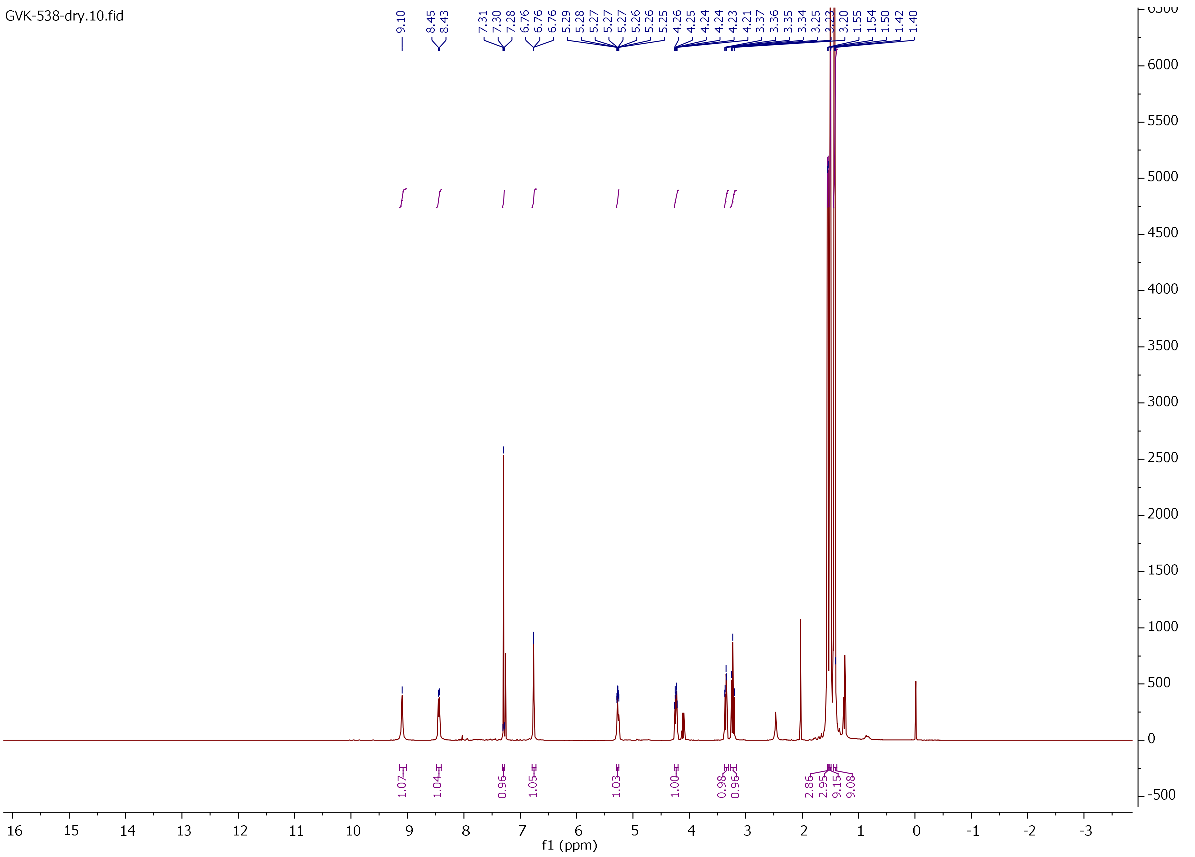

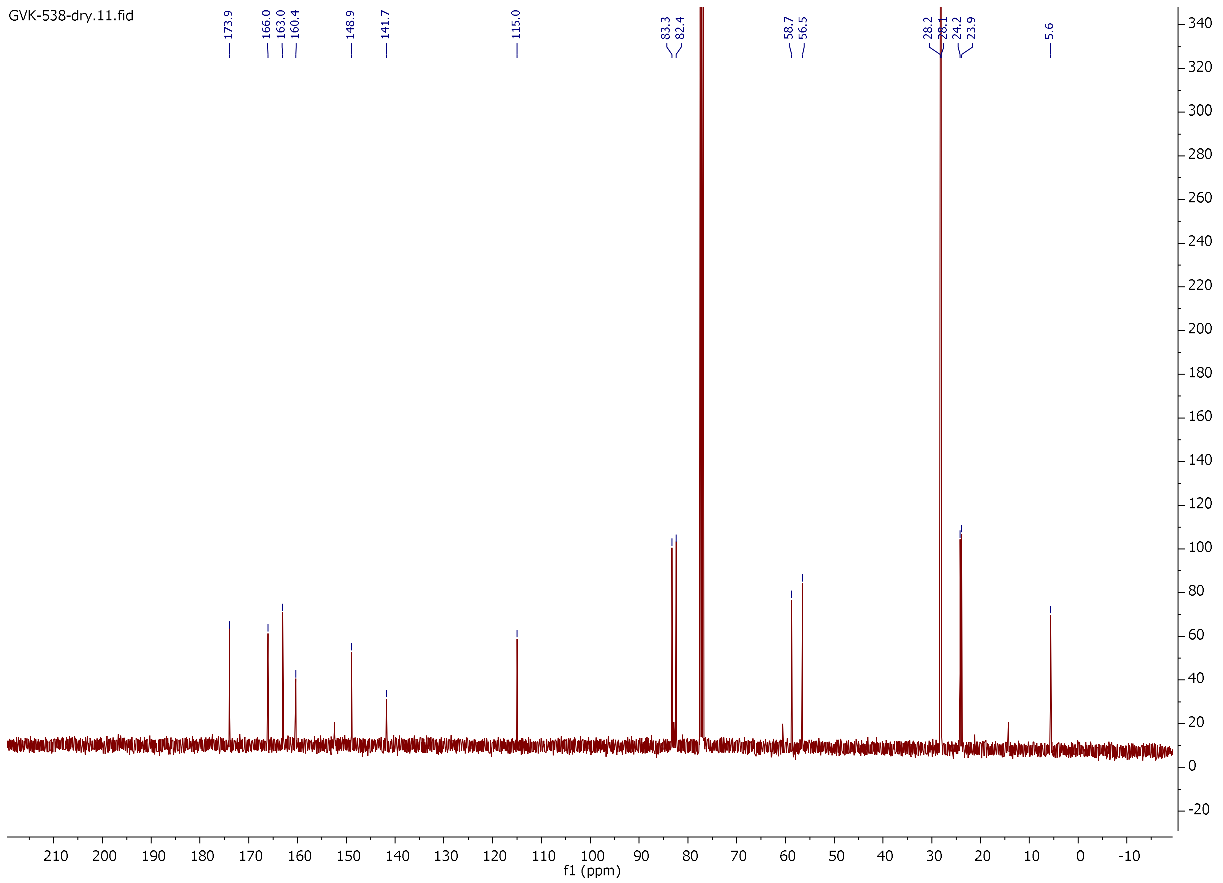
GVK-538-dry.11.fid
173.9
166.0
163.0
160.4
148.9
141.7
115.0
83.3
82.4
58.7
56.5
28.2
28.1
24.2
23.9
5.6
f1 (ppm)

Compound **20**

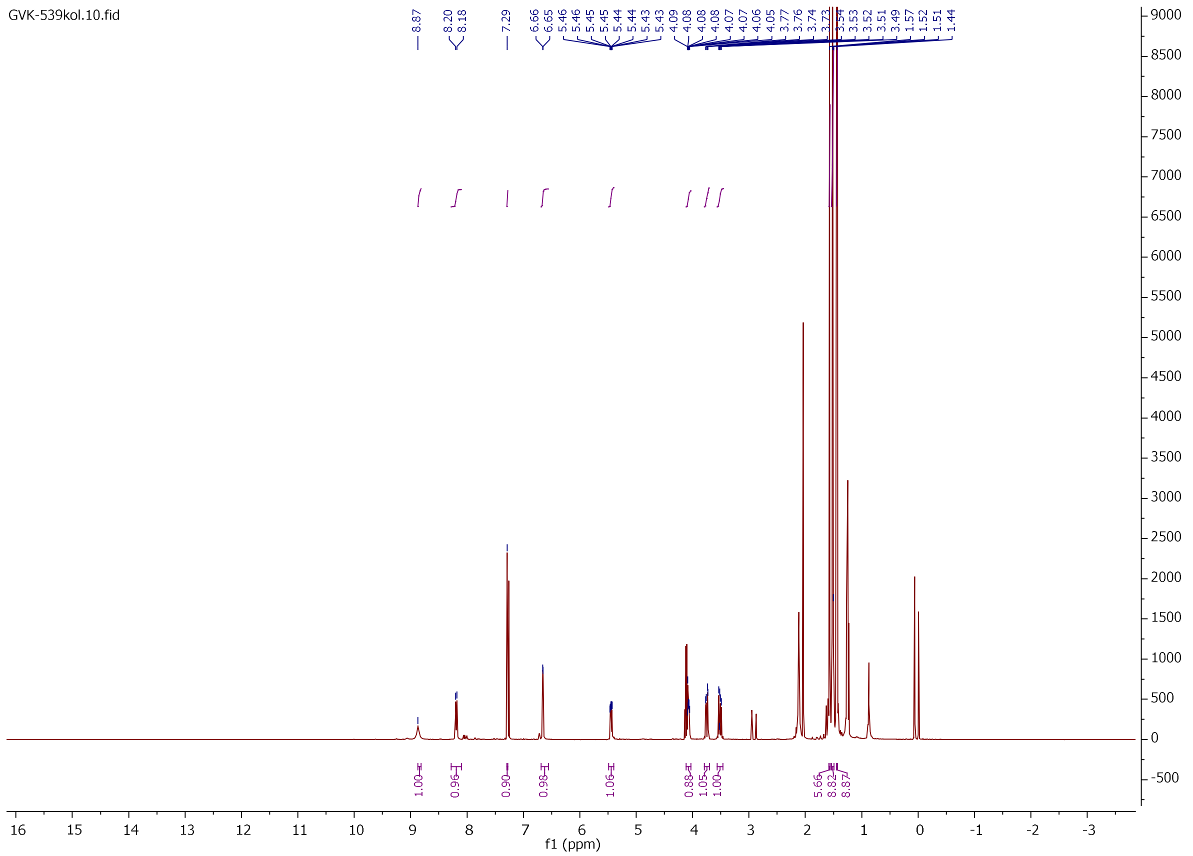
GVK-539kol.10.fid
8.87
8.20
8.18
7.29
6.66
6.65
5.46
5.45
5.45
5.44
5.44
5.43
5.43
4.09
4.08
4.08
4.07
4.07
4.06
4.05
3.77
3.76
3.74
3.73
3.54
3.53
3.52
3.51
3.49
1.57
1.52
1.51
1.44
1.00
0.96
0.90
0.98
1.06
0.88
1.05
1.00
5.66
8.82
8.87
f1 (ppm)

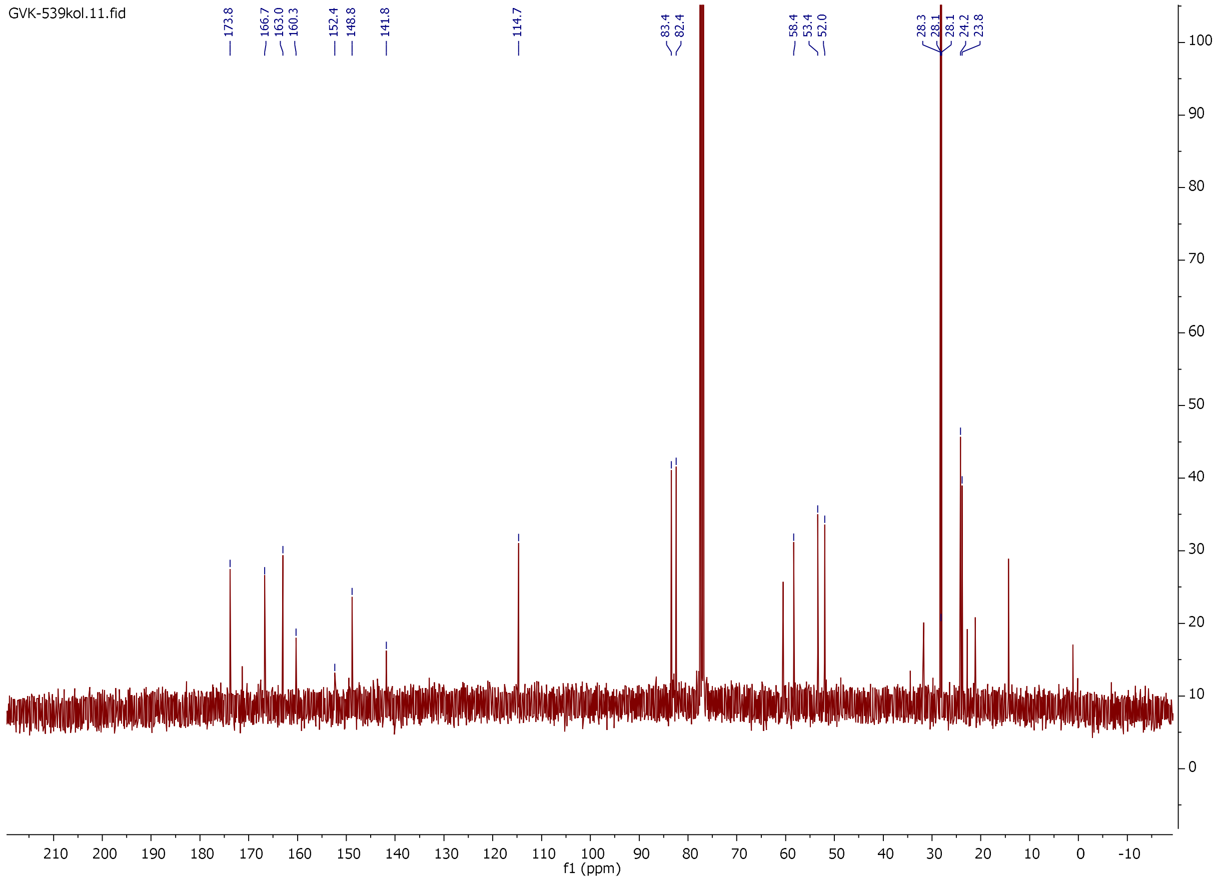
GVK-539kol.11.fid
173.8
166.7
163.0
160.3
152.4
148.8
141.8
114.7
83.4
82.4
58.4
53.4
52.0
28.3
28.1
28.1
24.2
23.8
f1 (ppm)

Compound **21**

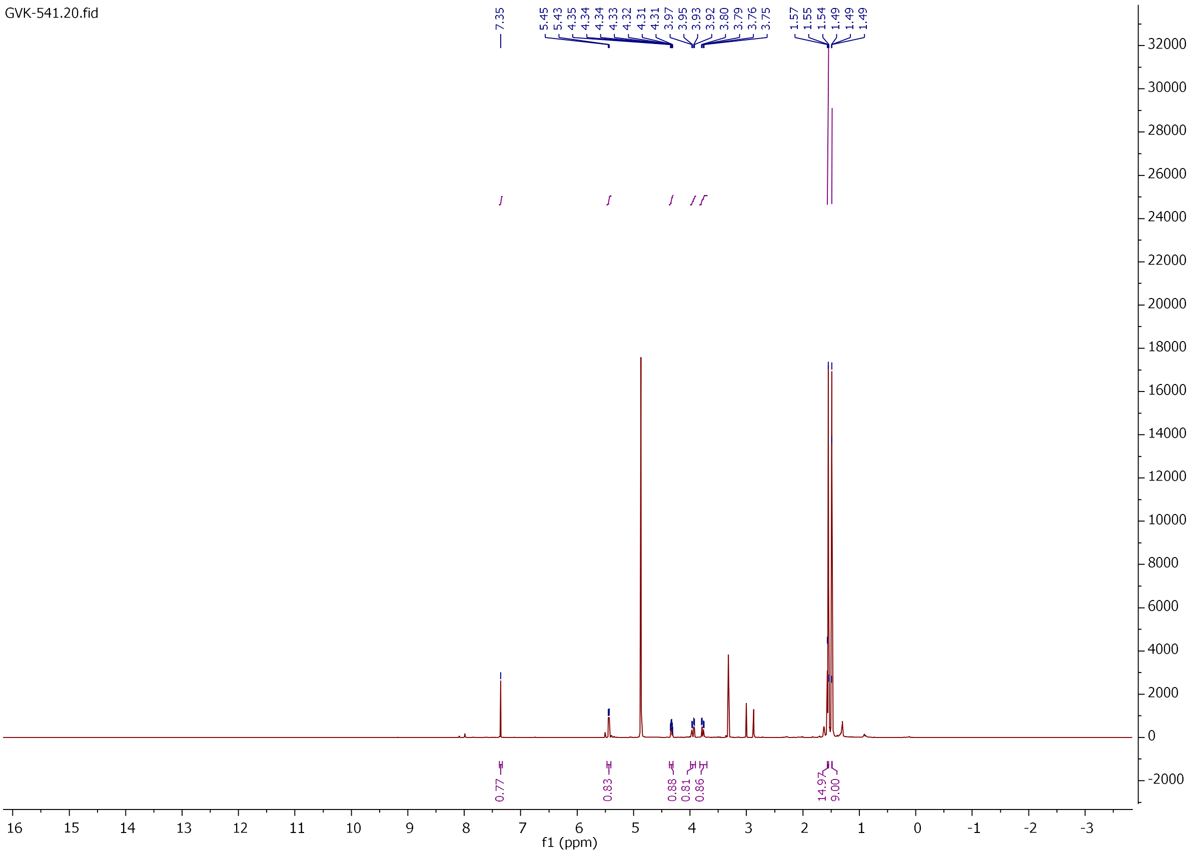
GVK-541.20.fid
7.35
5.45
5.43
4.35
4.34
4.34
4.33
4.32
4.31
4.31
3.97
3.95
3.93
3.92
3.80
3.79
3.76
3.75
1.57
1.55
1.54
1.49
1.49
1.49
0.77
0.83
0.88
0.81
0.86
14.97
9.00
f1 (ppm)

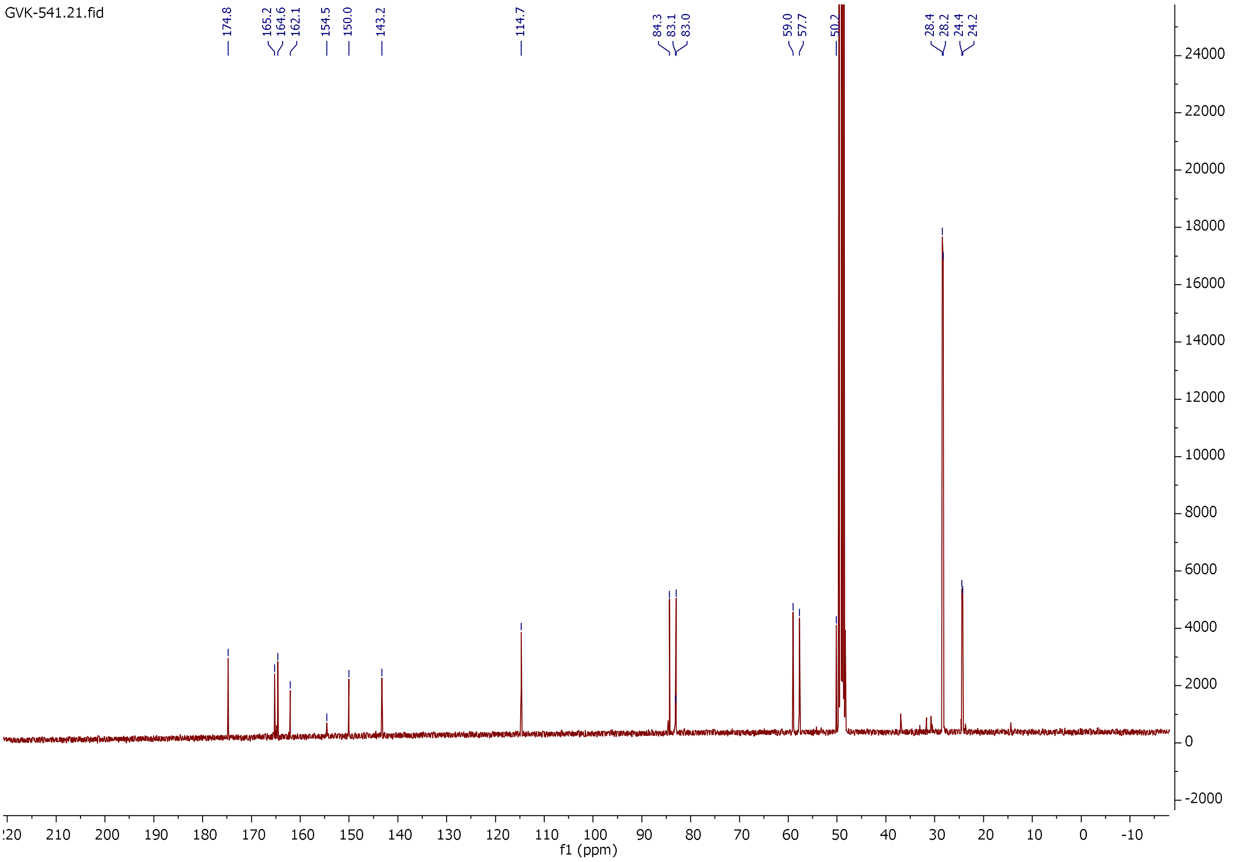
GVK-541.21.fid
174.8
165.2
164.6
162.1
154.5
150.0
143.2
114.7
84.3
83.1
83.0
59.0
57.7
50.2
28.4
28.2
24.4
24.2
f1 (ppm)

Compound **22**

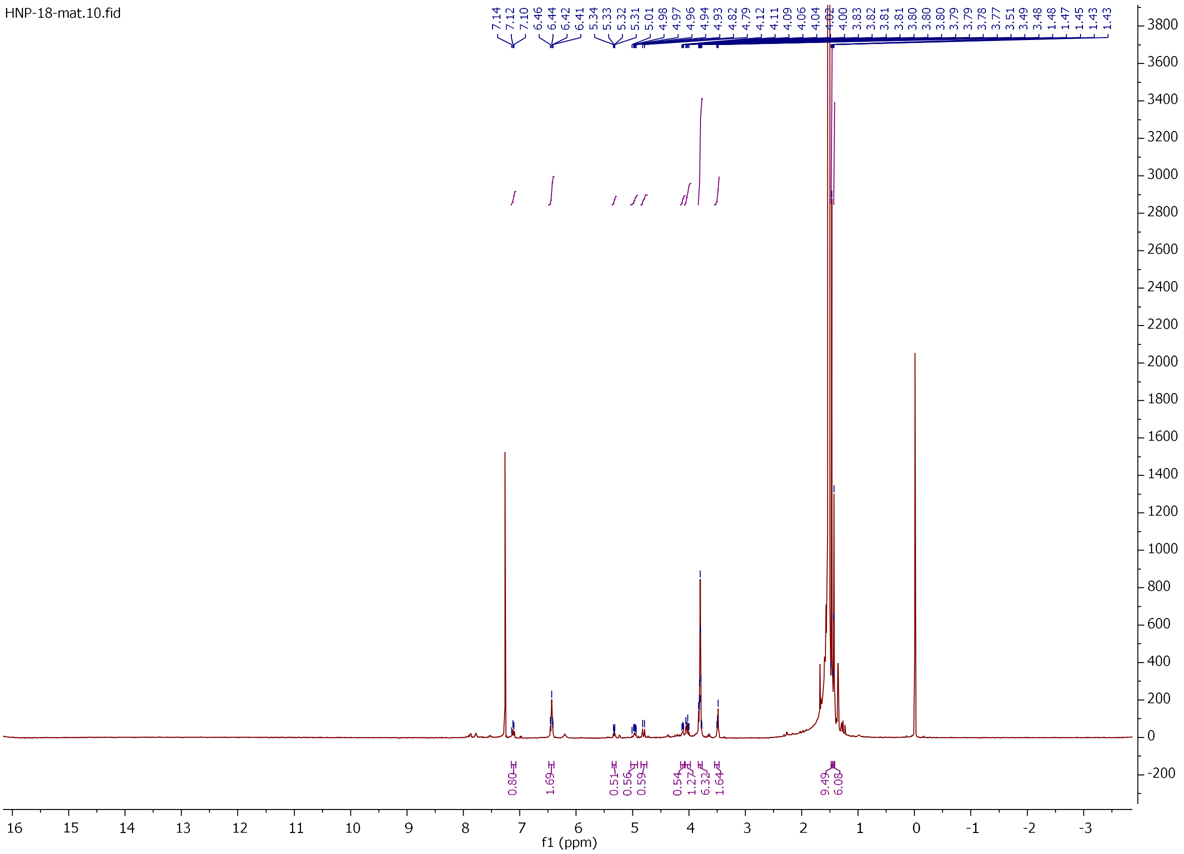

Compound **25**

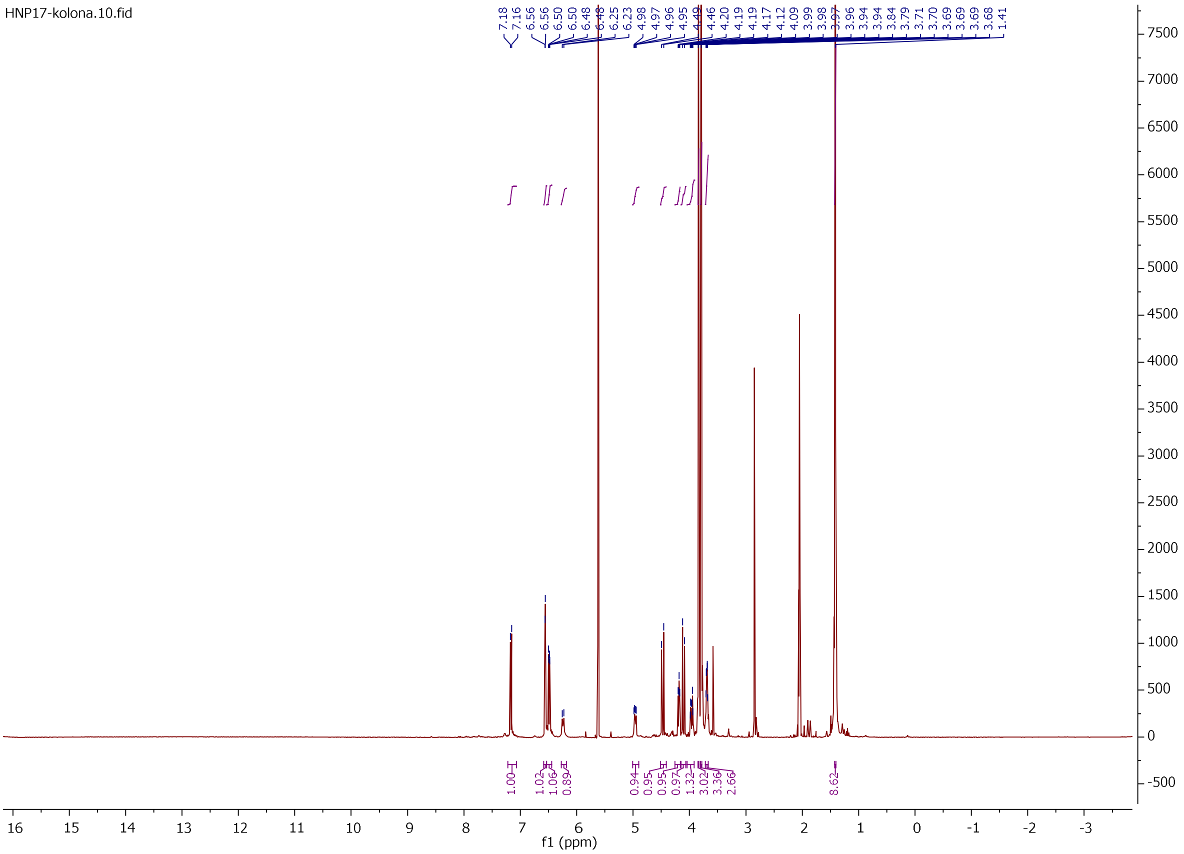

Compound **26**

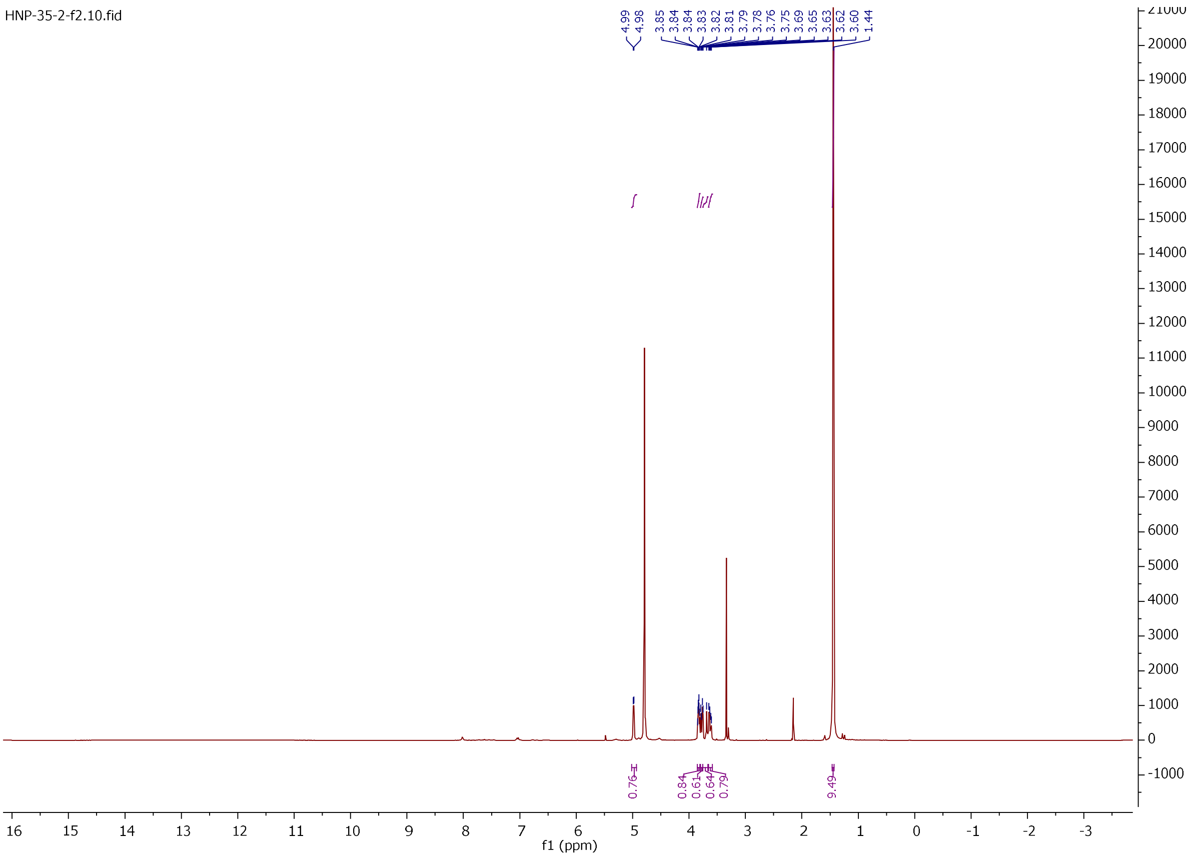

Compound **27a**

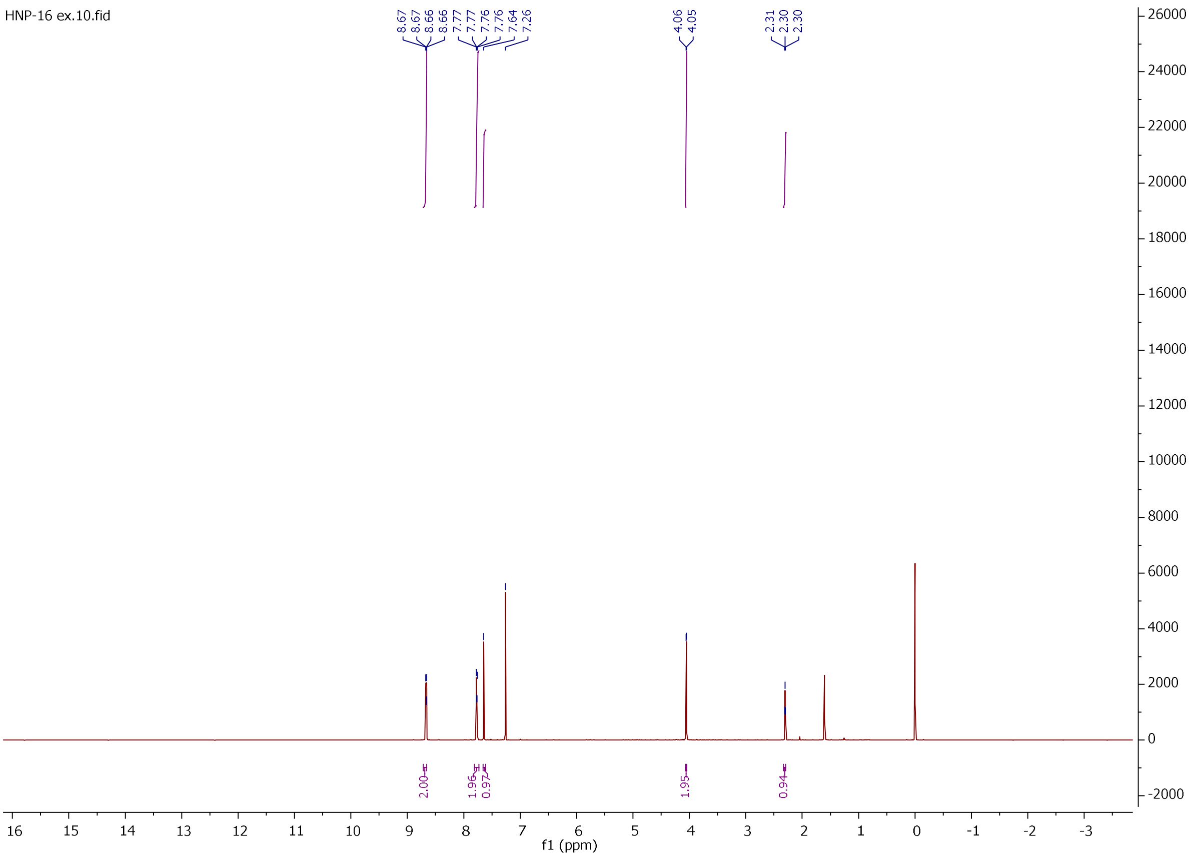

Compound **27**

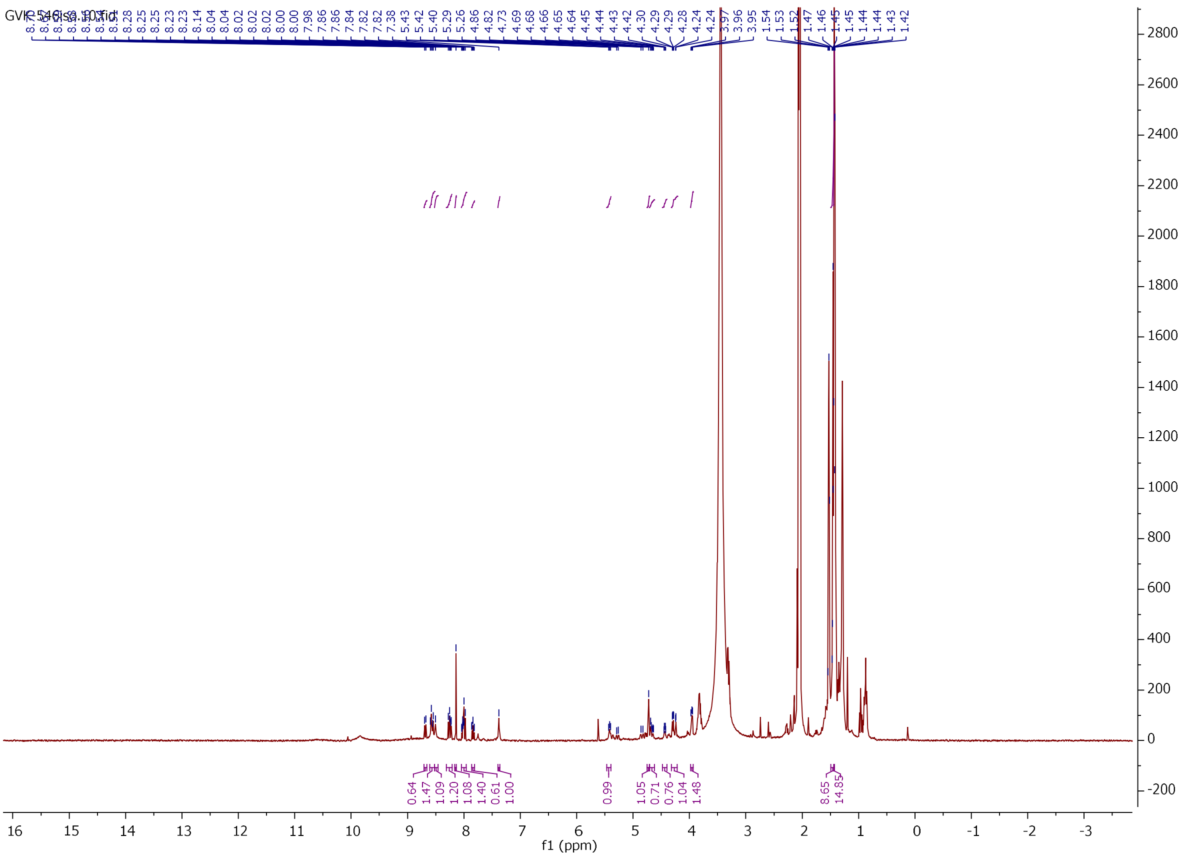

Compound **28**

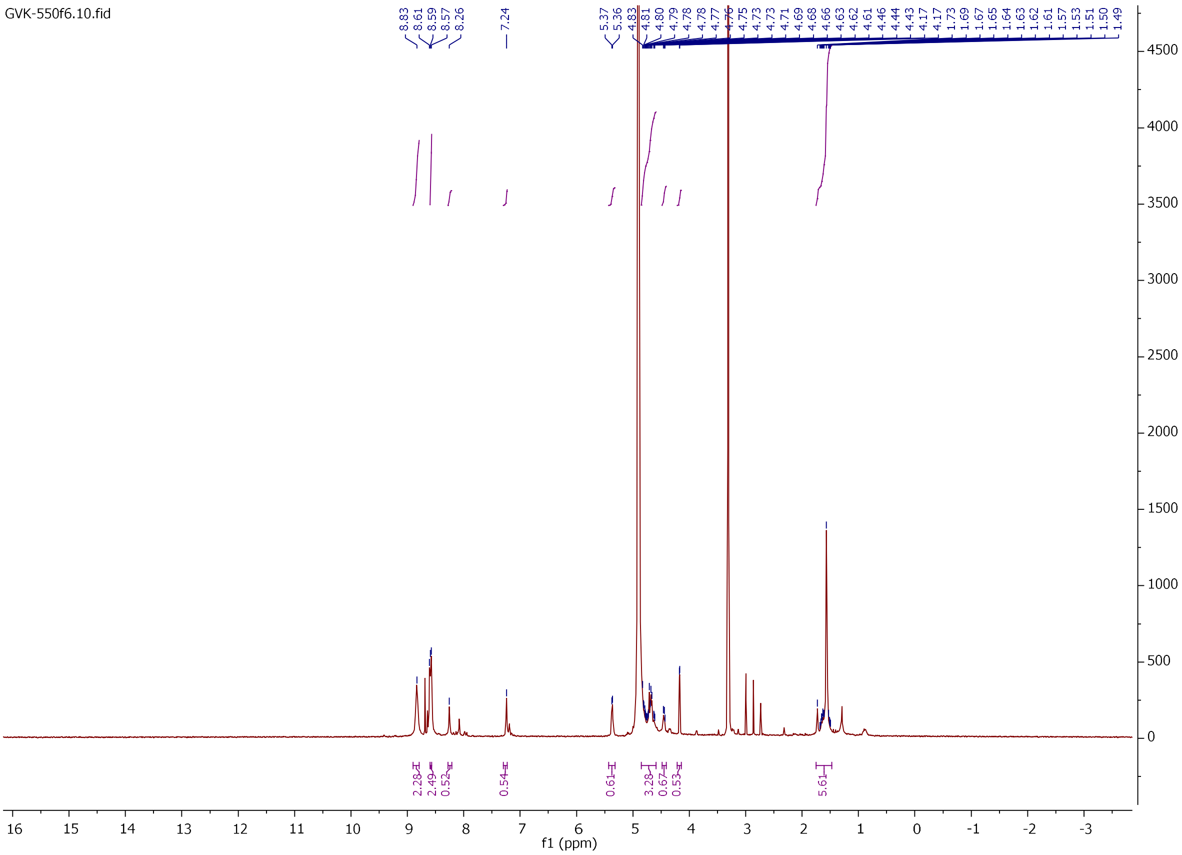

GVK-550_20260325223752 #751 RT: 6.47 AV: 1 NL: 1.26E7
T: FTMS + p ESI Full ms [200.0000-800.0000]

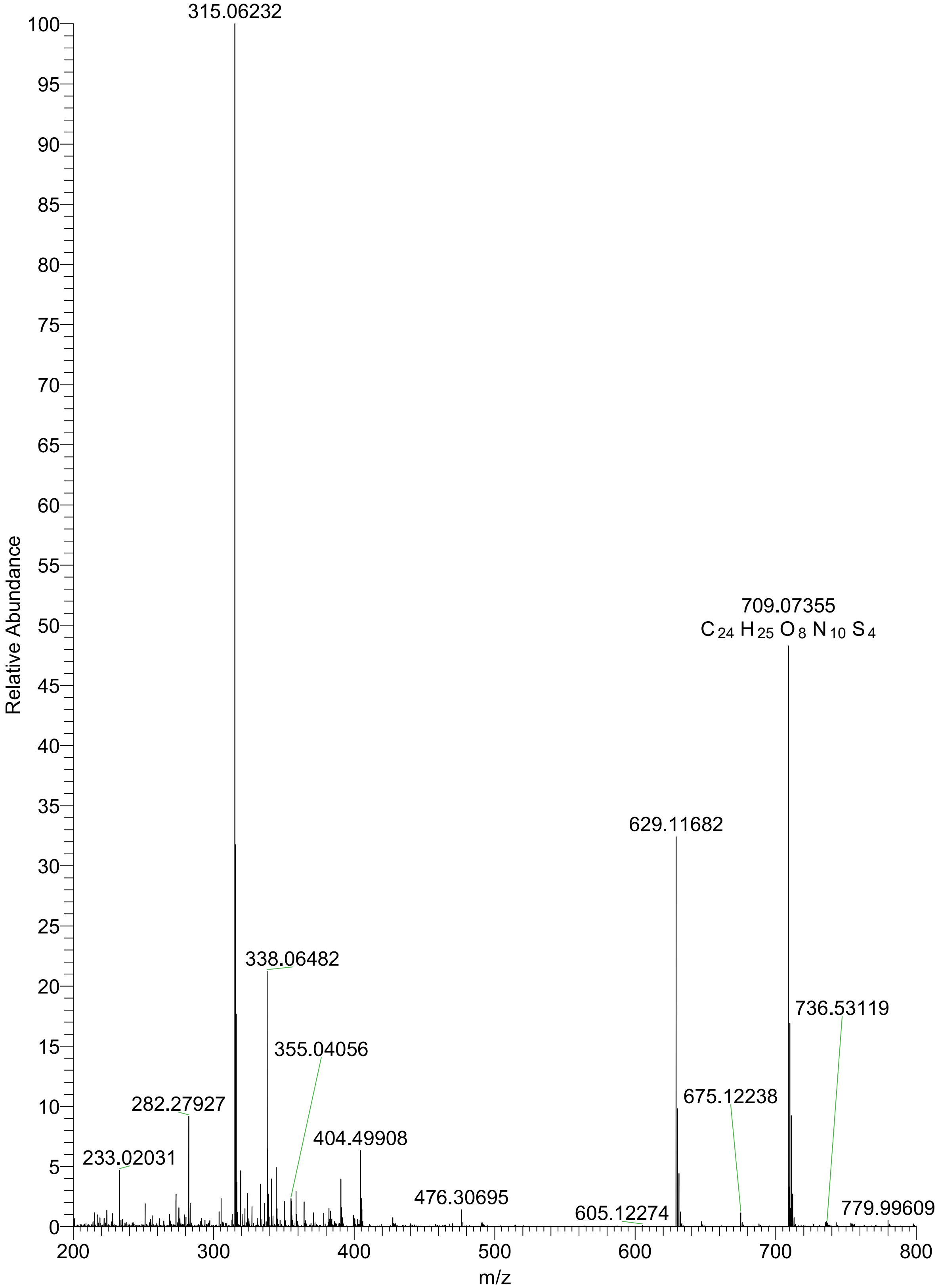

Elemental composition search on mass 709.07

m/z= 704.07-714.07

m/z	Theo. Mass	Delta (ppm)	RDB equiv.	Composition
709.07355	709.07342	0.19	17.5	$C_{24} H_{25} O_8 N_{10} S_4$

Chromatogram and Results

Injection Details

Injection Name:	GVK-550f6	*Run Time (min):*	8,00
Vial Number:	BE3	*Injection Volume:*	2,00
Injection Type:	Unknown	*Channel:*	UV_VIS_2
Calibration Level:		*Wavelength:*	254
Instrument Method:	General method (0.1TFA_MeCN_2-90)	*Bandwidth:*	16
Processing Method:	Quantitative	*Dilution Factor:*	1,0000
Injection Date/Time:	04.mar..26 13:12	*Sample Weight:*	1,0000

Chromatogram

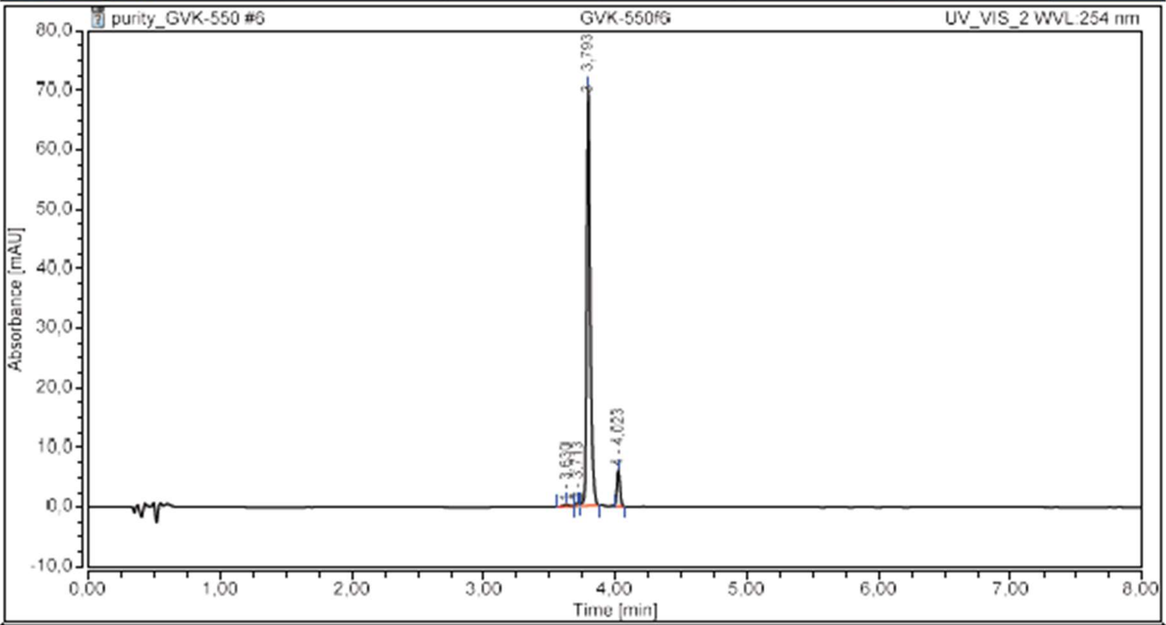

Integration Results

No.	Peak Name	Retention Time min	Area mAU*min	Height mAU	Relative Area %	Relative Height %	Amount n.a.
1		3,630	0,017	0,393	0,71	0,51	n.a.
2		3,713	0,017	0,479	0,70	0,62	n.a.
3		3,793	2,210	70,155	91,64	90,99	n.a.
4		4,023	0,168	6,078	6,96	7,88	n.a.
Total:			2,412	77,105	100,00	100,00	

Compound **29a**

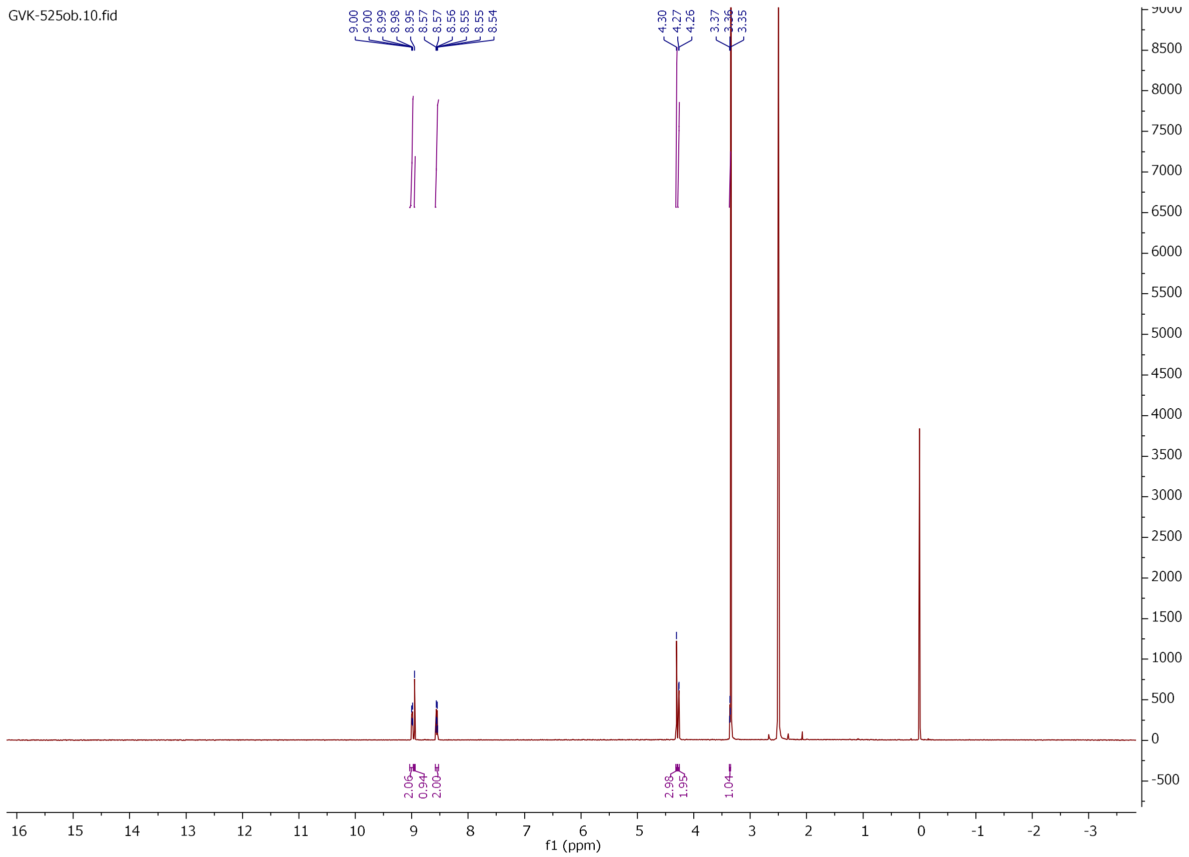

Compound **30**

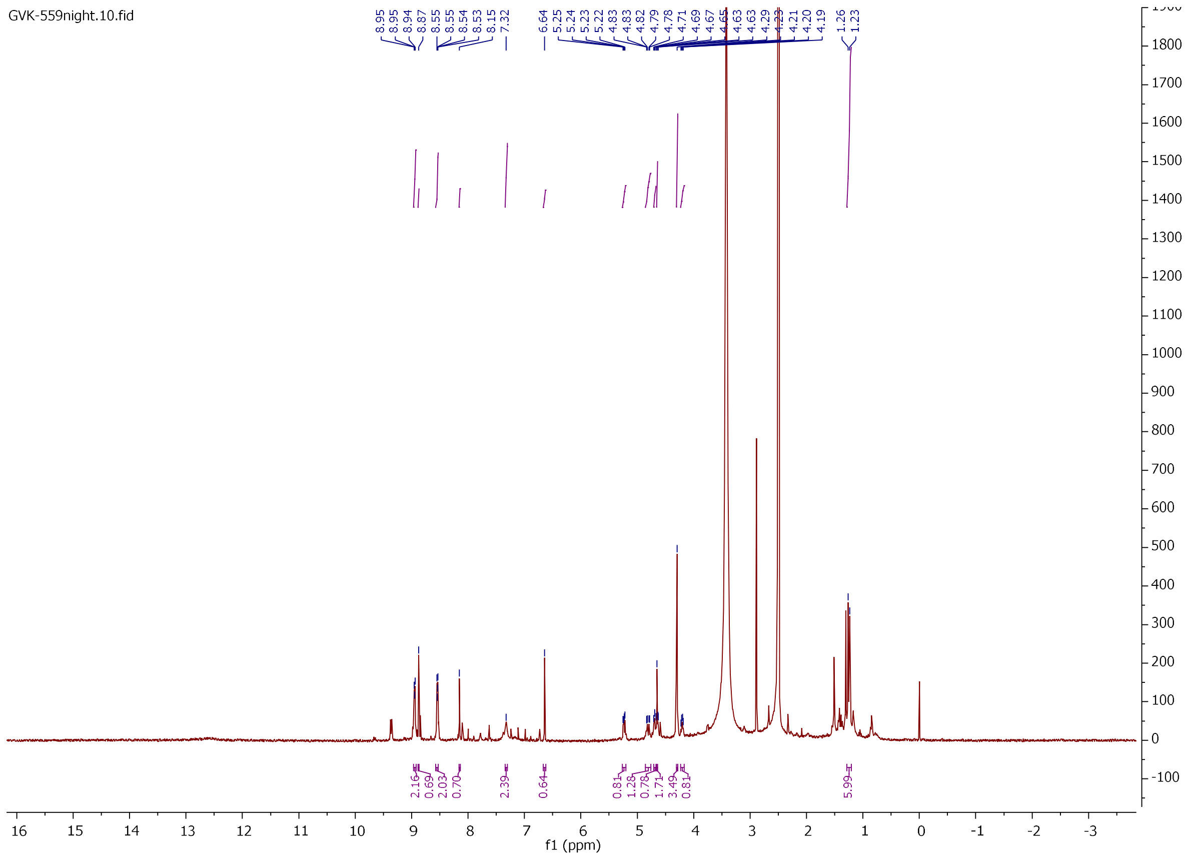

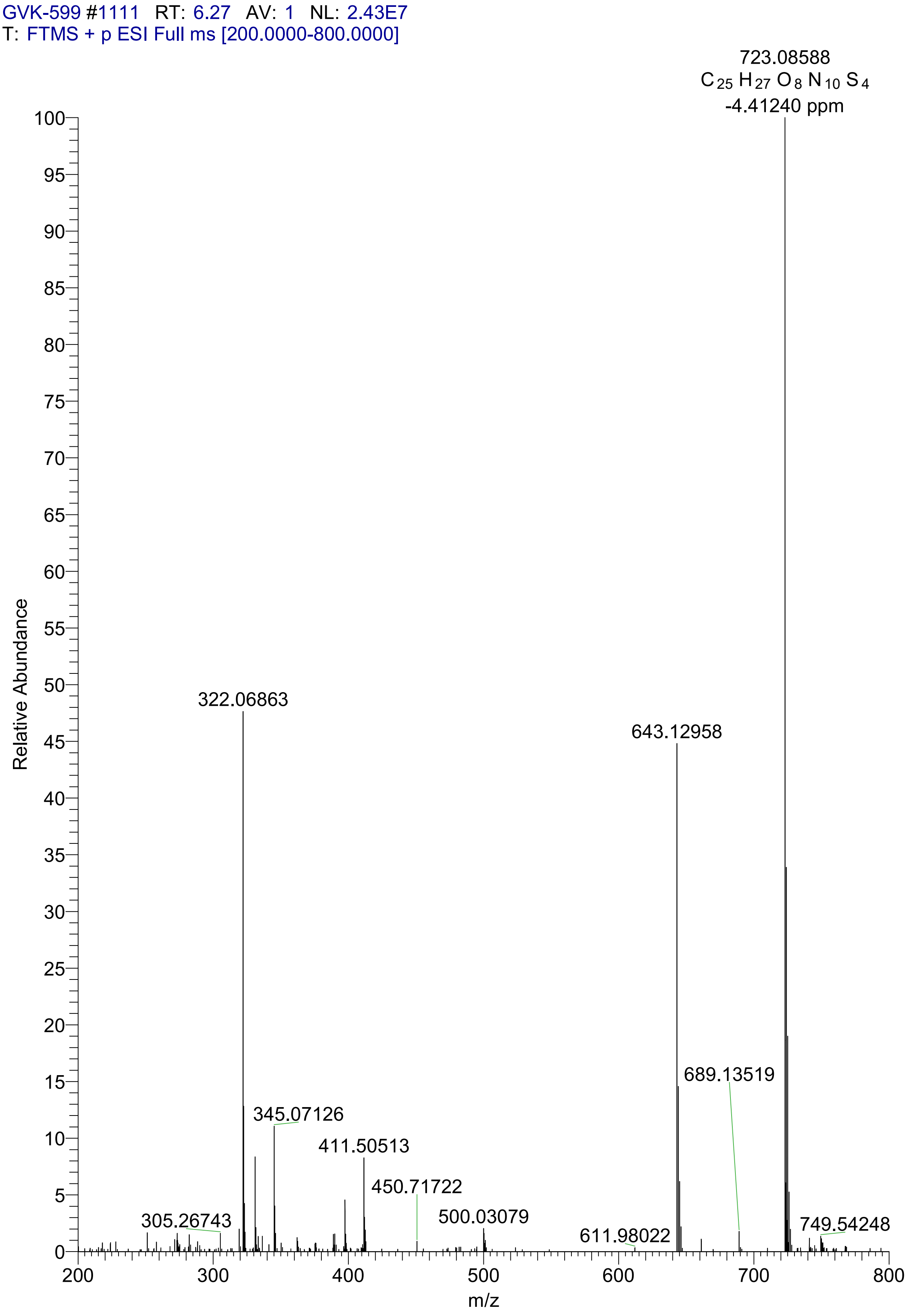
GVK-599 #1111 RT: 6.27 AV: 1 NL: 2.43E7
T: FTMS + p ESI Full ms [200.0000-800.0000]
723.08588
C 25 H 27 O 8 N 10 S 4
-4.41240 ppm
322.06863
643.12958
689.13519
345.07126
411.50513
450.71722
305.26743
500.03079
611.98022
749.54248
Relative Abundance
m/z

Elemental composition search on mass 723.09

m/z= 718.09-728.09

m/z	Theo. Mass	Delta (ppm)	RDB equiv.	Composition
723.08588	723.08907	-4.41	17.5	$C_{25} H_{27} O_{8} N_{10} S_{4}$

Chromatogram and Results

Injection Details

Injection Name:	**GVK-559f5**	*Run Time (min):*	**8,00**
Vial Number:	**GA4**	*Injection Volume:*	**2,00**
Injection Type:	**Unknown**	*Channel:*	**UV_VIS_2**
Calibration Level:		*Wavelength:*	**254**
Instrument Method:	**General method (0.1TFA_MeCN_10-90)**	*Bandwidth:*	**16**
Processing Method:	**Quantitative**	*Dilution Factor:*	**1,0000**
Injection Date/Time:	**07.maj.26 12:15**	*Sample Weight:*	**1,0000**

Chromatogram

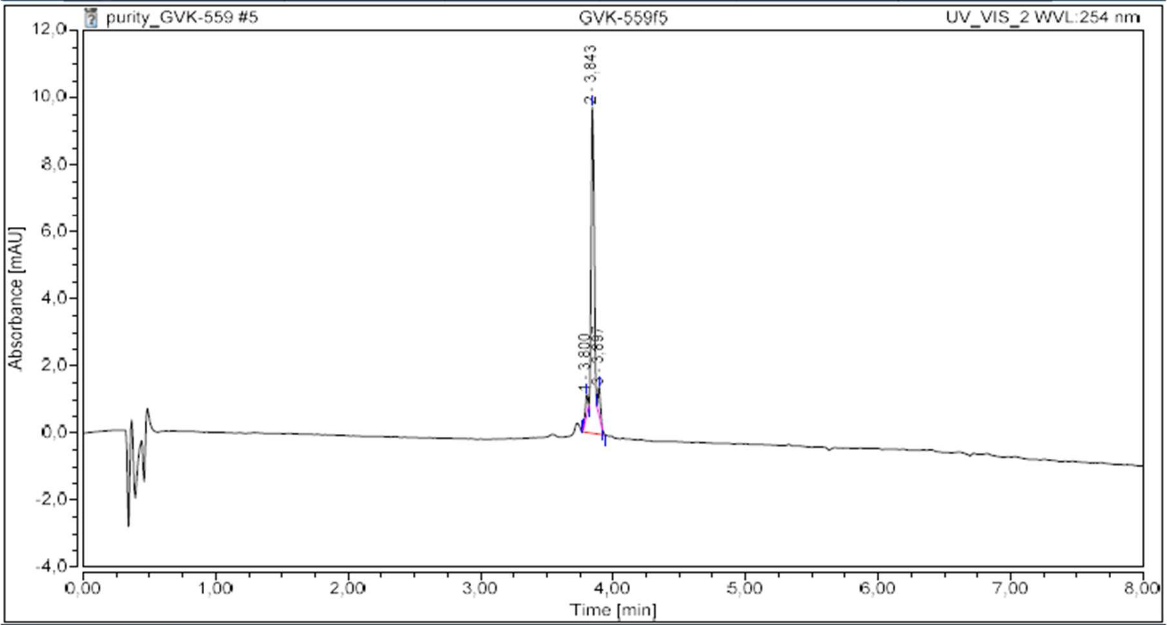

Integration Results

No.	Peak Name	Retention Time min	Area mAU*min	Height mAU	Relative Area %	Relative Height %	Amount n.a.
1		3,800	0,013	0,599	3,67	5,36	n.a.
2		3,843	0,321	9,731	91,23	87,09	n.a.
3		3,897	0,018	0,844	5,09	7,55	n.a.
**Total:**			**0,352**	**11,173**	**100,00**	**100,00**	

Compound **31**

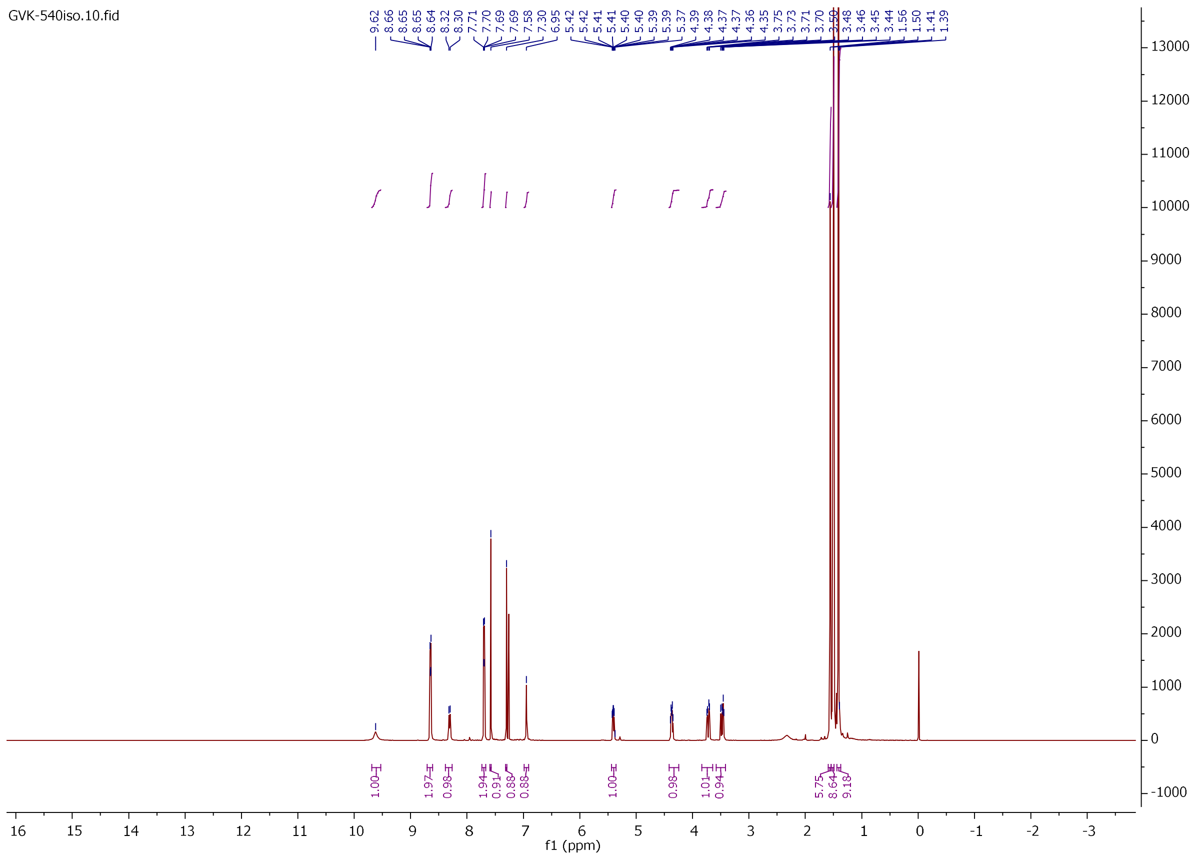
GVK-540iso.10.fid
9.62
8.66
8.65
8.65
8.64
8.32
8.30
7.71
7.70
7.69
7.69
7.58
7.30
6.95
5.42
5.42
5.41
5.41
5.40
5.40
5.39
5.39
5.37
4.39
4.38
4.37
4.37
4.36
4.35
3.75
3.73
3.71
3.70
3.50
3.48
3.46
3.45
3.44
1.56
1.50
1.41
1.39
1.00
1.97
0.98
1.94
0.91
0.88
0.88
1.00
0.98
1.01
0.94
5.75
8.64
9.18
f1 (ppm)

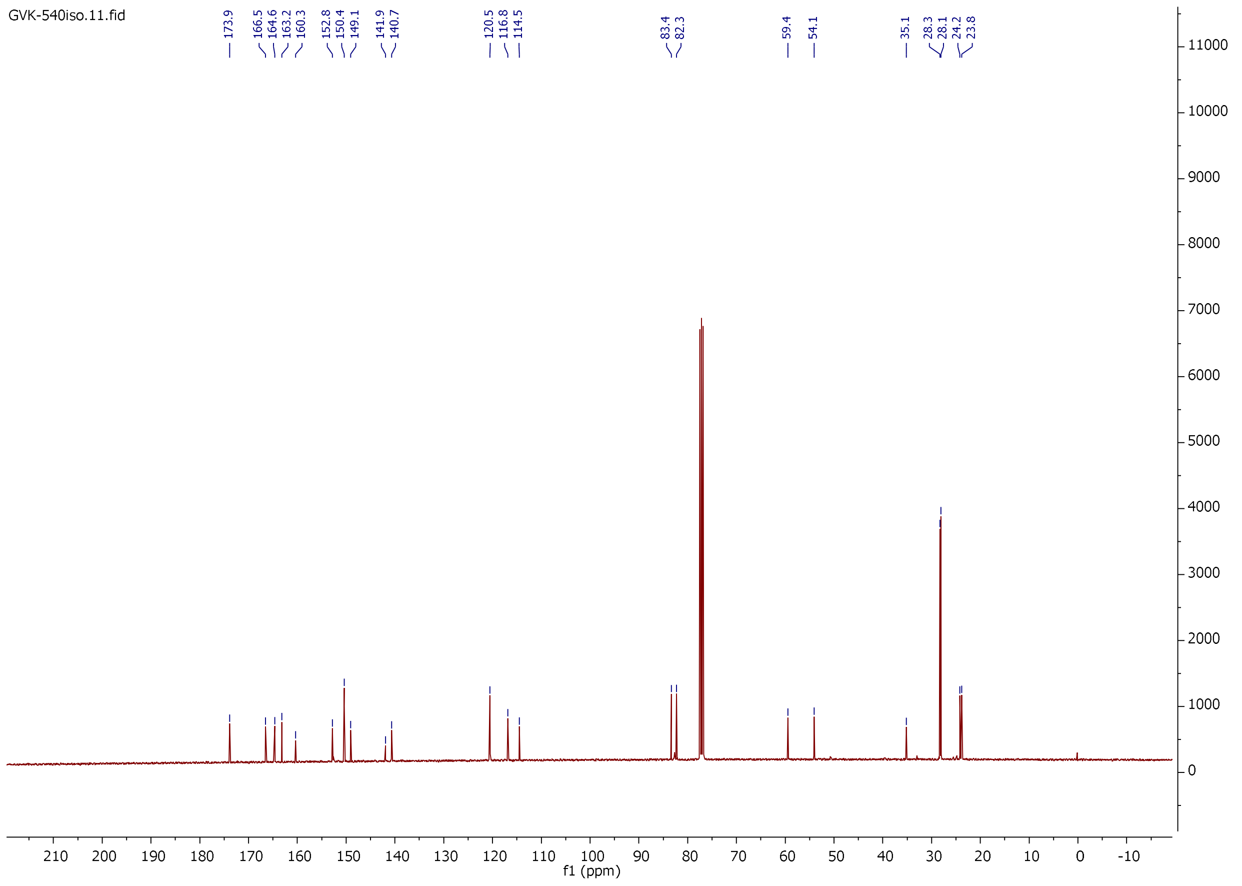
GVK-540iso.11.fid
173.9
166.5
164.6
163.2
160.3
152.8
150.4
149.1
141.9
140.7
120.5
116.8
114.5
83.4
82.3
59.4
54.1
35.1
28.3
28.1
24.2
23.8
f1 (ppm)

Compound **32**

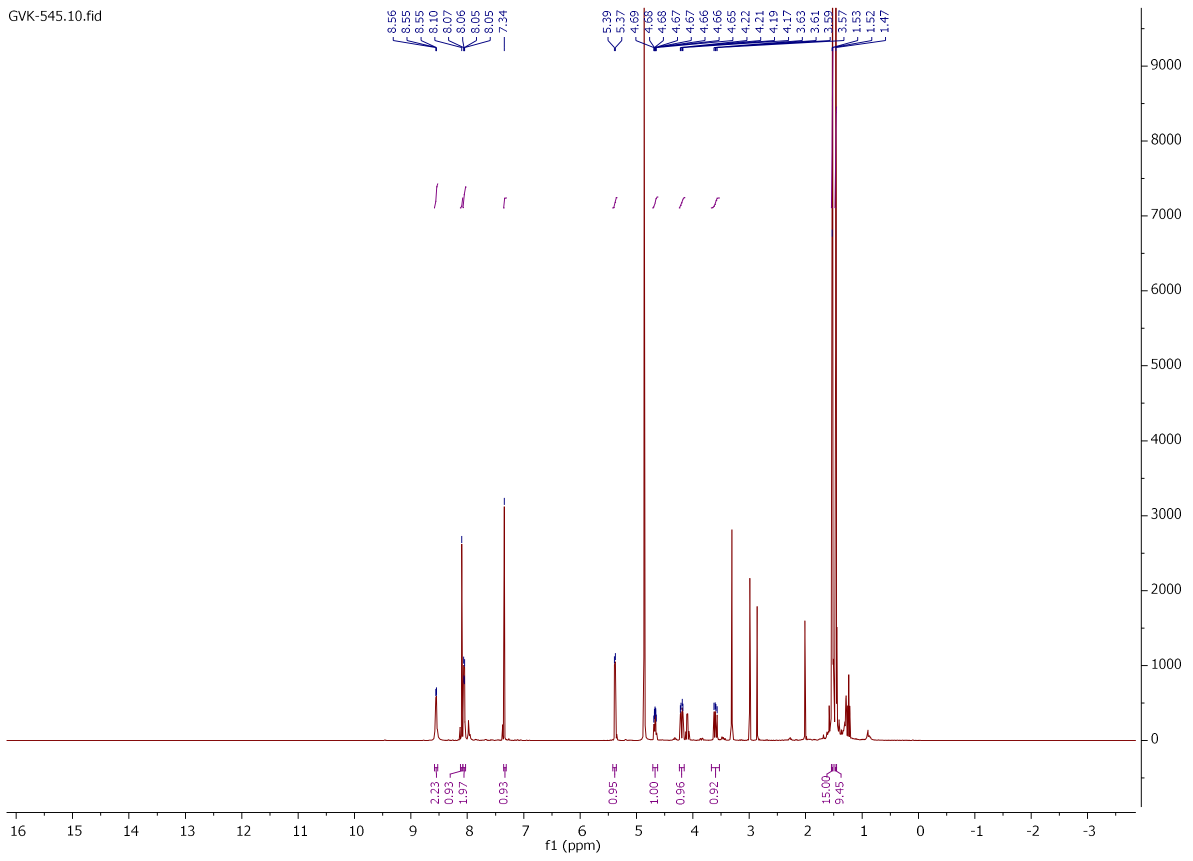
GVK-545.10.fid
8.56
8.55
8.55
8.10
8.07
8.06
8.05
8.05
7.34
5.39
5.37
4.69
4.68
4.68
4.67
4.67
4.66
4.66
4.65
4.22
4.21
4.19
4.17
3.63
3.61
3.59
3.57
1.53
1.52
1.47
2.23
0.93
1.97
0.93
0.95
1.00
0.96
0.92
15.00
9.45
f1 (ppm)

Compound **33**

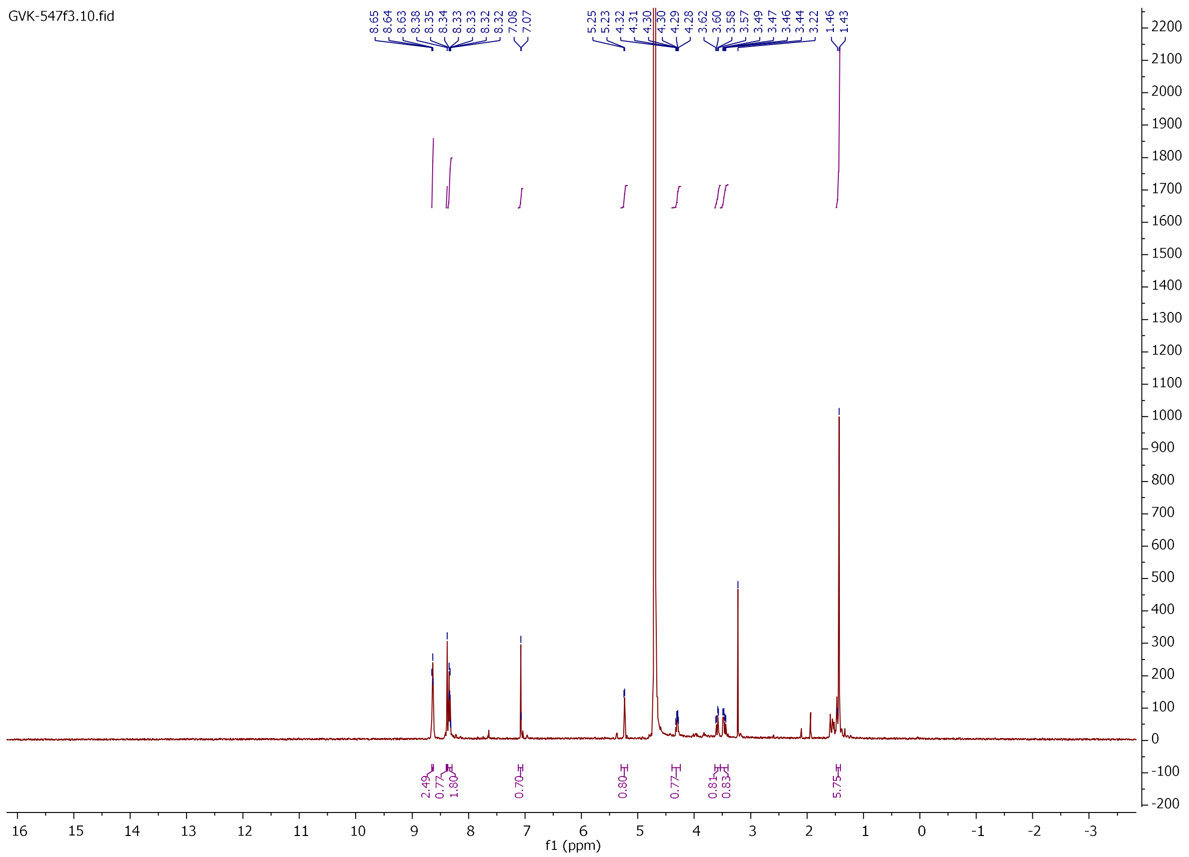

GVK-547f3.10.fid
8.65
8.64
8.63
8.38
8.35
8.34
8.33
8.33
8.32
8.32
7.08
7.07
5.25
5.23
4.32
4.31
4.30
4.30
4.29
4.28
3.62
3.60
3.58
3.57
3.49
3.47
3.46
3.44
3.22
1.46
1.43
2.49
0.77
1.80
0.70
0.80
0.77
0.81
0.83
5.75
f1 (ppm)

S

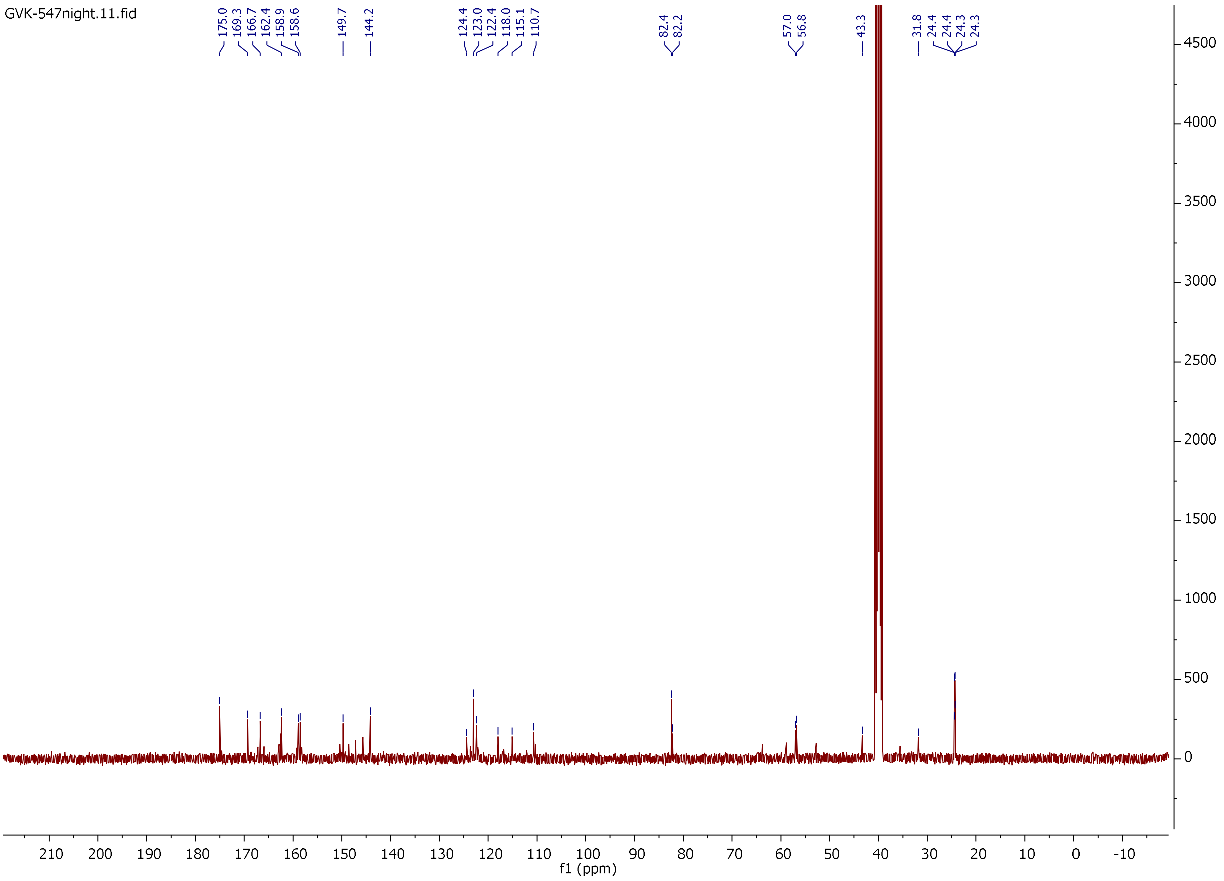

GVK-547night.11.fid
175.0
169.3
166.7
162.4
158.9
158.6
149.7
144.2
124.4
123.0
122.4
118.0
115.1
110.7
82.4
82.2
57.0
56.8
43.3
31.8
24.4
24.4
24.3
24.3
4500
4000
3500
3000
2500
2000
1500
1000
500
0
210
200
190
180
170
160
150
140
130
120
110
100
90
80
70
60
50
40
30
20
10
0
-10
f1 (ppm)

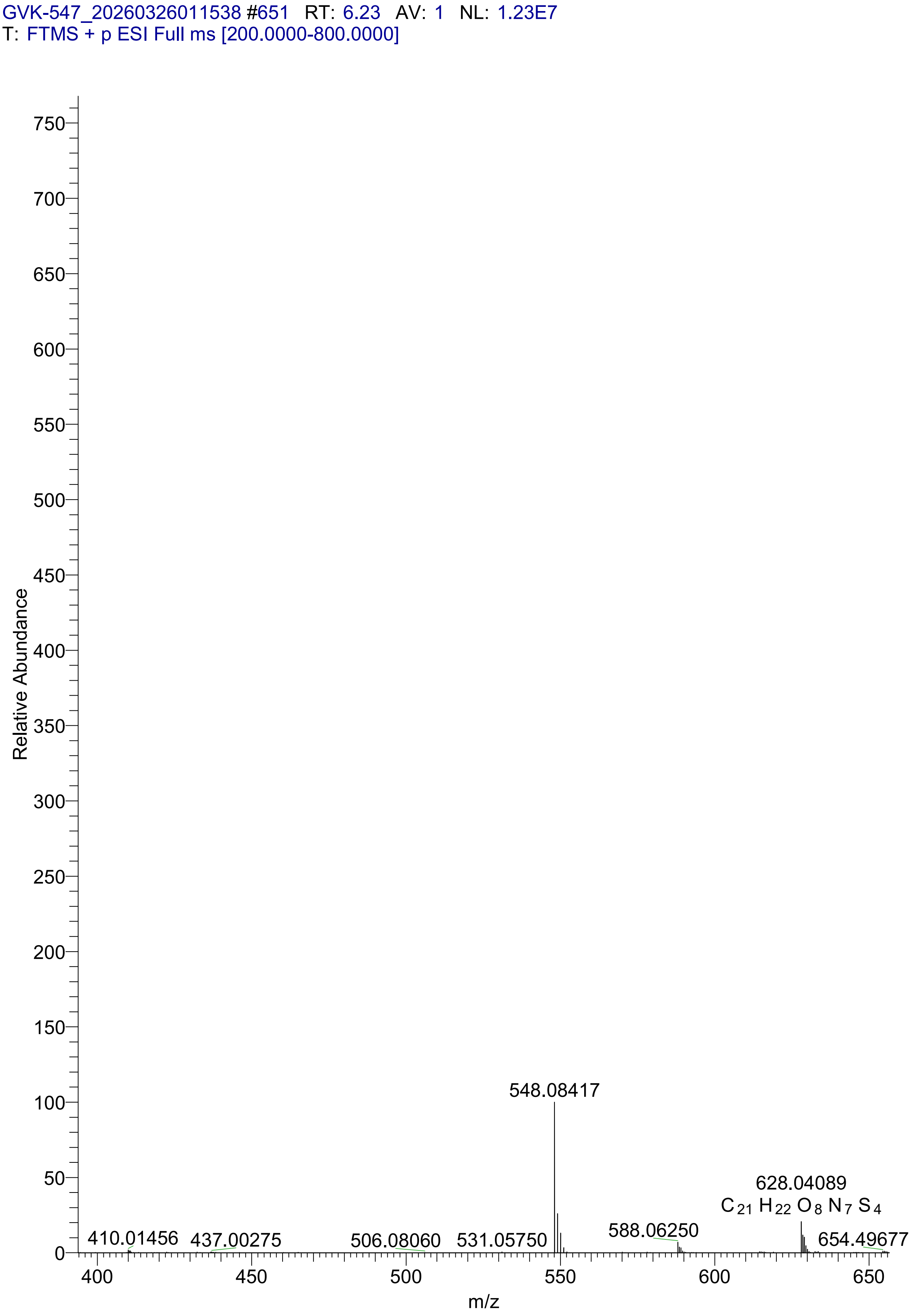
GVK-547_20260326011538 #651 RT: 6.23 AV: 1 NL: 1.23E7
T: FTMS + p ESI Full ms [200.0000-800.0000]
Relative Abundance
m/z
548.08417
628.04089
C 21 H 22 O 8 N 7 S 4
410.01456
437.00275
506.08060
531.05750
588.06250
654.49677

Elemental composition search on mass 628.04

m/z= 623.04-633.04

m/z	Theo. Mass	Delta (ppm)	RDB equiv.	Composition
628.04089	628.04072	0.27	14.5	$C_{21} H_{22} O_8 N_7 S_4$

Chromatogram and Results

Injection Details

Injection Name:	**GVK-547-2iso**	*Run Time (min):*	**8,00**
Vial Number:	**BE1**	*Injection Volume:*	**5,00**
Injection Type:	**Unknown**	*Channel:*	**UV_VIS_2**
Calibration Level:		*Wavelength:*	**254**
Instrument Method:	**General method (0.1TFA_MeCN_2-90)**	*Bandwidth:*	**16**
Processing Method:	**Quantitative**	*Dilution Factor:*	**1,0000**
Injection Date/Time:	**19.feb..26 15:12**	*Sample Weight:*	**1,0000**

Chromatogram

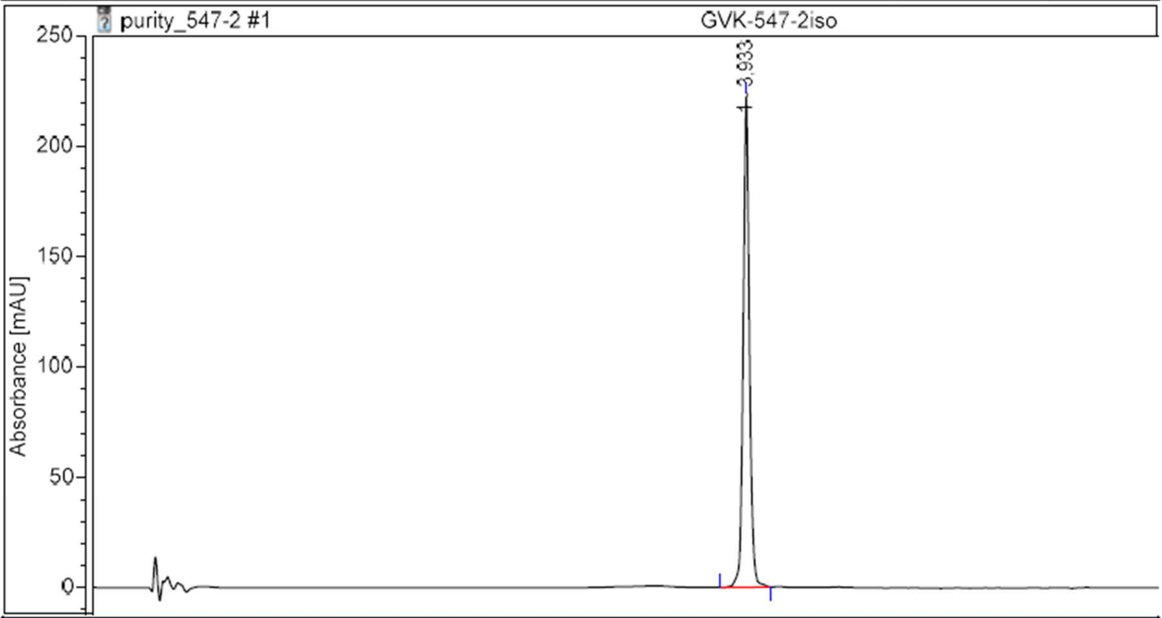

Integration Results

No.	Peak Name	Retention Time min	Area mAU*min	Height mAU	Relative Area %	Relative Height %	Amount n.a.
1		3,933	9,667	222,448	100,00	100,00	n.a.
**Total:**			**9,667**	**222,448**	**100,00**	**100,00**	
